# From neurotoxicity to neuroprotection: Rethinking GABA_A_R-targeting anesthetics

**DOI:** 10.1007/s10565-025-10057-z

**Published:** 2025-06-14

**Authors:** Yubao Li, Hongliang Yang, Lu Liu, Lulu Jiang, Peilin Xie, Xiaoling Wang, Xuhui Cong, Ruilou Zhu, Zhongyuan Lu, Mingyang Sun, Jiaqiang Zhang

**Affiliations:** 1 Xinxiang Medical University, Xinxiang, Henan China; 2 Department of Anesthesiology and Perioperative Medicine, Zhengzhou University People’s Hospital and Henan Provincial People’s Hospital, Zhengzhou, Henan China; 3 Academy of Medical Science, Zhengzhou University, Zhengzhou, Henan China; 4 Institute of ElectrophysiologyHenan Academy of Innovations in Medical Science, Zhengzhou, China

**Keywords:** Anesthetics targeting GABAARs, Neurodevelopment, Neurotoxicity, Neuroprotective, Neuroactive steroids

## Abstract

The brain growth spurt (BGS) represents a pivotal window in neurodevelopment, defined by rapid neurogenesis, heightened synaptogenesis, and the dynamic establishment of neural networks. During this phase, heightened brain plasticity significantly enhances learning and memory abilities, while also increasing the brain’s susceptibility to disruptions. Anesthetics, particularly those targeting γ-aminobutyric acid type A receptors (GABA_A_Rs), interfere with GABAergic and glutamatergic systems, disrupt brain-derived neurotrophic factor (BDNF) signaling, and exacerbate neurotoxic effects. These agents activate glial cells, induce inflammation, and contribute to oxidative stress, while also disrupting calcium homeostasis and triggering endoplasmic reticulum stress. Furthermore, anesthetics alter the expression of non-coding RNAs, which affects gene regulation and long-term memory formation. The extent of neurotoxic effects is contingent upon a constellation of factors, including the timing, dosage, and frequency of anesthetic exposure, as well as individual susceptibility. Notably, perioperative administration of anesthetic agents has been implicated in long-term cognitive dysfunction, thereby emphasizing the critical importance of precisely modulated dosing regimens and temporally optimized delivery strategies to mitigate potential neurodevelopmental risks. In contrast, neuroactive steroids demonstrate promising neuroprotective potential by modulating GABA_A_R activity, enhancing BDNF release, and regulating oxidative stress and inflammation. New strategies for preventing and reversing anesthetic-induced neurotoxicity could include novel anesthetic combinations, anti-apoptotic agents, antioxidants, or nutritional supplements. These findings underscore the complex and multifactorial effects of anesthetic agents on the developing brain and emphasize the urgent need to establish and refine anesthetic strategies that safeguard neural integrity during vulnerable windows of neurodevelopment.

**Graphical Abstract:**

Mechanisms of Anesthetic-Induced Neurotoxicity Targeting GABA_A_Rs and Associated Susceptibility Factors. Neonatal brain development occurs during a critical period, and anesthetic-induced neurotoxicity during this time is influenced by multiple complex factors, including the mechanisms of anesthetic drugs targeting GABA receptors and associated susceptibility factors. The interplay of these factors contributes to the formation of a complex mechanism underlying neonatal neurotoxicity induced by anesthesia

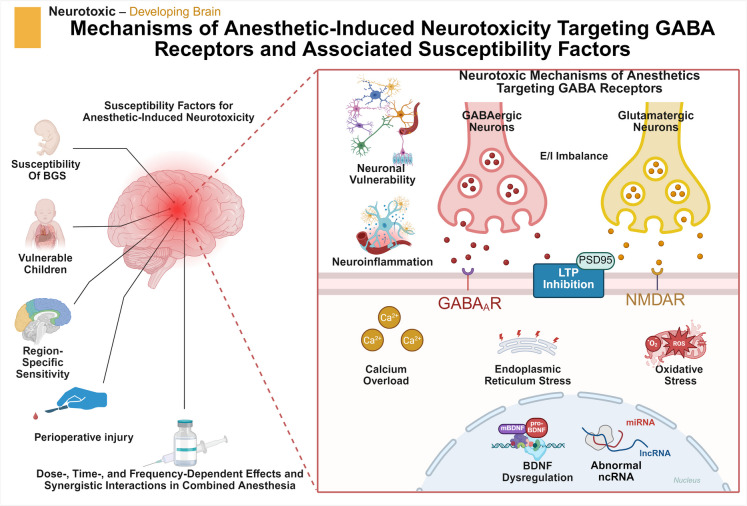

**Supplementary Information:**

The online version contains supplementary material available at 10.1007/s10565-025-10057-z.

## Introduction

A growing body of evidence derived from both animal models and clinical research suggests that the postnatal BGS represents a critical period in neurodevelopment, during which the central nervous system displays exceptional plasticity and heightened susceptibility to external perturbations. During this critical developmental window, neuronal circuits are sculpted through the rapid generation and remodeling of trillions of synapses, accompanied by dynamic neuronal migration, connectivity establishment, and functional maturation (Maksimovic et al. 2022). This crucial period is characterized by an accelerated process of neurogenesis, gliogenesis, and synaptogenesis, which collectively contribute to the brain's high plasticity and vulnerability (Fan et al. 2021; Vanderhaeghen & Polleux 2023). At this developmental stage, the brain exhibits heightened sensitivity to disturbances arising from both environmental stimuli and pharmacological exposures.

An expanding corpus of evidence derived from both invertebrate and vertebrate models indicates that administration of general anesthetics during the BGS period can elicit extensive neurodevelopmental disruptions. These include the induction of neuronal apoptosis, inhibition of dendritic arborization, impairment of synaptogenesis, and disruption of synaptic plasticity, collectively contributing to persistent deficits in learning, memory, and long-term neurological function (Schaefer et al. 2019; Xu et al. 2018). It is now well-recognized that such adverse neurodevelopmental outcomes are mechanistically linked to the modulation of synaptic signaling by anesthetic agents. In particular, these drugs enhance inhibitory neurotransmission via GABA_A_Rs and/or suppress excitatory activity by antagonizing N-methyl-D-aspartate (NMDA) receptors—alterations that critically impair synaptic formation and stability during key developmental periods (Forcelli et al. 2011; Zhou et al. 2011). In clinical practice, GABA_A_R agonists are more commonly administered than NMDA receptor antagonists such as ketamine or nitrous oxide. Frequently utilized GABA_A_R-targeting anesthetics encompass inhalational agents—such as sevoflurane, isoflurane, and desflurane—as well as intravenous drugs including propofol, etomidate, and a range of benzodiazepines(BZDs, e.g., midazolam, diazepam, remimazolam) and barbiturates (e.g., thiopental, pentobarbital).

GABA_A_R agonists such as propofol disrupt a critical developmental process in which GABA transitions from depolarizing to inhibitory action. By enhancing inhibitory signals, these agents disturb the balance between excitatory and inhibitory signaling, thereby compromising neural network stability and causing long-term damage to developing brain networks. Consequently, these drugs have been included on the FDA's warning list due to their potential neurotoxic risks to children(Food & Administration 2017).

Neuroactive steroids (NAS), a subclass of endogenous steroid compounds, act as powerful allosteric modulators of GABA_A_Rs,s and play a pivotal role in modulating inhibitory neurotransmission within the central nervous system. While NAS agents such as CDNC, alphaxalone, and 3β-hydroxyandrostanes share sedative and anesthetic properties akin to propofol, they differ significantly in their neurotoxicity profiles. Notably, even at equivalent or supratherapeutic concentrations, these compounds do not elicit neuroapoptotic responses. In contrast, conventional anesthetics including isoflurane and propofol have been shown to induce widespread neuronal apoptosis when administered during critical windows of brain development. These neurotoxic effects are intimately linked to enduring impairments in synaptogenesis, reduced neural plasticity, and cognitive decline (Xu et al. 2018; Zhao et al. 2023). Such findings underscore the distinctive neurodevelopmental safety profile of NAS agents relative to traditional anesthetics like propofol (Atluri et al. 2018; Tesic et al. 2020). Furthermore, NAS agents demonstrate more pronounced neuroprotective properties, such as preventing neuronal apoptosis, protecting against neurodegenerative diseases, and exerting anti-neuroinflammatory effects (Diviccaro et al. 2022).

Given the divergent mechanisms of action and neurodevelopmental consequences observed between traditional intravenous anesthetics and emerging NAS, this review systematically examines the neurotoxic risks and neuroprotective potential of GABAergic intravenous anesthetics during early brain development. Particular attention is devoted to the reversibility and long-term consequences of developmental impairments resulting from anesthetic exposure. This discussion provides deeper insights into the molecular and cellular pathways implicated in anesthetic-induced neurotoxicity, while also exploring critical factors that modulate individual susceptibility to these adverse effects. Lastly, we evaluate emerging strategies aimed at preventing or attenuating anesthetic-related neurodevelopmental harm.

## Neurotoxic mechanisms of anesthetics targeting GABA_A_Rs

**Fig. 1:**
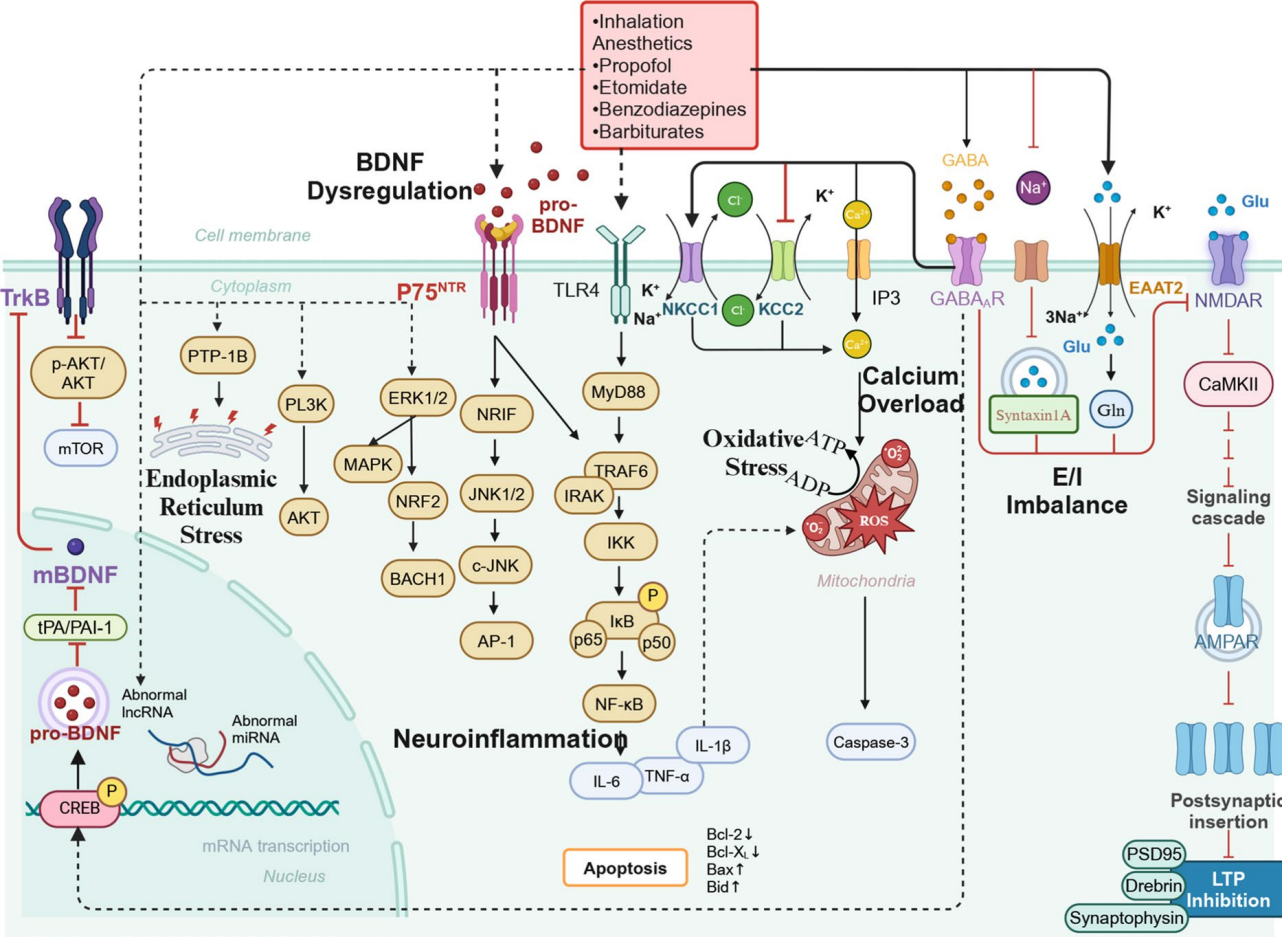
Mechanisms of developmental neurotoxicity induced by anesthetics targeting GABA_A_Rs. Anesthetic drugs target GABA_A_R in immature neurons, leading to developmental neurotoxicity. Black arrows represent the direct effects of anesthetic drugs on immature neurons, while red arrows indicate the subsequent effects triggered by the inhibition of relevant signaling pathways. The underlying mechanisms include: GA upregulating NKCC1 and downregulating KCC2, thereby enhancing GABA-mediated excitatory signaling, which leads to excessive calcium influx and calcium overload. This overload then triggers neuroinflammation and exacerbates oxidative stress. Through NMDAR-mediated excitatory/inhibitory imbalance (E/I imbalance), synaptic plasticity is disrupted, with a reduction in PSD95 and related proteins, as well as inhibition of LTP. Furthermore, GA affects the BDNF signaling pathway by enhancing BDNF/P75^NTR^, inhibiting BDNF/TrkB signaling, and causing BDNF dysregulation. Simultaneously, it interferes with abnormal ncRNA and PTP-1B signaling pathways, inducing endoplasmic reticulum stress, which ultimately results in neuronal damage

During key stages of neurodevelopment, the precisely coordinated activity of GABAergic and glutamatergic signaling cascades is essential for orchestrating neural stem cell proliferation, directing lineage-specific differentiation, and ensuring neuronal survival and maturation. General anesthetics, by profoundly modulating these excitatory and inhibitory neurotransmitter systems, have been demonstrated to disrupt the finely tuned synaptic equilibrium essential for normal brain development during these sensitive periods. This dysregulation is partially mediated by alterations in the NKCC1/KCC2 expression ratio, which governs intracellular chloride gradients and GABAergic polarity (see Section 'Disruption of Neurotransmitter Balance by Anesthetics'), as well as through direct interference with the functional integrity of GABA_A_Rs and NMDA receptors (Cabrera et al. 2020). In addition, anesthetic agents have been reported to modulate the expression patterns of BDNF, a key neurotrophin involved in synaptic regulation, thereby influencing neuron–glia crosstalk and initiating downstream neuroinflammatory responses (Wan et al. 2021). Moreover, anesthetic agents influence the expression of BDNF, thereby modulating the functional dynamics of both neurons and glial cells, and eliciting neuroinflammatory responses. This leads to calcium overload, enhanced oxidative stress, mitochondrial damage, endoplasmic reticulum stress, and alterations in non-coding RNAs (ncRNAs). These interacting mechanisms impair the proper assembly of neural circuits during development and may exert enduring detrimental effects on overall brain function (Fig. 1).

### Disruption of neurotransmitter balance by anesthetics

During early brain development, GABA is released prior to the functional maturation of glutamatergic synapses. In the neonatal cortex, high expression of the sodium–potassium–chloride cotransporter 1 (NKCC1) establishes a depolarizing chloride gradient, thereby rendering GABAergic signaling excitatory during this critical developmental window. As the brain matures, the expression of potassium–chloride cotransporter 2 (KCC2) progressively increases, replacing NKCC1 activity and resulting in a developmental transition in GABA function—from excitatory to inhibitory. This dynamic transition between excitatory and inhibitory signals ensures that neurons receive sufficient excitatory inputs during development while progressively establishing inhibitory neural networks (Oh et al. 2016; Watanabe & Fukuda 2015). GABA_A_R agonists, such as propofol and etomidate, may disrupt the excitation/inhibition (E/I) balance by increasing NKCC1 expression or inhibiting the maturation of KCC2 (Ju et al. 2017). This disruption leads to chloride extrusion and membrane depolarization, ultimately resulting in neuronal hyperexcitability and cognitive dysfunction (DiGruccio et al. 2015). In addition to modulating neurotransmitter systems, general anesthetics have been shown to activate voltage-gated calcium channels—particularly L-type channels—thereby elevating intracellular calcium concentrations. This abnormal calcium influx disrupts neuronal calcium homeostasis, a fundamental regulator of cell viability and intracellular signaling, and initiates a cascade of pathological events that ultimately lead to neurodegeneration. These observations underscore calcium dysregulation as a central pathogenic mechanism in anesthetic-induced neurotoxicity (Miao et al. 2022; Schaefer et al. 2019; Soyalp et al. 2022). BZDs like midazolam and diazepam enhance central GABAergic function by binding to GABA_A_Rs and modulating downstream signaling pathways, including those involving protein kinase G and synaptic marker interactions (Fredriksson et al. 2007; Jevtovic-Todorovic et al. 2003). For instance, midazolam requires higher doses to induce sedation in neonatal animals because, at elevated concentrations, the anesthetic's effect on KCC2 surpasses its impact on NKCC1, ultimately achieving sedation through neuronal hyperpolarization (Doi et al. 2021). However, excessive dosages increase the risk of neurotoxicity, particularly in infants, as anesthetic doses often exceed those used in adults, rendering them more susceptible to neuronal injury (Cattano et al. 2008; Huang et al. 2012; Sall et al. 2012). In contrast, remimazolam, with its lower equivalent dosage, short half-life, and minimal accumulation, results in less memory impairment. This is attributed to the reduced affinity of its metabolites for GABA_A_Rs, which is approximately 1/400 of remimazolam's affinity, thereby decreasing NMDAR overexpression and alleviating synaptic damage (Shi et al. 2024).

During intense synaptic activation, Glutamate (Glu) induces postsynaptic membrane depolarization by activating AMPA receptors, which relieves the magnesium block on NMDA receptors and allows Ca^2^⁺ to enter, triggering long-term potentiation (LTP). Subsequently, Postsynaptic density protein 95 (PSD95) functions as a pivotal structural scaffold at the synapse, anchoring NMDA and AMPA receptors at the postsynaptic membrane, facilitating AMPA receptor insertion, and enabling the induction of LTP and synaptic plasticity, thereby strengthening synaptic connectivity. Conversely, GABA_A_R activation has been shown to impair LTP induction—a fundamental cellular mechanism underpinning learning and memory—by attenuating Glu release at excitatory synapses. This inhibition of excitatory neurotransmission disrupts synaptic strengthening mechanisms essential for cognitive development. In vitro experiments indicate that propofol inhibits Glu release by suppressing presynaptic membrane depolarization, which reduces Na⁺ influx, while its effect on presynaptic Ca^2^⁺ influx and the reverse transport of transporters is not significant (Bademosi et al. 2018; Karunanithi et al. 2020). Evidence from animal models indicates that propofol, at concentrations consistent with clinical use, exerts direct modulatory effects on postsynaptic GABA_A_Rs, thereby enhancing inhibitory neurotransmission during critical stages of brain development (Li et al. 2020). At clinically relevant concentrations, both propofol and etomidate have been shown to interact with the vesicle docking protein syntaxin1 A, thereby restricting its lateral mobility and impairing Glu exocytosis from glutamatergic neurons in the basal forebrain of mice (Bademosi et al. 2018; Karunanithi et al. 2020). This molecular interaction diminishes neuronal excitability and contributes to functional impairment in cortical networks. Thiopental, another intravenous anesthetic, has been implicated in neonatal neurodegeneration by inhibiting Glu synthesis and promoting apoptotic signaling via upregulation of pro-apoptotic proteins (Naseri et al. 2017). Concurrently, gestational exposure to sevoflurane has been linked to sustained downregulation of key synaptic proteins—most notably PSD95 and synaptophysin—in the hippocampus of both fetal and postnatal offspring, suggesting long-lasting impairments in synaptic architecture and integrity (Zheng et al. 2013). This effect may involve modulation of the ubiquitin–proteasome pathway. Impaired PSD95 expression destabilizes postsynaptic AMPA receptor anchoring, enhancing receptor internalization through clathrin-mediated endocytosis. These alterations compromise hippocampal synaptic potentiation and are associated with memory deficits in rodent behavioral paradigms (Liao et al. 2021; Lu et al. 2017).

### Impact of anesthetics on BDNF

BDNF, a pivotal modulator of neurodevelopment, governs fundamental processes including neuronal differentiation, axonal and dendritic growth, and synaptic plasticity. Initially synthesized as the precursor protein proBDNF, BDNF is predominantly stored within synaptic vesicles and subsequently converted into its mature form (mBDNF) through proteolytic processing—a transformation essential for synaptic remodeling and the refinement of neural circuits during critical windows of brain development. Binding of proBDNF to its receptor p75^NTR^ activates the RhoA signaling cascade, resulting in axonal elongation and growth cone collapse (Head et al. 2009). During neurodevelopment, BDNF regulates the development of GABAergic, glutamatergic, and other synaptic types to ensure the precise establishment and stability of neural networks in the brain (Szymanski & Minichiello 2022).General anesthetics such as propofol significantly affect BDNF and its associated signaling pathways by modulating GABAergic and glutamatergic neurotransmission, thereby disrupting neuroplasticity (Cohen-Cory et al. 2010). Experimental studies have demonstrated that propofol attenuates the signaling cascade of BDNF and its receptor TrkB, thereby leading to reduced expression of PSD95 and impairing synaptic plasticity (Wan et al. 2021). Simultaneously, propofol activates the P75^NTR^/RhoA/ROCK pathway and GABA_A_Rs, increasing the ratio of proBDNF to mBDNF, disrupting the homeostasis of neurotrophic factor signaling, and exacerbating neuronal apoptosis, leading to neurological dysfunction (Kahraman et al. 2008). Furthermore, propofol significantly suppresses the expression of BDNF, BCL-2, and phosphorylated cAMP response element-binding protein (p-CREB) in hippocampal neurons, thereby intensifying its neurotoxic impact (Wei et al. 2016).

Within the context of early neural development, repeated exposure to sevoflurane disrupts the equilibrium between tissue plasminogen activator and its endogenous inhibitor PAI-1, thereby impairing the fibrinolytic cascade and compromising synaptic plasticity. This dysregulation subsequently hinders the activation of TrkB signaling and reduces the proteolytic processing of proBDNF into mBDNF (Dong et al. 2020). In parallel, sevoflurane engages the proBDNF–p75^NTR^ axis to trigger the JNK/c-JUN/AP-1 cascade (Bi et al. 2018), while simultaneously promoting neurodegenerative signaling via β-arrestin 1/2-mediated stimulation of metabotropic Glu receptors, leading to ERK1/2-MAPK activation and downstream NRF2/BACH1 transcriptional modulation (Wang et al. 2016; Yang et al. 2020a, b). Disruption of the BDNF–TrkB–PI3 K–Akt–mTOR signalling cascade in both cortical and hippocampal circuits is increasingly recognised as a key contributor to the neurotoxic effects observed following exposure to general anaesthetics (Wang & Wang 2019). In postnatal day 6 mice, exposure to sevoflurane induces pathological activation of GSK-3β, resulting in aberrant tau hyperphosphorylation, upregulation of IL-6 in the hippocampus, and diminished expression of the synaptic scaffolding protein PSD95. These molecular alterations converge to impair cognitive performance, suggesting a mechanistic link between anesthetic exposure, neuroinflammation, and synaptic dysfunction (Faraco et al. 2019; Tao et al. 2014).

Furthermore, Repeated exposure to BZDs, including remimazolam and midazolam, has been demonstrated to disrupt short-term cognitive processes such as learning and memory in juvenile rodent models. This cognitive impairment may be mechanistically attributed to a reduction in BDNF, upregulated Caspase-3 expression, diminished phosphorylation of PSD95, and suppression of LTP within the hippocampus, culminating in the exacerbation of hippocampal neuronal cell death (Shi et al. 2024).

### Effects of anesthetics on glial cells and inflammation

**Fig. 2:**
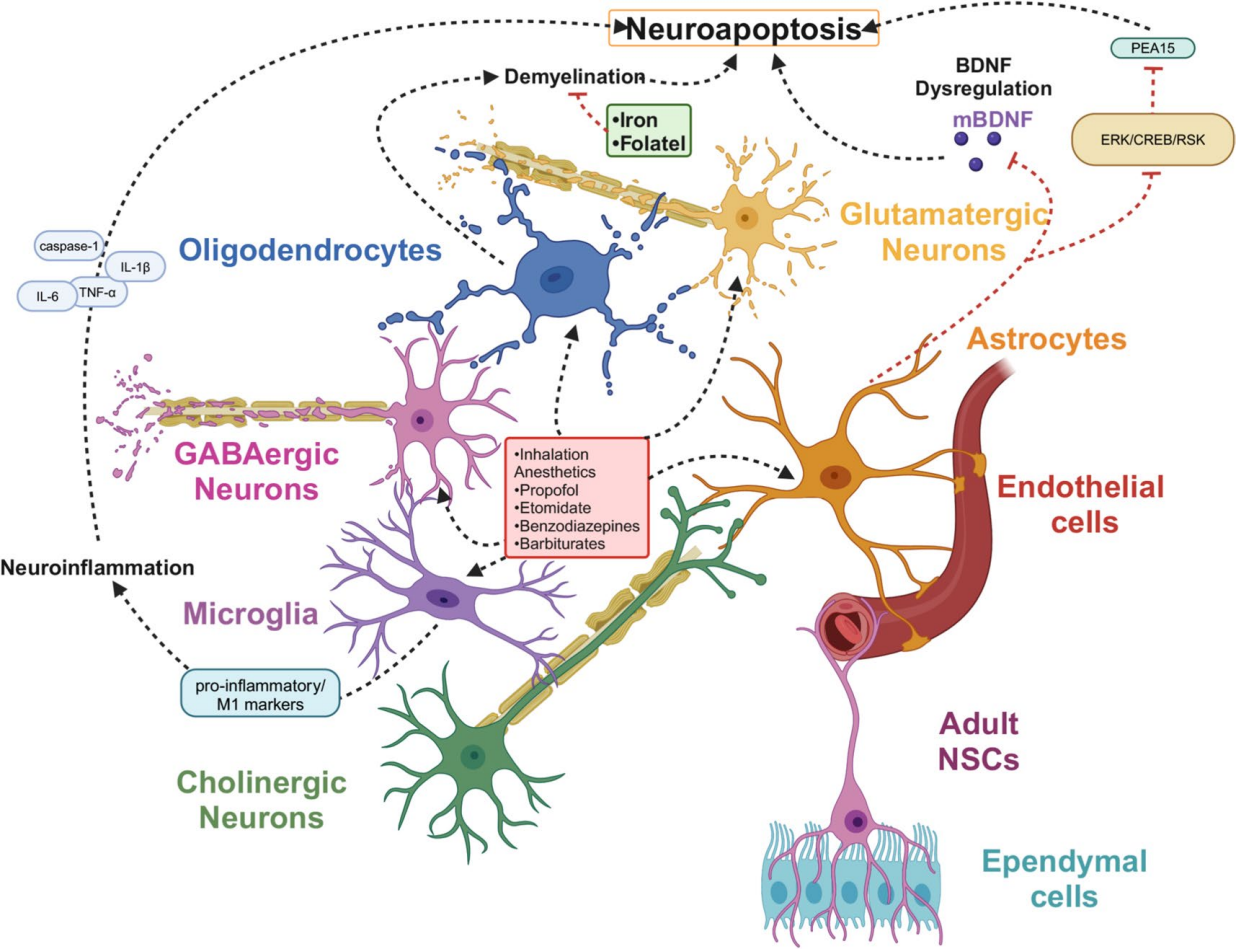
The effects of anesthetics targeting GABA_A_Rs on different neuronal populations. Anesthetic agents activate microglia, triggering the release of pro-inflammatory cytokines and M1 markers, which in turn exacerbate neuroinflammation. These drugs induce massive apoptosis in oligodendrocytes, leading to neuronal demyelination. Notably, iron and folate have been shown to mitigate this demyelination process. Furthermore, anesthetics interfere with astrocyte function via the ERK pathway, disrupting BDNF metabolism and ultimately leading to neuronal apoptosis. This cascade of events underscores the harmful effects of anesthetics on neurodevelopment, particularly in the context of inflammation-induced apoptosis in both neurons and glial cells

Anesthetic agents exert their effects not only on neurons but also extensively influence glial populations in the developing brain, with notable impacts on astrocytes, microglia, and oligodendrocytes. Amplified inflammatory activity within astrocytes and microglia is a defining feature of neuroinflammation, marked by glial cell activation or infiltration of immune cells that subsequently drive the upregulation of pro-inflammatory mediators (Mendiola & Cardona 2018). Oligodendrocytes further influence neurodevelopment through their role in myelination (Fig. 2).

Microglial activation is broadly acknowledged as a central driver of pro-inflammatory cytokine and chemokine production within the central nervous system. Persistent or excessive activation of these immune mediators is intimately linked to neuronal injury and is thought to underlie anaesthesia-related neuroinflammatory pathologies (Chen et al. 2018). Repeated exposure to sevoflurane during the neonatal stage has been demonstrated to markedly stimulate microglial activation, leading to heightened expression of pivotal neuroinflammatory mediators such as TNF-α, IL-6, and IL-1β (M. Yang et al. 2020a, b). Moreover, prenatal anaesthetic exposure via maternal administration disrupts fetal glial cell signaling, prominently increasing IL-6 production through extracellular signal-regulated kinase phosphorylation (Hirotsu et al. 2019). Concurrently, neonatal exposure to sevoflurane significantly upregulates sirtuin 2 in the hippocampus, promoting microglial polarization towards a pro-inflammatory M1 phenotype (Wu et al. 2020c). Beyond inhalational anesthetics, intravenous agents such as propofol and midazolam have also been associated with microglial activation, leading to an upsurge in the release of inflammatory mediators, notably IL-1β and TNF-α (Popić et al. 2015; Ramirez et al. 2016). These responses contribute to neuroinflammatory cascades and interfere with developmental neurogenesis (Milanovic et al. 2016). Exposure to propofol during early postnatal development significantly elevates the expression of pro-inflammatory markers—including caspase-1 and interleukin-1β—in both cortical and thalamic regions of postnatal day 7 rats (Milanovic et al. 2016). Importantly, microglial activation induced by sevoflurane has likewise been linked to the pathogenesis of various neurodegenerative conditions (Yeh et al. 2017).

Astrocytes are essential regulators of neural circuit formation and play a fundamental role in sustaining synaptic architecture and functionality throughout neurodevelopment.. Anesthetics may disrupt astrocyte density and function, thereby impairing BDNF secretion and indirectly affecting neuronal survival. For example, in high-density co-cultures of astrocytes, BDNF secretion effectively rescues propofol-induced neuronal death, whereas low-density astrocytes, which secrete insufficient BDNF, fail to provide the same protection (Liu et al. 2017a, b). These observations may offer mechanistic insight into the heightened susceptibility of the developing brain to anesthetic-induced neurotoxicity. Within the hippocampus, astrocytes enriched in phosphoprotein enriched in astrocytes 15 (PEA15) are prominently represented, with PEA15 expression shown to increase progressively as astrocytes undergo maturation. Mounting evidence indicates that propofol interferes with neurodevelopment by suppressing the expression of PEA15, thereby perturbing the ERK/CREB/RSK2 signaling axis. This disruption ultimately results in diminished neuronal proliferation alongside an increase in programmed cell deat (Xian et al. 2019).

Oligodendrocytes are central mediators of myelin sheath formation, facilitating efficient axonal conduction and supporting the maturation of neural circuits (Choi et al. 2019; Thomason et al. 2020). Myelination, a tightly regulated and temporally coordinated process occurring during early postnatal development, plays a pivotal role in the emergence of higher-order cognitive capacities and is intimately associated with the dynamic remodeling of both structural and functional neural circuitry (Deoni et al. 2018). Oligodendrocytes are not fully mature during the neonatal period, and anesthetic-induced inflammation may permanently damage myelination, hindering normal neurodevelopment. Studies have shown that anesthetic drugs have a far greater impact on oligodendrocyte apoptosis than on neurons, with apoptosis occurring at twice the rate in oligodendrocytes, representing 59% of all apoptotic cells (Schenning et al. 2017). Administration of propofol during vulnerable windows of early neurodevelopment markedly amplifies apoptotic activity in both neuronal and oligodendrocyte populations, concurrently triggering a substantial upregulation of pro-inflammatory cytokine expression. These molecular and cellular disruptions exhibit a strong association with enduring behavioural impairments manifesting in later stages of life (Milanovic et al. 2016; Ye et al. 2013). Moreover, gestational exposure to sevoflurane has been shown to impair myelination in offspring by inducing iron deficiency, thereby compromising myelin structure and reducing overall myelin content (Zuo et al. 2020). Similarly, exposure to high concentrations of sevoflurane during the early postnatal phase has been linked to impaired oligodendrocyte maturation and disruption of the normal trajectory of white matter myelination in the developing rat brain. These disturbances in oligodendrocyte lineage development contribute to central nervous system dysmyelination, a pathological process that may underlie long-term cognitive impairments (Wu et al. 2020b).

### Effects of anesthetics on calcium overload, oxidative stress, and endoplasmic reticulum stress

Appropriate Ca^2^⁺ levels are crucial for regulating oxidative phosphorylation in the mitochondrial matrix, thereby maintaining normal ATP production rates. Nevertheless, excessive calcium influx—particularly originating from the cytosol or the endoplasmic reticulum (ER)—can compromise mitochondrial respiratory chain function, thereby promoting the overproduction of reactive oxygen species (ROS). The resulting elevation in intracellular ROS levels ultimately culminates in oxidative DNA damage (Piao et al. 2020). Excessive generation and accumulation of ROS compromise the structural integrity of the mitochondrial membrane, disrupt cellular energy homeostasis by impairing ATP synthesis, and increase mitochondrial outer membrane permeability (MOMP), collectively triggering signaling cascades that culminate in cellular dysfunction (Calvo-Rodriguez & Bacskai 2021; Marchi et al. 2018).

General anesthetics have been shown to induce excessive activation of inositol 1,4,5-trisphosphate (IP₃) receptors, leading to a pronounced increase in cytosolic Ca^2^⁺ levels while simultaneously depleting calcium stores within the endoplasmic reticulum. This disruption in intracellular calcium homeostasis may represent a key mechanistic link underlying anesthesia-induced neurotoxicity during brain development (Yang & Wei 2017). For instance, the combination of propofol and midazolam significantly increases mitochondrial ROS levels, inhibits antioxidant enzyme activity, and impairs synaptic function (Boscolo et al. 2013). Propofol uncouples oxidative phosphorylation, disrupting mitochondrial function and increasing ROS production, thereby exacerbating neurotoxicity, which is difficult to mitigate through pretreatment (Shibuta et al. 2022). When midazolam is combined with nitrous oxide and isoflurane, ROS levels increase by approximately 30%, and antioxidant enzyme activity is notably reduced (Boscolo et al. 2013). Anesthetic compounds—particularly BZDs such as midazolam and diazepam—have been associated with detrimental neurodevelopmental consequences, primarily through the disruption of synaptic architecture, impairment of synaptic transmission, and induction of neuronal apoptosis (Lee et al. 2015).

Exposure to sevoflurane has been demonstrated to activate the PI3 K/Akt signaling cascade, thereby perturbing the balance between pro-survival and pro-apoptotic pathways and triggering apoptotic cell death. This dysregulation enhances MOMP, ultimately triggering intrinsic apoptotic pathways and contributing to long-term cognitive impairments observed during adolescence (Hu et al. 2019; Yu et al. 2020). In parallel, mounting evidence implicates ER stress as a key mediator of sevoflurane-induced neurotoxicity, primarily via the PERK–eIF2α–ATF4–CHOP signaling pathway (Liu et al. 2017a, b). In the developing brain, exposure to sevoflurane has also been shown to upregulate the expression of protein tyrosine phosphatase 1B within the ER, thereby initiating ER stress responses and promoting neurodegenerative alterations (Liu et al. 2019).

### Effects of anesthetics targeting GABA_A_Rs on ncRNAs

Emerging evidence highlights the pivotal influence of ncRNAs in orchestrating diverse neurobiological processes through intricate post-transcriptional regulatory mechanisms. General anesthetics modulate the expression profiles of microRNAs (miRNAs) and long non-coding RNAs (lncRNAs) in the developing brain, forming complex molecular regulatory networks that may perturb critical neural pathways. Such dysregulation is implicated in the induction of acute neuronal apoptosis and persistent memory deficits, potentially resulting in irreversible neurodevelopmental alterations (Jiang et al. 2021; Rodrigues et al. 2020; Shu et al. 2019).

With respect to apoptotic mechanisms, sevoflurane has been shown to upregulate the expression of caspase-3 and Bax, while concurrently downregulating BCL-2, BDNF, and nerve growth factor. These changes are accompanied by alterations in lncRNA expression and a reduction in hippocampal neuronal density, collectively leading to ultrastructural abnormalities and neuronal apoptosis (Hu et al. 2019). Moreover, the long non-coding RNA LRCF has been identified as a pivotal regulatory element in mediating isoflurane-induced apoptosis of oligodendrocytes. In neonatal mice, expression levels of LRCF are significantly elevated relative to those in adults. This upregulation promotes apoptotic signaling through activation of the HIF-1α/caspase-3 axis. Conversely, reduced LRCF expression appears to exert neuroprotective effects by recruiting the alternative HIF-1α/miR-138-5p/caspase-3 pathway. These expression-dependent regulatory divergences may account for the heightened susceptibility of the developing brain to anesthesia-induced neurotoxicity during the neonatal period. Consequently, high LRCF expression may serve as a high-risk biomarker for oligodendrocyte damage caused by anesthesia (Zeng et al. 2021). Exposure to sevoflurane has been demonstrated to influence synaptic plasticity by upregulating the expression of synaptophysin mRNA, a key presynaptic marker, via a mechanism dependent on N6-methyladenosine RNA modification. Paradoxically, this post-transcriptional modification contributes to a subsequent decline in synaptophysin protein abundance, which is associated with disrupted motor coordination and impaired cognitive performance in juvenile micee (Zhang et al. 2022a, b). Isoflurane anesthesia has been reported to downregulate the expression of microRNA-132, thereby reducing dendritic spine density in the hippocampus, a morphological alteration that contributes to impaired learning and memory performance in rodent models (Zhang et al. 2017).

## Neuroprotective potential of anesthetics

**Fig. 3:**
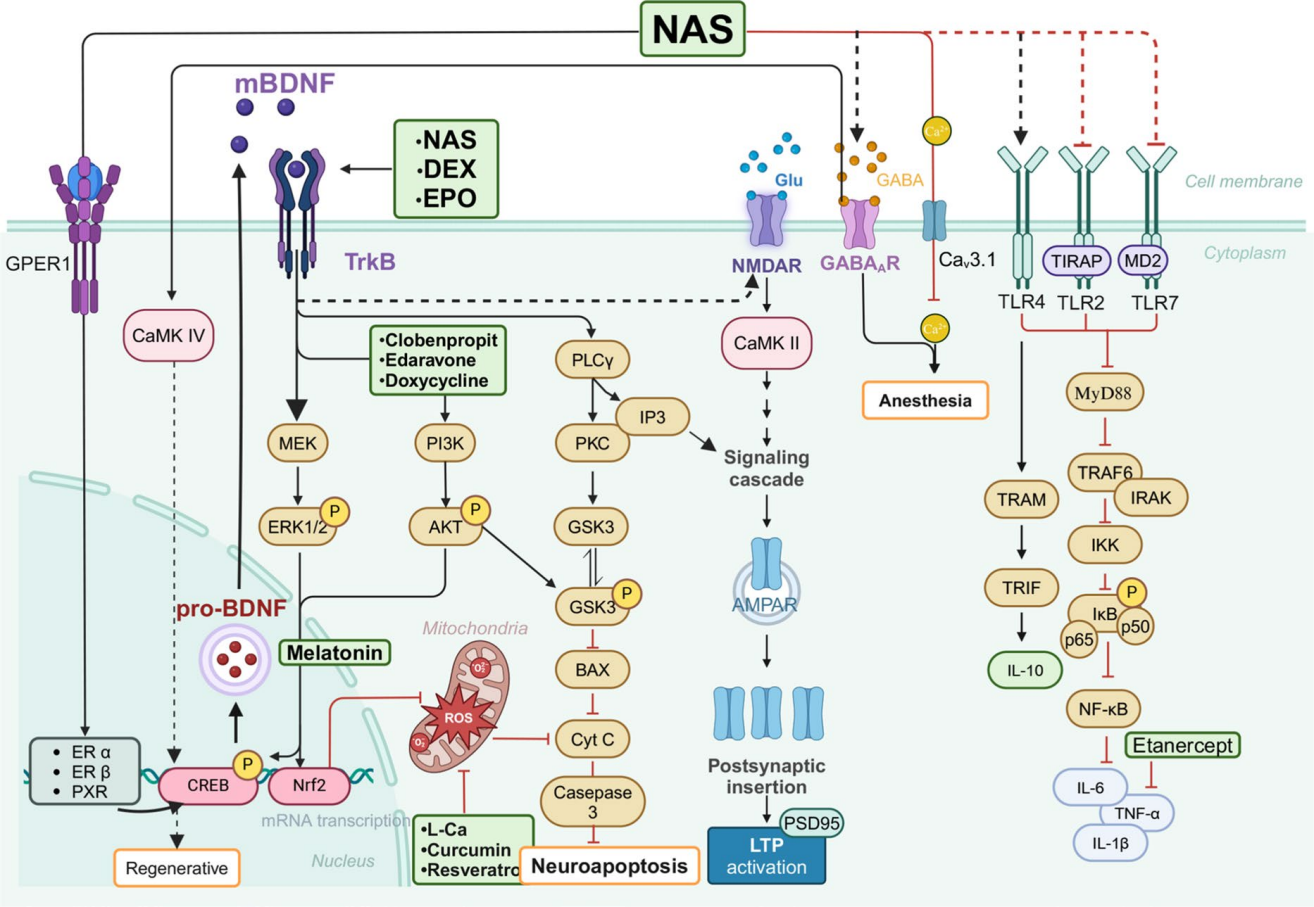
Neuroprotective mechanisms of NAS. NAS exerts neuroprotective effects through multiple pathways. The mechanisms are as follows: black arrows indicate the direct effects of anesthetics on immature neurons, while red arrows represent subsequent effects induced by the inhibition of related signaling pathways. NAS interacts with TLR2, TLR4, and TLR7 and their adaptor proteins to reduce the production of inflammatory factors, thereby alleviating neuronal inflammation. NAS also regulates the synthesis of mBDNF through receptors such as GPER1, ERα/β, and PXR. mBDNF activates MEK/ERK, PI3 K/AKT, and PLCγ/PKC signaling pathways through TrkA/B receptors, inhibiting ROS production, alleviating mitochondrial dysfunction, reducing neuronal apoptosis, enhancing neurotransmitter release, and activating NMDAR to promote LTP formation, thus increasing synaptic plasticity. Additionally, NAS may exert anesthetic effects by modulating GABA_A_Rs and Ca^2^⁺ while enhancing neuronal regeneration to further protect the nervous system. NAS, DEX, and EPO reduce neuroinflammation and exhibit anti-apoptotic and neuroprotective effects through the BDNF/TrkB pathway, while Clobenpropit, Edaravone, and Doxycycline act through the BDNF/PI3 K pathway. Melatonin increases BDNF production through Nrf2, while L-Ca, Curcumin, and Resveratrol reduce oxidative stress, and Etanercept reduces the production of inflammatory factors

NAS, as sedatives and anesthetics, demonstrate significant neuroprotective potential (Tateiwa & Evers 2024). By modulating both inhibitory and excitatory neurotransmitter systems, NAS maintain the stability of neural networks. Additionally, NAS promote the release of BDNF, a molecule essential for supporting neuronal growth, viability, and synaptic plasticity. Furthermore, NAS regulate inflammation and oxidative stress, alleviating neuroinflammation and reducing oxidative damage, thereby effectively protecting neuronal cells from injury. These multiple mechanisms collectively highlight the potential of NAS agents as neurodevelopmental protective compounds (Fig. 3).

### Modulation of the inhibitory neurotransmitter system

While most NAS serve as potent allosteric modulators of GABA_A_R, enhancing or inhibiting GABA-induced currents via subtype-specific binding—such as potentiation by 3α-hydroxylated neurosteroids and suppression by 3β-sulfated derivatives—emerging evidence indicates a divergence in neurotoxic potential among GABA_A_R-activating agents (Chen et al. 2019; Laverty et al. 2017), Notably, compounds like propofol, CDNC24, and alphaxalone all activate GABA_A_Rs, yet only propofol has been consistently associated with significant neurotoxicity during brain development, whereas NAS such as alphaxalone exhibit negligible or no overt neurotoxic effects (Tesic et al. 2020). Further investigations revealed that NAS compounds, such as alphaxalone and 3β-OH, do not induce neuronal apoptosis, regardless of their activation of GABA_A_Rs. The neuroprotective properties of these compounds may be partially attributed to their ability to inhibit T-type calcium channels, particularly Cav3.1, thereby diminishing presynaptic GABA release (Tesic et al. 2020; Timic Stamenic et al. 2021). Intriguingly, the mechanism of action of 3β-OH appears to operate independently of classical modulation of GABAergic or NMDA receptor pathways (Atluri et al. 2018; Timic Stamenic et al. 2021). NAS enhances neuronal regeneration. For instance, allopregnanolone binds to the transmembrane domain of the GABA_A_R complex, activating the receptor and increasing intracellular Ca^2^⁺ concentrations. This calcium influx subsequently activates CaMK IV, which phosphorylates and activates the transcription factor CREB1. Once activated, CREB1 enhances the transcription of genes involved in cell cycle regulation, thereby promoting mitotic activity in neural stem cells and oligodendrocyte precursor cells (Li et al. 2018a, b, c). Moreover, NAS demonstrates significant potential for selective regulation of different GABA_A_R subtypes. These compounds can modulate receptor tonic currents by enhancing GABA_A_R-mediated current activity. At moderate concentrations, NAS compounds inhibit GABA_A_R function, whereas at lower and higher concentrations, they enhance GABA regulation. This suggests that NAS compounds exert a complex bidirectional effect on GABA_A_Rs within different concentration ranges, potentially providing a new therapeutic strategy for neuroprotection by modulating neural activity(Fig. 3).

### Modulation of the excitatory neurotransmitter system

NAS, such as 3β-OH, reduces presynaptic AMPA receptor-mediated excitatory synaptic currents but does not affect NMDAR-mediated excitatory currents, thus modulating the glutamatergic system (Atluri et al. 2018). Gonadal steroids, such as estradiol and 17β-estradiol, potentiate NMDAR-mediated synaptic transmission and facilitate the release of Glu from primary afferent terminals, thereby increasing dendritic spine density in dorsal horn neurons of the spinal cord (Zhang et al. 2012). Estradiol not only enhances the binding of NMDAR agonists and competitive antagonists (Kow et al. 2005) but may also exert a dual action by enhancing cognitive function, promoting synaptic transmission while offering neuroprotective effects (Nilsen et al. 2002). The enantiomer of 17β-estradiol exhibits neuroprotective properties against Glu-induced toxicity in cultured neuronal cells, implying that its protective effects may be mediated through pathways independent of classical nuclear estrogen receptors (Green et al. 2001). These studies suggest that NAS drugs exhibit neuroprotective potential by modulating NMDARs and the Glu transport system within a specific concentration range(Fig. 3).

### Promotion of BDNF release

Many studies have indicated that NAS can bind to and regulate various proteins involved in cell protection, including estrogen receptors α/β (ERα/β), the orphan nuclear receptor pregnane-X receptor (PXR), and the G protein-coupled estrogen receptor 1 (GPER1), thereby modulating BDNF levels (Briz & Baudry 2014; Jakab et al. 2001; Serrao & Goodchild 2022). For example, the enantiomer of 17β-estradiol regulates BDNF levels through an ERβ-mediated mechanism (Jakab et al. 2001). This modulatory action not only contributes to improved learning and memory performance but also serves a pivotal role in mitigating cognitive impairments induced by anesthesia (Taylor et al. 2017). Additionally, neurosteroid drugs such as alphaxalone activate PXR, further promoting the secretion of BDNF and exerting neuroprotective effects (Frye et al. 2014; Serrao & Goodchild 2022). Clinical randomized controlled trials (RCTs) have demonstrated that patients treated with alphaxalone during hip replacement surgery show better cognitive performance postoperatively, with significantly higher plasma levels of BDNF than patients treated with propofol and sevoflurane (Serrao & Goodchild 2022). These findings suggest that NAS, by modulating BDNF and associated pathways, exhibits significant neuroprotective effects, particularly in the recovery of cognitive function following anesthesia and surgery.

### Regulation of inflammation and oxidative stress

Neurosteroids, such as pregnenolone, exert anti-inflammatory effects through multiple signaling pathways, including Toll-like receptor signaling, the TRAM-TRIF pathway, BDNF, and CX3 CL1 signaling pathways. NAS suppress the initiation of pro-inflammatory signaling cascades by disrupting the binding of Toll-like receptors—specifically TLR2, TLR4, and TLR7—to their adaptor proteins such as MyD88, MD2, and TIRAP, thereby reducing downstream synthesis of pro-inflammatory mediators (Balan et al. 2019; Balan et al. 2023a, b; Balan et al. 2023a, b). In addition to its established role in suppressing pro-inflammatory signaling, NAS activate the TLR4–TRAM–TRIF pathway, leading to the upregulation of key anti-inflammatory mediators such as IL-10 and BDNF, thereby contributing to neuroprotection (Aurelian & Balan 2019). NAS also promote the expression of the anti-inflammatory chemokine CX3 CL1, which is essential for maintaining the equilibrium between pro- and anti-inflammatory responses within the central nervous system, ultimately alleviating neuroinflammation. Furthermore, NAS have been shown to attenuate microglial pro-inflammatory activation by engaging GABAARs and modulating the BDNF–TrkA/TrkB signaling cascade, while concurrently regulating the transcription of pro-inflammatory genes (Alexaki et al. 2018; Wu et al. 2020a, b, c). Beyond anti-inflammatory action, NAS support neuronal repair and regeneration through diverse neuroprotective mechanisms, offering potential therapeutic benefits in the context of neurodegenerative conditions and traumatic brain injury. Collectively, these findings underscore the central role of inflammation and oxidative stress in anesthetic-induced neurotoxicity. Importantly, NAS modulate antioxidant and anti-inflammatory processes not only through GABAergic pathways but also via alternative non-GABAergic mechanisms, highlighting their promise in mitigating the adverse neurodevelopmental effects associated with anesthetic exposure.

## Susceptibility factors for anesthetic-induced neurotoxicity

### Dose-, time-, and frequency-dependent effects of anesthetics

Anesthetic-induced neurotoxicity demonstrates marked dependence on dose, exposure duration, and administration frequency. Evidence from both cellular and animal models indicates that anesthetic agents, such as propofol, may facilitate the differentiation of neural progenitor cells into neurons when administered at very low concentrations (Qiao et al. 2017). However, at subclinical or medium-to-high doses, these anesthetics inhibit neuronal proliferation, increase pro-inflammatory factor levels, and trigger widespread neuronal apoptosis, with the extent of apoptosis gradually increasing with higher doses (Chen et al. 2022a, b; Han et al. 2015). Anesthetic neurotoxicity also demonstrates clear time dependence, with neuronal apoptosis worsening as the exposure duration increases (Huang et al. 2012; Zhao et al. 2016). In rodent models, brief exposure to propofol (2 or 4 h) induced marked activation of caspase-3, a key executor of apoptosis. Notably, a more prolonged exposure of 6 h elicited not only a pronounced increase in Caspase-3 but also elevated calpain activity within the cerebral cortex and thalamus. These molecular alterations were concomitant with the emergence of apoptotic neuronal profiles, indicating that extended anesthetic exposure may potentiate neurodegenerative processes in specific brain regions (Milanovic et al. 2010). Prolonged exposure to sevoflurane impairs the maturation of neural circuits by inhibiting synaptogenesis and destabilizing dendritic spine development. This anesthetic disruption is further characterized by structural synaptic alterations, including increased expression of synaptic vesicle-related proteins, reduced density of apical dendritic spines, and ultrastructural disorganization within hippocampal synapses, which collectively contribute to cognitive deficits observed in juvenile rats (Yu et al. 2020). Moreover, a study comparing single-dose versus seven-dose administrations of propofol with the same total dose showed that rats exposed multiple times exhibited significant impairments in spatial learning and hippocampal function, suggesting that frequent exposures may have a more severe impact on neurological function than a single cumulative dose (Gao et al. 2014). Recurrent administration of sevoflurane during critical periods of cortical development has been implicated in long-term synaptic dysfunction, accompanied by structural synaptic anomalies and persistent cognitive deficits manifesting in adulthood (Li et al. 2019a, b, c; Zhou et al. 2019).

### Synergistic effects of combined anesthesia

Emerging evidence indicates that the combined administration of anesthetic agents may markedly potentiate neuronal apoptosis and disrupt hippocampal synaptic integrity, thereby impairing learning and memory through synergistic neurotoxic effects (Jevtovic-Todorovic et al. 2003). For instance, during critical windows of brain maturation, the combined administration of ketamine with thiopental or propofol significantly amplifies neuronal cell death in neonatal animals and results in enduring functional impairments in adulthood, whereas the administration of these agents individually tends to produce only modest neurodevelopmental effects (Fredriksson et al. 2007). The combined use of anesthetics such as isoflurane, midazolam, and nitric oxide in a"triple cocktail"may reduce synaptic density (Lunardi et al. 2010), destabilize mitochondria (Sanchez et al. 2011), and increase autophagic activity (Fredriksson et al. 2007), which could contribute to neurodegenerative changes. Combined exposure to midazolam and nitrous oxide perturbs the homeostasis of mitochondrial dynamics by dysregulating key modulators such as Mfn-2 and Drp-1. This imbalance leads to pronounced mitochondrial morphological alterations, compromised bioenergetic function, and subsequent impairments in neuronal integrity and performance (Boscolo et al. 2013).

### Region-specific sensitivity to anesthetics during critical periods of brain development

**Fig. 4:**
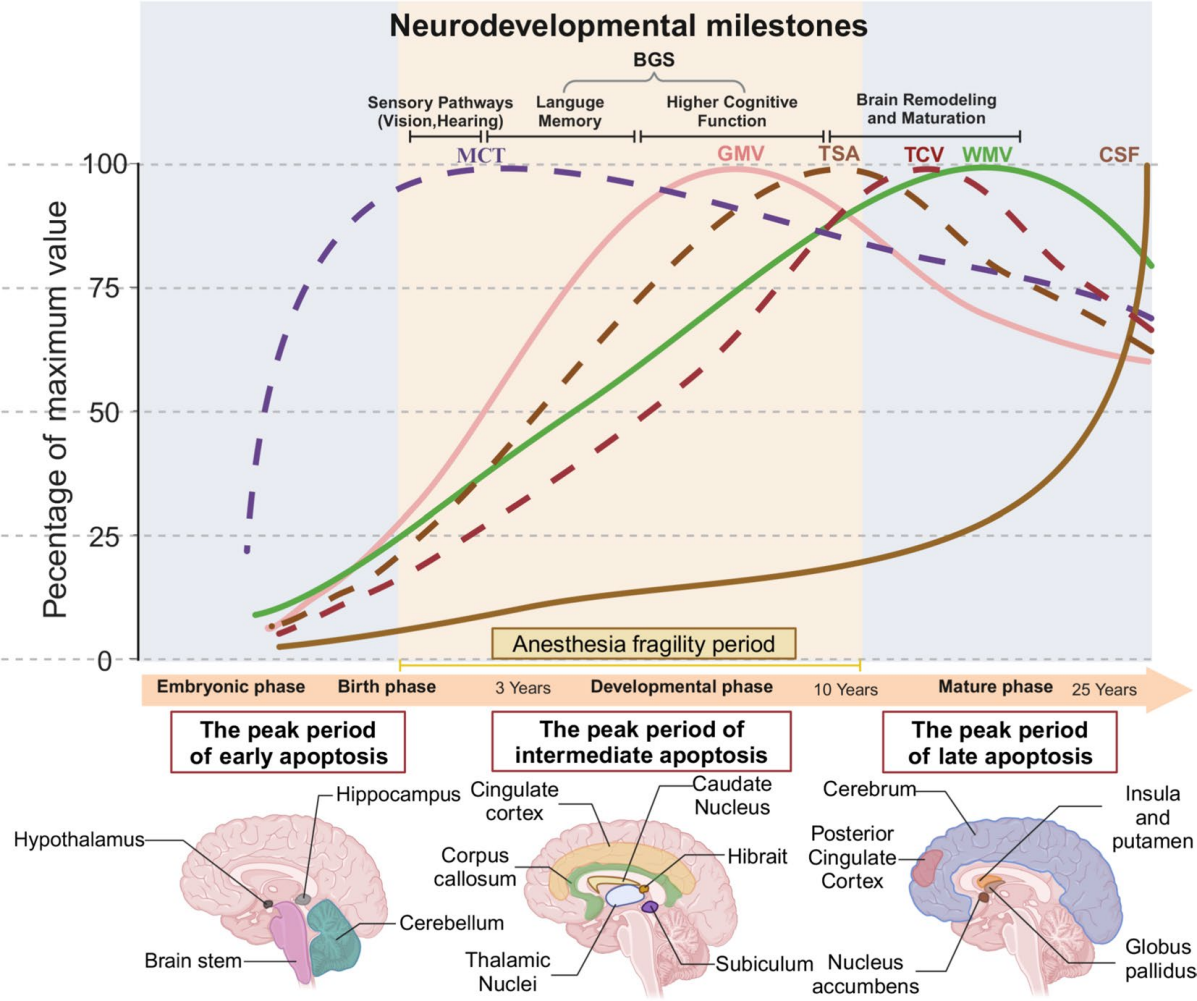
The impact of anesthetic agents on different brain regions during development. Neurodevelopmental trajectories based on global pediatric MRI data are presented, with the y-axis representing the percentage of maximum volume attained by each brain region during development, and the x-axis representing age. The yellow shaded region highlights the period of anesthesia vulnerability, in comparison to the critical peak developmental periods for various brain functions.The impact of anesthetic drugs on different brain regions is shown, with highlighted areas indicating regions particularly vulnerable to anesthesia-induced damage. BGS:brain growth spurt;GMV:total cortical grey matter volume;WMV:total white matter volume;CSF:total ventricular cerebrospinal fluid volume (ventricles or cerebrospinal fluid);MCT:mean cortical thickness;TSA:total surface area; TCV:total cerebrum volume

During BGS, exposure to anesthetic drugs can lead to widespread neuronal apoptosis and result in persistent behavioral deficits (Gentry et al. 2013). The temporal window of the BGS varies significantly across mammalian species. In rodents, this critical phase of neurodevelopment unfolds within the first two weeks following birth (Semple et al. 2013); whereas in rhesus macaques, it spans from gestational day 115 to approximately postnatal day 60 (Brambrink et al. 2012); and in humans, the growth spurts occur at varying times across different brain regions, with most regions peaking in the second year after birth and typically reaching their maximum between 3 and 10 years of age (Andropoulos & Greene 2017). Neuronal apoptosis induced by anesthetic drugs exhibits significant regional specificity at different time points during the BGS. In rodents, postnatal day 0 is the peak period of early apoptosis, primarily occurring in neurons of the hypothalamus and certain thalamic nuclei; day 3 marks the peak of intermediate apoptosis, affecting regions such as the Subiculum, Hibrait, caudate nucleus, and thalamic nuclei; day 7 represents the peak of late apoptosis, predominantly affecting cortical neurons (Maloney et al. 2019). Similarly, in a comparable model using rhesus monkeys, prenatal exposure to propofol predominantly induces apoptosis in neurons and oligodendrocytes within the cerebellum, hypothalamus, and additional posterior and anterior brain regions, whereas exposure during the neonatal period primarily affects the cerebral cortex (Creeley et al. 2013) (Fig. 4).

### Vulnerable populations for anesthetic-induced neurotoxicity

Neonates—particularly those born prematurely, with low birth weight, reduced gestational age, or complex congenital anomalies—constitute a highly vulnerable population, exhibiting an elevated susceptibility to anesthesia-related neurodevelopmental impairment. Neuroimaging studies have revealed that preterm infants frequently display structural abnormalities in both gray and white matter following neonatal surgery. These alterations, detectable by MRI, have been strongly correlated with subsequent cognitive deficits and delays in neurodevelopmental milestones (Filan et al. 2012; Stolwijk et al. 2017). Weiss et al. noted that neonatal brains are highly sensitive to ischemic injury, placing these groups at a higher risk for neurodevelopmental disorders during the perioperative period. Specifically, during anesthesia, changes in hemodynamics, respiration, and metabolism may have a greater impact on neurodevelopment than the anesthetic drugs themselves (Weiss et al. 2016). Furthermore, factors such as increased surgical frequency, prolonged hospital stays, and prolonged respiratory support further exacerbate the risk of neurodevelopmental disorders. These impairments are not only related to the direct effects of surgery and anesthesia, but may also be closely associated with hemodynamic instability, improper respiratory control, and metabolic imbalance during anesthesia, compounded by surgical stress and primary diseases (Stolwijk et al. 2016).

## Potential strategies to prevent and reverse neurotoxicity

### Combination of newer anesthetic agents

Dexmedetomidine (DEX), a highly selective α₂-adrenergic receptor agonist, has emerged as a potent neuroprotective agent against isoflurane-induced neurotoxicity, primarily due to its robust anti-apoptotic properties. Mechanistically, DEX exerts its neuroprotective effects through engagement with several converging molecular cascades. These include the DNMT3a–miR-377-5p–Arc axis and the ERK1/2–CREB–BDNF signaling pathway(Xiao et al. 2018), along with activation of the PI3 K–Akt cascade. Concurrently, it inhibits pro-apoptotic processes by downregulating the GSK-3β–CRMP2 and CDK5–CRMP2 pathways (Chen et al. 2022a, b). Pretreatment with DEX has also been shown to counteract the isoflurane-mediated repression of neurotrophic mediators such as BDNF mRNA, phosphorylated ERK1/2, CREB, and BDNF protein, thereby preserving synaptic homeostasis under anesthetic-induced stress(Tu et al. 2019). Additional studies have confirmed that the suppression of the GSK-3β/CRMP2 and CDK5/CRMP2 pathways by DEX contributes to reduced neuronal apoptosis and cognitive decline in neonatal models (Li et al. 2019a, b, c). Moreover, the neuroprotection afforded by DEX extends beyond apoptosis, encompassing antioxidative and anti-inflammatory actions that mitigate sevoflurane-induced cognitive impairments (Wang et al. 2015). In neonatal rats, DEX may also confer protection via modulation of glutamatergic transmission (Wang et al. 2019). Activation of α₂-adrenergic receptors by DEX and related agonists like clonidine has additionally been found to attenuate tau hyperphosphorylation and its associated cognitive sequelae in neonatal mice exposed to sevoflurane (Sun et al. 2021). Notably, the neuroprotective efficacy of DEX in anesthetic regimens appears dose-dependent: at low concentrations, co-administration with sevoflurane enhances neuroprotection; however, higher doses paradoxically increase apoptosis and mortality, potentially due to reduced α₂ receptor selectivity and inadvertent α₁ receptor activation(Liu et al. 2016; Perez-Zoghbi et al. 2020) (Perez-Zoghbi et al. 2017) (Fig. 3).

Novel BZDs such as remimazolam, when combined with sevoflurane, significantly reduce the incidence of postoperative delirium, suggesting a neuroprotective potential that may be linked to reduced overall anesthetic exposure(Yang et al. 2022). The combined use of anesthetics might also protect the brain by balancing excitatory and inhibitory pathways. For example, midazolam could facilitate the reuptake of excess Glu via EAAT2 and enhance GABA_A_R activity, inhibiting the release of Glu transporters, thereby effectively alleviating excitotoxicity caused by the upregulation of NMDAR NR1 subunits after ketamine withdrawal(Li et al. 2018a, b, c; Slikker et al. 2007). Moreover, NAS such as 17β-estradiol may enhance AKT phosphorylation, increase p-GSK-3β levels, and stimulate BDNF release(Li et al. 2019a, b, c; Yang et al. 2022), effectively mitigating the apoptosis induced by combinations of triple cocktails(Lu et al. 2006) or ketamine(Li et al. 2014). Alphaxalone has been reported to mitigate the neurotoxic effects elicited by isoflurane exposure in neonatal rats, highlighting its potential as a neuroprotective agent during early brain development (Zhao et al. 2023).

### Neuroprotective agents

#### Anti-apoptotic agents

Neuronal apoptosis is a major mechanism of developmental neurotoxicity induced by GABA_A_R receptor agonists. Inhibiting apoptotic pathways may serve as a potential intervention. The histamine H₃ receptor antagonist Clobenpropit has been shown to activate the PI3 K/Akt signaling cascade, thereby mitigating isoflurane-induced apoptotic injury in hippocampal neurons in vitro (He et al. 2018). Complementarily, the TNF-α inhibitor Etanercept significantly reduced neuronal apoptosis in the developing rat brain following isoflurane exposure (Chen et al. 2016), suggesting a role for inflammatory modulation in anesthetic-induced neurotoxicity. In a separate study, Straiko and colleagues demonstrated that the co-administration of lithium with either ketamine or propofol did not significantly alter caspase-3 activation levels compared to control conditions, indicating a limited effect of lithium in modulating anesthetic-induced apoptosis via this pathway (Straiko et al. 2009). Moreover, erythropoietin (EPO) can partially reverse the decrease in BDNF and BCL-2 levels caused by general anesthetics, thereby mitigating neuronal damage(Pellegrini et al. 2014).

#### Anti-inflammatory agents

Neuroinflammation has emerged as a critical mediator of anesthetic-induced neurotoxicity, making it a compelling target for therapeutic intervention. Recent findings indicate that edaravone confers neuroprotection against isoflurane-induced neuronal injury by attenuating inflammatory and apoptotic responses via activation of the mBDNF–TrkB–PI3 K signaling axis(Yang et al. 2021). These beneficial effects have been validated across both in vitro neuronal culture systems and in vivo preclinical models, highlighting the therapeutic promise of neurotrophic pathway modulation in mitigating anesthesia-related neural damage. In parallel, doxycycline—a semi-synthetic tetracycline antibiotic—has demonstrated potent neuroprotective capabilities beyond its antimicrobial function. Through engagement of the PI3 K/Akt pathway, doxycycline has been shown to exert significant anti-inflammatory and anti-apoptotic effects, supporting its candidacy as a therapeutic agent for inflammation-related neurodevelopmental pathologies(Möller et al. 2016).

#### Anti-oxidative stress neuroprotective agents

Recent advances in neuropharmacology underscore the neuroprotective potential of natural antioxidants against anesthesia-induced neurotoxicity. Melatonin, for instance, has been shown to activate the PKCα/Nrf2 signaling cascade, upregulate the anti-apoptotic protein BCL-xL, suppress cytochrome c release, and inhibit mitochondrial-dependent apoptotic pathways. Through these mechanisms, melatonin markedly reduces neuronal apoptosis triggered by combined exposure to midazolam, nitrous oxide, and isoflurane, suggesting a protective role in anesthetic neurotoxicity(Li et al. 2018a, b, c; Yon et al. 2006). Similarly, curcumin (Ji et al. 2015) and resveratrol(Tang et al. 2021), both known for their antioxidant properties, have been demonstrated to attenuate sevoflurane-induced oxidative stress by neutralizing ROS and suppressing associated inflammatory responses. Acetyl-L-carnitine (L-Ca), a mitochondria-targeted antioxidant, has been identified as neuroprotective in aging and neurodegenerative conditions(Liu et al. 2014; Robinson et al. 2019; Yan et al. 2014). These findings highlight the promise of L-Ca as a potential adjunct in anesthetic protocols, particularly those involving agents with known oxidative liabilities.

#### Nutritional supplements for neuroprotection

Calcium homeostasis disruption is a key mechanism underlying anesthetic-induced neurotoxicity, involving mitochondrial dysfunction as well as damage to astrocytes and neurons. Emerging evidence indicates that stabilizing intracellular calcium dynamics may represent a promising strategy to counteract the neurodevelopmental toxicity associated with sevoflurane exposure(Zhu et al. 2024). Iron deficiency has been recognized as a pivotal determinant of impaired myelination within the central nervous system, exerting its most detrimental effects during the fetal period and early stages of postnatal development. Disruptions in iron homeostasis during these sensitive periods may compromise oligodendrocyte function and hinder the proper formation of myelin sheaths(Ward et al. 2014). Sevoflurane exposure has been implicated in disrupting myelin development during early brain maturation, potentially through mechanisms involving oligodendrocyte apoptosis, as well as deficiencies in iron and folate metabolism. Supplementing with iron and folate and inhibiting OL apoptosis could be effective preventive measures, although further research is needed.

These findings suggest that strategies aimed at preventing apoptosis and inflammation, reducing oxidative stress, and restoring calcium homeostasis and nutrient balance may offer promising interventions against sevoflurane-induced neurotoxicity (Fig. 3).

## Concluding remarks and future directions

During the BGS, dynamic balance between the GABA/Glu systems plays a crucial role in neural development, with the transition from NKCC1 to KCC2 being critical in regulating the E/I signal balance. Anesthetics, particularly intravenous agents targeting GABA_A_Rs, may disrupt this transition, thereby destabilizing neural networks(Cabrera et al. 2020). Additional mechanisms include the imbalance of the BDNF signaling pathway, oxidative stress responses, and inflammatory reactions, all of which exacerbate neurotoxicity. Compared with traditional anesthetics, newer agents such as NAS(Tesic et al. 2020; Timic Stamenic et al. 2021), remimazolam(Shi et al. 2024), and DEX(Wei et al. 2016) exhibit milder mechanisms of action, maintaining sufficient anesthetic effects while minimizing interference with the nervous system. However, our understanding remains limited regarding how novel NAS and similar compounds may modulate T-type calcium channels and selectively bind to specific sites on GABA_A_R, thereby facilitating endogenous regenerative processes in the brain and mitigating the neurotoxic effects of anesthetic agents. Further investigation is essential to elucidate their potential in alleviating neurotoxicity. Concurrently, significant strides are being made in the development of neuroprotective pharmacotherapies. Agents such as melatonin (Li et al. 2018a, b, c), EPO(Pellegrini et al. 2014), and resveratrol (Tang et al. 2021), —all of which exhibit potent antioxidant, anti-inflammatory, and neuroprotective properties—have been investigated as promising candidates for mitigating anesthetic-induced neurotoxicity (Fig. 3).Accordingly, the development of next-generation anesthetic agents should prioritise strategies that minimise neurotoxicity, enhance intrinsic neuroprotective mechanisms, and facilitate post-injury repair and functional recovery—thereby ensuring both the safety and therapeutic efficacy of anaesthesia in paediatric and other high-risk populations.

Since the 2016 advisory issued by the FDA regarding the potential neurotoxic effects of anesthetic agents in pediatric populations, there has been a marked increase in clinical investigations evaluating the neurodevelopmental consequences of anesthetic exposure in children. Findings from key studies such as the PANDA study (Sun et al. 2016) and GAS trial (Davidson et al. 2016; McCann et al. 2019) have shown that a single, brief exposure—typically under one hour—to general anesthesia in children younger than five years of age is not associated with significant long-term neurodevelopmental deficits. However, the MASK study provided evidence that multiple exposures prior to age three may be linked to slower cognitive processing, impaired fine motor coordination, and increased risk of behavioral and learning difficulties. Supporting this, recent data suggest that general anesthesia administered to infants under one year of age is associated with an elevated risk of developmental delays, with risk escalating as the number of surgical procedures increases (Kobayashi et al. 2020). While the majority of clinical studies suggest that isolated anesthetic exposure is relatively safe, the literature remains inconclusive due to ethical constraints, methodological limitations, and perioperative confounders. Notably, the combined neurotoxic effects of multiple anesthetic agents and their potential protective mechanisms remain underexplored. Future research should adopt an integrative approach, combining mechanistic molecular studies, behavioral analyses in animal models, and rigorously designed clinical trials. Prospective randomized controlled trials with long-term neurodevelopmental follow-up are essential. Moreover, the establishment of standardized dosing frameworks and exposure durations, alongside anesthesia protocols tailored to regional brain vulnerability across developmental stages, will be critical to advancing this field.

Clinical investigations into the long-term cognitive and behavioral consequences of anesthetic exposure in children employ heterogeneous methodologies. However, a significant proportion of these studies rely predominantly on subjective outcome assessments (Maloney et al. 2019), with limited incorporation of objective neuroimaging techniques or biomarker analyses, thereby constraining a comprehensive evaluation of anesthetic-induced neurodevelopmental effects (Bethlehem et al. 2022; Zhang et al. 2022a, b). Notably, quantitative ultrasound imaging has revealed increased apoptotic activity in the developing brains of non-human primates following sevoflurane exposure, highlighting the potential neurotoxic impact of this agent during vulnerable periods of brain maturation (Rosado-Mendez et al. 2019). Complementary neuroimaging studies suggest that pediatric exposure to inhalational anesthetics such as isoflurane and sevoflurane is associated with cognitive impairments and disruptions in white matter integrity (Banerjee et al. 2019; Bethlehem et al. 2022). Moreover, prospective investigations report that as many as 75% of infants undergoing non-cardiac surgery for congenital anomalies exhibit mild to moderate neuroimaging abnormalities postoperatively (Mongerson et al. 2019; Moran et al. 2019). To delineate the precise neurodevelopmental impact of anesthetic agents, future studies should adopt an integrative approach—combining MRI and diffusion tensor imaging (DTI) with high-resolution ultrasound and biomarker profiling (e.g., inflammatory cytokines, BDNF)—in conjunction with longitudinal assessments of cognitive and behavioral function across defined developmental windows. This will help establish preventive or reversal strategies, such as the use of antioxidants, calcium channel blockers, or agents that promote myelination, or through environmental stimulation and functional training to restore the function of impaired neural networks.

## Conclusion

With the widespread clinical application of intravenous anesthetics targeting GABA_A_Rs, their potential impact on the developing nervous system has increasingly become a focal point of academic attention. Although extensive research has elucidated the neurodevelopmental impact of GABA_A_R–targeting agents via modulation of both GABA_A_Rs and NMDA receptors, the specific influence of anesthetic drugs on synaptogenesis remains a complex and multilayered phenomenon. Deciphering the long-term consequences of such agents, alongside the identification of potential neuroprotective strategies and the refinement of their clinical applicability, continues to pose substantial challenges for future investigations.

## Supplementary Information

Below is the link to the electronic supplementary material.

Supplementary file1 (PDF 8395 KB)

## Data Availability

No datasets were generated or analysed during the current study.

## Declarations

### Sources and data illustration strategy

To illustrate the mechanisms of anesthetic-induced neurotoxicity, all pathway diagrams and synaptic models presented in this review were created using BioRender and manually curated from previously published peer-reviewed literature. No new experimental or clinical data were generated or analyzed for this work.
