# Supplementary material for: From neurotoxicity to neuroprotection: Rethinking GABAAR-targeting anesthetics
Source: Cell Biol Toxicol. 2025 Jun 14;41(1):104. doi: 10.1007/s10565-025-10057-z (PMC12167312; doi:10.1007/s10565-025-10057-z)
Supplement: Supplementary file 1 — Supplementary file1 (PDF 8395 KB) [file 10565_2025_10057_MOESM1_ESM.pdf]

20250528222701112523091301041766

WORD COUNT	14905	TIME SUBMITTED	28-MAY-2025 10:27PM
		PAPER ID	116471972

From Neurotoxicity to Neuroprotection: Rethinking $GABA_A$R-Targeting Anesthetics

Yubao Li[1,2], Hongliang Yang[1,2], Lu Liu[2,3], Lulu Jiang[2], Peilin Xie[2], Xiaoling Wang[2,3], Xuhui Cong[2], Ruilou Zhu[2], Zhongyuan Lu[2,3], Mingyang Sun[2*], Jiaqiang Zhang[2,4*]

[1]Xinxiang Medical University, Xinxiang, Henan, China.

[2]Department of Anesthesiology and Perioperative Medicine, Zhengzhou University People's Hospital and Henan provincial People's Hospital, Zhengzhou, Henan, China.

[3]Academy of Medical Science, Zhengzhou University, Zhengzhou, Henan, China.

[4]Institute of Electrophysiology, Henan Academy of Innovations in Medical Science, Zhengzhou, China

[*] **Correspondence author**

Jiaqiang Zhang and Mingyang Sun

Department of Anesthesiology and Perioperative Medicine,

Zhengzhou University People's Hospital and Henan provincial People's Hospital,

Zhengzhou, P.R. China

Email to zhangjiq@zzu.edu.cn(Jiaqiang Zhang) and also email to znxyfs@zzu.edu.cn (Mingyang Sun)

Telephone and fax numbers: +86 139-3712-1360, +86 5928 8887 777.

ABSTRACT

The brain growth spurt (BGS) represents a pivotal window in neurodevelopment, defined by rapid neurogenesis, heightened synaptogenesis, and the dynamic establishment of neural networks. During this phase, heightened brain plasticity significantly enhances learning and memory abilities, while also increasing the brain's susceptibility to disruptions. Anesthetics, particularly those targeting γ-aminobutyric acid type A receptors ($GABA_ARs$), interfere with GABAergic and glutamatergic systems, disrupt brain-derived neurotrophic factor (BDNF) signaling, and exacerbate neurotoxic effects. These agents activate glial cells, induce inflammation, and contribute to oxidative stress, while also disrupting calcium homeostasis and triggering endoplasmic reticulum stress. Furthermore, anesthetics alter the expression of non-coding RNAs, which affects gene regulation and long-term memory formation. The extent of neurotoxic effects is contingent upon a constellation of factors, including the timing, dosage, and frequency of anesthetic exposure, as well as individual susceptibility. Notably, perioperative administration of anesthetic agents has been implicated in long-term cognitive dysfunction, thereby emphasizing the critical importance of precisely modulated dosing regimens and temporally optimized delivery strategies to mitigate potential neurodevelopmental risks. In contrast, neuroactive steroids demonstrate promising neuroprotective potential by modulating $GABA_AR$ activity, enhancing BDNF release, and regulating oxidative stress and inflammation. New strategies for preventing and reversing anesthetic-induced neurotoxicity could include novel anesthetic combinations, anti-apoptotic agents, antioxidants, or nutritional supplements. These findings underscore the complex and multifactorial effects of anesthetic agents on the developing brain and emphasize the urgent need to establish and refine anesthetic strategies that safeguard neural integrity during vulnerable windows of neurodevelopment.

Keywords Anesthetics Targeting GABAARs; Neurodevelopment; Neurotoxicity; Neuroprotective; Neuroactive Steroids

1. Introduction

A growing body of evidence derived from both animal models and clinical research suggests that the postnatal BGS represents a critical period in neurodevelopment, during which the central nervous system displays exceptional plasticity and heightened susceptibility to external perturbations. During this critical developmental window, neuronal circuits are sculpted through the rapid generation and remodeling of trillions of synapses, accompanied by dynamic neuronal migration, connectivity establishment, and functional maturation(Maksimovic et al., 2022). This crucial period is characterized by an accelerated process of neurogenesis, gliogenesis, and synaptogenesis, which collectively contribute to the brain's high plasticity and vulnerability(Fan et al., 2021; Vanderhaeghen & Polleux, 2023). At this developmental stage, the brain exhibits heightened sensitivity to disturbances arising from both environmental stimuli and pharmacological exposures.

An expanding corpus of evidence derived from both invertebrate and vertebrate models indicates that administration of general anesthetics during the BGS period can elicit extensive neurodevelopmental disruptions. These include the induction of neuronal apoptosis, inhibition of dendritic arborization, impairment of synaptogenesis, and disruption of synaptic plasticity, collectively contributing to persistent deficits in learning, memory, and long-term neurological function (Schaefer et al., 2019; Xu et al., 2018). It is now well-recognized that such adverse neurodevelopmental outcomes are mechanistically linked to the modulation of synaptic signaling by anesthetic agents. In particular, these drugs enhance inhibitory neurotransmission via $GABA_A$Rs and/or suppress excitatory activity by antagonizing N-methyl-D-aspartate (NMDA) receptors—alterations that critically impair synaptic formation and stability during key developmental periods (Forcelli et al., 2011; Zhou et al., 2011). In clinical practice, $GABA_A$R agonists are more commonly administered than NMDA receptor antagonists such as ketamine or nitrous oxide. Frequently utilized $GABA_A$R-targeting anesthetics encompass inhalational agents—such as sevoflurane, isoflurane and desflurane—as well as intravenous drugs including propofol, etomidate, and a range of benzodiazepines (e.g., midazolam, diazepam, remimazolam) and barbiturates (e.g., thiopental, pentobarbital).

$GABA_A$R agonists such as propofol disrupt a critical developmental process in which GABA transitions from depolarizing to inhibitory action. By enhancing inhibitory signals, these agents disturb the balance between excitatory and inhibitory signaling, thereby compromising neural network stability and causing long-term damage to developing brain networks. Consequently, these drugs have been included on the FDA's warning list due to their potential neurotoxic risks to children(Food & Administration, 2017).

Neuroactive steroids (NAS), a subclass of endogenous steroid compounds, act as powerful allosteric modulators of $GABA_A$Rs,s and play a pivotal role in modulating inhibitory neurotransmission within the central nervous system. While NAS agents such as CDNC, alphaxalone, and 3β-hydroxyandrostanes share sedative and anesthetic properties akin to propofol, they differ significantly in their neurotoxicity profiles. Notably, even at equivalent or supratherapeutic concentrations, these compounds do not elicit neuroapoptotic responses. In contrast, conventional anesthetics including isoflurane and propofol have

been shown to induce widespread neuronal apoptosis when administered during critical windows of brain development. These neurotoxic effects are intimately linked to enduring impairments in synaptogenesis, reduced neural plasticity, and cognitive decline (Xu et al., 2018; Zhao et al., 2023). Such findings underscore the distinctive neurodevelopmental safety profile of NAS agents relative to traditional anesthetics like propofol(Atluri et al., 2018; Tesic et al., 2020). Furthermore, NAS agents demonstrate more pronounced neuroprotective properties, such as preventing neuronal apoptosis, protecting against neurodegenerative diseases, and exerting anti-neuroinflammatory effects(Diviccaro et al., 2022).

Given the divergent mechanisms of action and neurodevelopmental consequences observed between traditional intravenous anesthetics and emerging NAS, this review systematically examines the neurotoxic risks and neuroprotective potential of GABAergic intravenous anesthetics during early brain development. Particular attention is devoted to the reversibility and long-term consequences of developmental impairments resulting from anesthetic exposure. This discussion provides deeper insights into the molecular and cellular pathways implicated in anesthetic-induced neurotoxicity, while also exploring critical factors that modulate individual susceptibility to these adverse effects. Lastly, we evaluate emerging strategies aimed at preventing or attenuating anesthetic-related neurodevelopmental harm.

2. Neurotoxic Mechanisms of Anesthetics Targeting GABA$_A$ Rs

During key stages of neurodevelopment, the precisely coordinated activity of GABAergic and glutamatergic signaling cascades is essential for orchestrating neural stem cell proliferation, directing lineage-specific differentiation, and ensuring neuronal survival and maturation. General anesthetics, by profoundly modulating these excitatory and inhibitory neurotransmitter systems, have been demonstrated to disrupt the finely tuned synaptic equilibrium essential for normal brain development during these sensitive periods. This dysregulation is partially mediated by alterations in the NKCC1/KCC2 expression ratio, which governs intracellular chloride gradients and GABAergic polarity (see section 2.1), as well as through direct interference with the functional integrity of GABA$_A$Rs and NMDA receptors(Cabrera et al., 2020). In addition, anesthetic agents have been reported to modulate the expression patterns of BDNF, a key neurotrophin involved in synaptic regulation, thereby influencing neuron–glia crosstalk and initiating downstream neuroinflammatory responses(Wan et al., 2021). Moreover, anesthetic agents influence the expression of brain-derived neurotrophic factor (BDNF), thereby modulating the functional dynamics of both neurons and glial cells, and eliciting neuroinflammatory responses. This leads to calcium overload, enhanced oxidative stress, mitochondrial damage, endoplasmic reticulum stress, and alterations in non-coding RNAs (ncRNAs). These interacting mechanisms impair the proper assembly of neural circuits during development and may exert enduring detrimental effects on overall brain function(Fig. 1).

2.1 Disruption of Neurotransmitter Balance by Anesthetics

During early brain development, GABA is released prior to the functional maturation of glutamatergic synapses. In the neonatal cortex, high expression of the sodium–potassium–chloride cotransporter 1 (NKCC1) establishes a depolarizing chloride gradient, thereby rendering GABAergic

signaling excitatory during this critical developmental window. As the brain matures, the expression of potassium–chloride cotransporter 2 (KCC2) progressively increases, replacing NKCC1 activity and resulting in a developmental transition in GABA function—from excitatory to inhibitory. This dynamic transition between excitatory and inhibitory signals ensures that neurons receive sufficient excitatory inputs during development while progressively establishing inhibitory neural networks(Oh et al., 2016; Watanabe & Fukuda, 2015).$GABA_AR$ agonists, such as propofol and etomidate, may disrupt the excitation/inhibition (E/I) balance by increasing NKCC1 expression or inhibiting the maturation of KCC2(Ju et al., 2017). This disruption leads to chloride extrusion and membrane depolarization, ultimately resulting in neuronal hyperexcitability and cognitive dysfunction(DiGruccio et al., 2015). In addition to modulating neurotransmitter systems, general anesthetics have been shown to activate voltage-gated calcium channels—particularly L-type channels—thereby elevating intracellular calcium concentrations. This abnormal calcium influx disrupts neuronal calcium homeostasis, a fundamental regulator of cell viability and intracellular signaling, and initiates a cascade of pathological events that ultimately lead to neurodegeneration. These observations underscore calcium dysregulation as a central pathogenic mechanism in anesthetic-induced neurotoxicity(Miao et al., 2022; Schaefer et al., 2019; Soyalp et al., 2022). BZDs like midazolam and diazepam enhance central GABAergic function by binding to $GABA_ARs$ and modulating downstream signaling pathways, including those involving protein kinase G and synaptic marker interactions(Fredriksson et al., 2007; Jevtovic-Todorovic et al., 2003).. For instance, midazolam requires higher doses to induce sedation in neonatal animals because, at elevated concentrations, the anesthetic's effect on KCC2 surpasses its impact on NKCC1, ultimately achieving sedation through neuronal hyperpolarization(Doi et al., 2021). However, excessive dosages increase the risk of neurotoxicity, particularly in infants, as anesthetic doses often exceed those used in adults, rendering them more susceptible to neuronal injury. In contrast, remimazolam, with its lower equivalent dosage, short half-life, and minimal accumulation, results in less memory impairment. This is attributed to the reduced affinity of its metabolites for $GABA_ARs$, which is approximately 1/400 of remimazolam's affinity, thereby decreasing NMDAR overexpression and alleviating synaptic damage(Shi et al., 2024).

During intense synaptic activation, Glutamate (GLU) induces postsynaptic membrane depolarization by activating AMPA receptors, which relieves the magnesium block on NMDA receptors and allows calcium ions to enter, triggering long-term potentiation (LTP). Subsequently, Postsynaptic density protein 95 (PSD95) functions as a pivotal structural scaffold at the synapse, anchoring NMDA and AMPA receptors at the postsynaptic membrane, facilitating AMPA receptor insertion, and enabling the induction of long-term potentiation (LTP) and synaptic plasticity, thereby strengthening synaptic connectivity. Conversely, $GABA_AR$ activation has been shown to impair LTP induction—a fundamental cellular mechanism underpinning learning and memory—by attenuating GLU release at excitatory synapses. This inhibition of excitatory neurotransmission disrupts synaptic strengthening mechanisms essential for cognitive development. In vitro experiments indicate that propofol inhibits GLU release by suppressing presynaptic membrane depolarization, which reduces Na^+ influx, while its effect on presynaptic Ca^{2+} influx and the reverse transport of transporters is not significant(Bademosi et al., 2018; Karunanithi et al., 2020). Evidence from animal models indicates that propofol, at concentrations consistent with clinical use, exerts direct modulatory effects on postsynaptic $GABA_ARs$, thereby enhancing inhibitory neurotransmission during critical stages of brain development(Li et al., 2020). At clinically relevant concentrations, both propofol and etomidate have been shown to interact with the

vesicle docking protein syntaxin1A, thereby restricting its lateral mobility and impairing glutamate (GLU) exocytosis from glutamatergic neurons in the basal forebrain of mice (Bademosi et al., 2018; Karunanithi et al., 2020). This molecular interaction diminishes neuronal excitability and contributes to functional impairment in cortical networks. Thiopental, another intravenous anesthetic, has been implicated in neonatal neurodegeneration by inhibiting GLU synthesis and promoting apoptotic signaling via upregulation of pro-apoptotic proteins (Naseri et al., 2017). Concurrently, gestational exposure to sevoflurane has been linked to sustained downregulation of key synaptic proteins—most notably PSD95 and synaptophysin—in the hippocampus of both fetal and postnatal offspring, suggesting long-lasting impairments in synaptic architecture and integrity (Zheng et al., 2013). This effect may involve modulation of the ubiquitin–proteasome pathway. Impaired PSD95 expression destabilizes postsynaptic AMPA receptor anchoring, enhancing receptor internalization through clathrin-mediated endocytosis. These alterations compromise hippocampal synaptic potentiation and are associated with memory deficits in rodent behavioral paradigms (Liao et al., 2021; Lu et al., 2017).

2.2 Impact of Anesthetics on BDNF

BDNF, a pivotal modulator of neurodevelopment, governs fundamental processes including neuronal differentiation, axonal and dendritic growth, and synaptic plasticity. Initially synthesized as the precursor protein proBDNF, BDNF is predominantly stored within synaptic vesicles and subsequently converted into its mature form (mBDNF) through proteolytic processing—a transformation essential for synaptic remodeling and the refinement of neural circuits during critical windows of brain development. Binding of proBDNF to its receptor $p75^{NTR}$ activates the RhoA signaling cascade, resulting in axonal elongation and growth cone collapse(Head et al., 2009). During neurodevelopment, BDNF regulates the development of GABAergic, glutamatergic, and other synaptic types to ensure the precise establishment and stability of neural networks in the brain(Szymanski & Minichiello, 2022).

General anesthetics such as propofol significantly affect BDNF and its associated signaling pathways by modulating GABAergic and glutamatergic neurotransmission, thereby disrupting neuroplasticity(Cohen-Cory et al., 2010). Experimental studies have demonstrated that propofol attenuates the signaling cascade of BDNF and its receptor TrkB, thereby leading to reduced expression of PSD95 and impairing synaptic plasticity (Wan et al., 2021). Simultaneously, propofol activates the $P75^{NTR}$/RhoA/ROCK pathway and $GABA_A$Rs, increasing the ratio of proBDNF to mBDNF, disrupting the homeostasis of neurotrophic factor signaling, and exacerbating neuronal apoptosis, leading to neurological dysfunction(Kahraman et al., 2008). Furthermore, propofol significantly suppresses the expression of BDNF, B-cell lymphoma 2 (BCL-2), and phosphorylated cAMP response element-binding protein (p-CREB) in hippocampal neurons, thereby intensifying its neurotoxic impact(Wei et al., 2016).

Within the context of early neural development, repeated exposure to sevoflurane disrupts the equilibrium between tissue plasminogen activator and its endogenous inhibitor PAI-1, thereby impairing the fibrinolytic cascade and compromising synaptic plasticity. This dysregulation subsequently hinders

the activation of TrkB signaling and reduces the proteolytic processing of proBDNF into mBDNF(Dong et al., 2020). In parallel, sevoflurane engages the proBDNF–p75 neurotrophin receptor ($p75^{NTR}$) axis to trigger the JNK/c-JUN/AP-1 cascade (Bi et al., 2018), while simultaneously promoting neurodegenerative signaling via β-arrestin 1/2-mediated stimulation of metabotropic GLU receptors, leading to ERK1/2-MAPK activation and downstream NRF2/BACH1 transcriptional modulation (Wang et al., 2016; F. Yang et al., 2020). Disruption of the BDNF–TrkB–PI3K–Akt–mTOR signalling cascade in both cortical and hippocampal circuits is increasingly recognised as a key contributor to the neurotoxic effects observed following exposure to general anaesthetics(Wang & Wang, 2019). In postnatal day 6 (P6) mice, exposure to sevoflurane induces pathological activation of GSK-3β, resulting in aberrant tau hyperphosphorylation, upregulation of IL-6 in the hippocampus, and diminished expression of the synaptic scaffolding protein PSD95. These molecular alterations converge to impair cognitive performance, suggesting a mechanistic link between anesthetic exposure, neuroinflammation, and synaptic dysfunction(Faraco et al., 2019; Tao et al., 2014).

Furthermore, Repeated exposure to benzodiazepines, including remimazolam and midazolam, has been demonstrated to disrupt short-term cognitive processes such as learning and memory in juvenile rodent models. This cognitive impairment may be mechanistically attributed to a reduction in BDNF, upregulated caspase-3 expression, diminished phosphorylation of PSD95, and suppression of LTP within the hippocampus, culminating in the exacerbation of hippocampal neuronal cell death.(Shi et al., 2024).

2.3 Effects of Anesthetics on Glial Cells and Inflammation

Anesthetic agents exert their effects not only on neurons but also extensively influence glial populations in the developing brain, with notable impacts on astrocytes, microglia, and oligodendrocytes. Amplified inflammatory activity within astrocytes and microglia is a defining feature of neuroinflammation, marked by glial cell activation or infiltration of immune cells that subsequently drive the upregulation of pro-inflammatory mediators(Mendiola & Cardona, 2018). Oligodendrocytes further influence neurodevelopment through their role in myelination(Fig. 3).

Microglial activation is broadly acknowledged as a central driver of pro-inflammatory cytokine and chemokine production within the central nervous system. Persistent or excessive activation of these immune mediators is intimately linked to neuronal injury and is thought to underlie anaesthesia-related neuroinflammatory pathologies (Chen et al., 2018). Repeated exposure to sevoflurane during the neonatal stage has been demonstrated to markedly stimulate microglial activation, leading to heightened expression of pivotal neuroinflammatory mediators such as TNF-α, IL-6, and IL-1β (M. Yang et al., 2020). Moreover, prenatal anaesthetic exposure via maternal administration disrupts fetal glial cell signaling, prominently increasing IL-6 production through extracellular signal-regulated kinase (ERK) phosphorylation (Hirotsu et al., 2019). Concurrently, neonatal exposure to sevoflurane significantly upregulates sirtuin 2 (SIRT2) in the hippocampus, promoting microglial polarization towards a pro-inflammatory M1 phenotype (Z. Wu, Y. Zhang, et al., 2020). Beyond inhalational anesthetics, intravenous agents such as propofol and midazolam have also been associated with microglial activation, leading to an upsurge in the release of inflammatory mediators, notably IL-1β and TNF-α (Popić et al., 2015; Ramirez et al., 2016). These responses contribute to neuroinflammatory cascades and interfere

with developmental neurogenesis (Milanovic et al., 2016). Exposure to propofol during early postnatal development significantly elevates the expression of pro-inflammatory markers—including caspase-1 and interleukin-1β—in both cortical and thalamic regions of postnatal day 7 (PND7) rats(Milanovic et al., 2016). Importantly, microglial activation induced by sevoflurane has likewise been linked to the pathogenesis of various neurodegenerative conditions(Yeh et al., 2017).

Astrocytes are essential regulators of neural circuit formation and play a fundamental role in sustaining synaptic architecture and functionality throughout neurodevelopment. Anesthetics may disrupt astrocyte density and function, thereby impairing BDNF secretion and indirectly affecting neuronal survival. For example, in high-density co-cultures of astrocytes, BDNF secretion effectively rescues propofol-induced neuronal death, whereas low-density astrocytes, which secrete insufficient BDNF, fail to provide the same protection(Y. Liu et al., 2017). These observations may offer mechanistic insight into the heightened susceptibility of the developing brain to anesthetic-induced neurotoxicity. Within the hippocampus, astrocytes enriched in phosphoprotein enriched in astrocytes 15 (PEA15) are prominently represented, with PEA15 expression shown to increase progressively as astrocytes undergo maturation... Mounting evidence indicates that propofol interferes with neurodevelopment by suppressing the expression of PEA15, thereby perturbing the ERK/CREB/RSK2 signaling axis. This disruption ultimately results in diminished neuronal proliferation alongside an increase in programmed cell death(Xian et al., 2019).

Oligodendrocytes are central mediators of myelin sheath formation, facilitating efficient axonal conduction and supporting the maturation of neural circuits (Choi et al., 2019; Thomason et al., 2020). Myelination, a tightly regulated and temporally coordinated process occurring during early postnatal development, plays a pivotal role in the emergence of higher-order cognitive capacities and is intimately associated with the dynamic remodeling of both structural and functional neural circuitry(Deoni et al., 2018). Oligodendrocytes are not fully mature during the neonatal period, and anesthetic-induced inflammation may permanently damage myelination, hindering normal neurodevelopment. Studies have shown that anesthetic drugs have a far greater impact on oligodendrocyte apoptosis than on neurons, with apoptosis occurring at twice the rate in oligodendrocytes, representing 59% of all apoptotic cells(Schenning et al., 2017). Administration of propofol during vulnerable windows of early neurodevelopment markedly amplifies apoptotic activity in both neuronal and oligodendrocyte populations, concurrently triggering a substantial upregulation of pro-inflammatory cytokine expression. These molecular and cellular disruptions exhibit a strong association with enduring behavioural impairments manifesting in later stages of life (Milanovic et al., 2016; Ye et al., 2013). Moreover, gestational exposure to sevoflurane has been shown to impair myelination in offspring by inducing iron deficiency, thereby compromising myelin structure and reducing overall myelin content (Zuo et al., 2020). Similarly, exposure to high concentrations of sevoflurane during the early postnatal phase has been linked to impaired oligodendrocyte maturation and disruption of the normal trajectory of white matter myelination in the developing rat brain. These disturbances in oligodendrocyte lineage development contribute to central nervous system dysmyelination, a pathological process that may underlie long-term cognitive impairments (Z. Wu, H. Xue, et al., 2020).

2.4 Effects of Anesthetics on Calcium Overload, Oxidative Stress, and Endoplasmic Reticulum Stress

Appropriate calcium ion (Ca^{2+}) levels are crucial for regulating oxidative phosphorylation in the mitochondrial matrix, thereby maintaining normal ATP production rates. Nevertheless, excessive calcium influx—particularly originating from the cytosol or the endoplasmic reticulum (ER)—can compromise mitochondrial respiratory chain function, thereby promoting the overproduction of reactive oxygen species (ROS). The resulting elevation in intracellular ROS levels ultimately culminates in oxidative DNA damage(Piao et al., 2020). Excessive generation and accumulation of ROS compromise the structural integrity of the mitochondrial membrane, disrupt cellular energy homeostasis by impairing ATP synthesis, and increase mitochondrial outer membrane permeability (MOMP), collectively triggering signaling cascades that culminate in cellular dysfunction(Calvo-Rodriguez & Bacskai, 2021; Marchi et al., 2018).

General anesthetics have been shown to induce excessive activation of inositol 1,4,5-trisphosphate (IP_3) receptors, leading to a pronounced increase in cytosolic Ca^{2+} levels while simultaneously depleting calcium stores within the endoplasmic reticulum. This disruption in intracellular calcium homeostasis may represent a key mechanistic link underlying anesthesia-induced neurotoxicity during brain development(Yang & Wei, 2017). For instance, the combination of propofol and midazolam significantly increases mitochondrial ROS levels, inhibits antioxidant enzyme activity, and impairs synaptic function(Boscolo et al., 2013). Propofol uncouples oxidative phosphorylation, disrupting mitochondrial function and increasing ROS production, thereby exacerbating neurotoxicity, which is difficult to mitigate through pretreatment(Shibuta et al., 2022). When midazolam is combined with nitrous oxide and isoflurane, ROS levels increase by approximately 30%, and antioxidant enzyme activity is notably reduced(Boscolo et al., 2013). Anesthetic compounds—particularly benzodiazepines such as midazolam and diazepam—have been associated with detrimental neurodevelopmental consequences, primarily through the disruption of synaptic architecture, impairment of synaptic transmission, and induction of neuronal apoptosis(Lee et al., 2015).

Exposure to sevoflurane has been demonstrated to activate the PI3K/Akt signaling cascade, thereby perturbing the balance between pro-survival and pro-apoptotic pathways and triggering apoptotic cell death. This dysregulation enhances MOMP, ultimately triggering intrinsic apoptotic pathways and contributing to long-term cognitive impairments observed during adolescence (Hu et al., 2019; Yu et al., 2020). In parallel, mounting evidence implicates ER stress as a key mediator of sevoflurane-induced neurotoxicity, primarily via the PERK–eIF2α–ATF4–CHOP signaling pathway (B. Liu et al., 2017). In the developing brain, exposure to sevoflurane has also been shown to upregulate the expression of protein tyrosine phosphatase 1B within the ER, thereby initiating ER stress responses and promoting neurodegenerative alterations (Liu et al., 2019).

2.5 Effects of Anesthetics Targeting $GABA_A$ Rs on Non-Coding RNAs (ncRNAs)

Emerging evidence highlights the pivotal influence of ncRNAs in orchestrating diverse neurobiological processes through intricate post-transcriptional regulatory mechanisms. General

anesthetics modulate the expression profiles of microRNAs (miRNAs) and long non-coding RNAs (lncRNAs) in the developing brain, forming complex molecular regulatory networks that may perturb critical neural pathways. Such dysregulation is implicated in the induction of acute neuronal apoptosis and persistent memory deficits, potentially resulting in irreversible neurodevelopmental alterations(Jiang et al., 2021; Rodrigues et al., 2020; Shu et al., 2019).

With respect to apoptotic mechanisms, sevoflurane has been shown to upregulate the expression of caspase-3 and Bax, while concurrently downregulating Bcl-2, BDNF, and nerve growth factor (NGF). These changes are accompanied by alterations in long non-coding RNA (lncRNA) expression and a reduction in hippocampal neuronal density, collectively leading to ultrastructural abnormalities and neuronal apoptosis (Hu et al., 2019) (Hu et al., 2019). Moreover, the long non-coding RNA LRCF has been identified as a pivotal regulatory element in mediating isoflurane-induced apoptosis of oligodendrocytes.. In neonatal mice, expression levels of LRCF are significantly elevated relative to those in adults. This upregulation promotes apoptotic signaling through activation of the HIF-1α/caspase-3 axis. Conversely, reduced LRCF expression appears to exert neuroprotective effects by recruiting the alternative HIF-1α/miR-138-5p/caspase-3 pathway. These expression-dependent regulatory divergences may account for the heightened susceptibility of the developing brain to anesthesia-induced neurotoxicity during the neonatal period. Consequently, high LRCF expression may serve as a high-risk biomarker for OLG damage caused by anesthesia(Zeng et al., 2021). Exposure to sevoflurane has been demonstrated to influence synaptic plasticity by upregulating the expression of synaptophysin mRNA, a key presynaptic marker, via a mechanism dependent on N6-methyladenosine RNA modification. Paradoxically, this post-transcriptional modification contributes to a subsequent decline in synaptophysin protein abundance, which is associated with disrupted motor coordination and impaired cognitive performance in juvenile mice(L. Zhang et al., 2022). Isoflurane anesthesia has been reported to downregulate the expression of microRNA-132, thereby reducing dendritic spine density in the hippocampus, a morphological alteration that contributes to impaired learning and memory performance in rodent models.(Zhang et al., 2017).

3. Neuroprotective Potential of Anesthetics

NAS, as sedatives and anesthetics, demonstrate significant neuroprotective potential (Tateiwa & Evers, 2024). By modulating both inhibitory and excitatory neurotransmitter systems, NAS maintain the stability of neural networks. Additionally, NAS promote the release of BDNF, a molecule essential for supporting neuronal growth, viability, and synaptic plasticity. Furthermore, NAS regulate inflammation and oxidative stress, alleviating neuroinflammation and reducing oxidative damage, thereby effectively protecting neuronal cells from injury. These multiple mechanisms collectively highlight the potential of NAS agents as neurodevelopmental protective compounds (Fig. 3).

3.1 Modulation of the Inhibitory Neurotransmitter System

While most NAS serve as potent allosteric modulators of $GABA_AR$, enhancing or inhibiting GABA-induced currents via subtype-specific binding—such as potentiation by 3α-hydroxylated neurosteroids and suppression by 3β-sulfated derivatives—emerging evidence indicates a divergence in neurotoxic potential among $GABA_AR$-activating agents(Chen et al., 2019; Laverty et al., 2017). Notably,

compounds like propofol, CDNC24, and alphaxalone all activate $GABA_A$Rs, yet only propofol has been consistently associated with significant neurotoxicity during brain development, whereas NAS such as alphaxalone exhibit negligible or no overt neurotoxic effects(Tesic et al., 2020). Further investigations revealed that NAS compounds, such as alphaxalone and 3β-OH, do not induce neuronal apoptosis, regardless of their activation of $GABA_A$Rs. The neuroprotective properties of these compounds may be partially attributed to their ability to inhibit T-type calcium channels, particularly Cav3.1, thereby diminishing presynaptic GABA release(Tesic et al., 2020; Timic Stamenic et al., 2021). Intriguingly, the mechanism of action of 3β-OH appears to operate independently of classical modulation of GABAergic or NMDA receptor pathways(Atluri et al., 2018; Timic Stamenic et al., 2021). NAS enhances neuronal regeneration. For instance, allopregnanolone binds to the transmembrane domain of the $GABA_A$R complex, activating the receptor and increasing intracellular Ca^{2+} concentrations. This calcium influx subsequently activates CaMK IV, which phosphorylates and activates the transcription factor CREB1. Once activated, CREB1 enhances the transcription of genes involved in cell cycle regulation, thereby promoting mitotic activity in neural stem cells and oligodendrocyte precursor cells(G. F. Li et al., 2018). Moreover, NAS demonstrates significant potential for selective regulation of different $GABA_A$R subtypes. These compounds can modulate receptor tonic currents by enhancing $GABA_A$R-mediated current activity. At moderate concentrations, NAS compounds inhibit $GABA_A$R function, whereas at lower and higher concentrations, they enhance GABA regulation. This suggests that NAS compounds exert a complex bidirectional effect on $GABA_A$Rs within different concentration ranges, potentially providing a new therapeutic strategy for neuroprotection by modulating neural activity (Fig. 4).

3.2 Modulation of the Excitatory Neurotransmitter System

NAS, such as 3β-OH, reduces presynaptic AMPA receptor-mediated excitatory synaptic currents but does not affect NMDAR-mediated excitatory currents, thus modulating the glutamatergic system(Atluri et al., 2018). Gonadal steroids, such as estradiol (E2) and 17β-estradiol, potentiate NMDAR-mediated synaptic transmission and facilitate the release of GLU from primary afferent terminals, thereby increasing dendritic spine density in dorsal horn neurons of the spinal cord(Zhang et al., 2012). Estradiol not only enhances the binding of NMDAR agonists and competitive antagonists(Kow et al., 2005) but may also exert a dual action by enhancing cognitive function, promoting synaptic transmission while offering neuroprotective effects(Nilsen et al., 2002). The enantiomer of 17β-estradiol exhibits neuroprotective properties against glutamate-induced toxicity in cultured neuronal cells, implying that its protective effects may be mediated through pathways independent of classical nuclear estrogen receptors(Green et al., 2001). These studies suggest that NAS drugs exhibit neuroprotective potential by modulating NMDARs and the GLU transport system within a specific concentration range(Fig. 4).

3.3 Promotion of BDNF Release

Many studies have indicated that NAS can bind to and regulate various proteins involved in cell protection, including estrogen receptors α/β (ERα/β), the orphan nuclear receptor pregnane-X receptor (PXR), and the GPER1, thereby modulating BDNF levels(Briz & Baudry, 2014; Jakab et al., 2001; Serrao & Goodchild, 2022). For example, the enantiomer of 17β-estradiol regulates BDNF levels through an ERβ-mediated mechanism(Jakab et al., 2001). This modulatory action not only contributes to improved learning and memory performance but also serves a pivotal role in mitigating cognitive impairments induced by anesthesia(Taylor et al., 2017). Additionally, neurosteroid drugs such as alphaxalone activate PXR, further promoting the secretion of BDNF and exerting neuroprotective effects(Frye et al., 2014; Serrao & Goodchild, 2022). Clinical randomized controlled trials (RCTs) have demonstrated that patients treated with alphaxalone during hip replacement surgery show better cognitive performance postoperatively, with significantly higher plasma levels of BDNF than patients treated with propofol and sevoflurane(Serrao & Goodchild, 2022). These findings suggest that NAS, by modulating BDNF and associated pathways, exhibits significant neuroprotective effects, particularly in the recovery of cognitive function following anesthesia and surgery.

3.4 Regulation of Inflammation and Oxidative Stress

NAS, such as pregnenolone, exert anti-inflammatory effects through multiple signaling pathways, including Toll-like receptor signaling, the TRAM-TRIF pathway, BDNF, and CX3CL1 signaling pathways. NAS suppress the initiation of pro-inflammatory signaling cascades by disrupting the binding of Toll-like receptors—specifically TLR2, TLR4, and TLR7—to their adaptor proteins such as MyD88, MD2, and TIRAP, thereby reducing downstream synthesis of pro-inflammatory mediators(Balan et al., 2019; Balan, Grusca, et al., 2023; Balan, Patterson, et al., 2023).In addition to its established role in suppressing pro-inflammatory signaling, NAS activate the TLR4–TRAM–TRIF pathway, leading to the upregulation of key anti-inflammatory mediators such as IL-10 and BDNF, thereby contributing to neuroprotection (Aurelian & Balan, 2019). NAS also promote the expression of the anti-inflammatory chemokine CX3CL1, which is essential for maintaining the equilibrium between pro- and anti-inflammatory responses within the central nervous system, ultimately alleviating neuroinflammation. Furthermore, NAS have been shown to attenuate microglial pro-inflammatory activation by engaging $GABA_A$Rs and modulating the BDNF–TrkA/TrkB signaling cascade, while concurrently regulating the transcription of pro-inflammatory genes (Alexaki et al., 2018; S. Y. Wu et al., 2020).Beyond anti-inflammatory action, NAS support neuronal repair and regeneration through diverse neuroprotective mechanisms, offering potential therapeutic benefits in the context of neurodegenerative conditions and traumatic brain injury. Collectively, these findings underscore the central role of inflammation and oxidative stress in anesthetic-induced neurotoxicity. Importantly, NAS modulate antioxidant and anti-inflammatory processes not only through GABAergic pathways but also via alternative non-GABAergic mechanisms, highlighting their promise in mitigating the adverse neurodevelopmental effects associated with anesthetic exposure.

4. Susceptibility Factors for Anesthetic-Induced Neurotoxicity

4.1. Dose-, Time-, and Frequency-Dependent Effects of Anesthetics

Anesthetic-induced neurotoxicity demonstrates marked dependence on dose, exposure duration, and administration frequency. Evidence from both cellular and animal models indicates that anesthetic agents, such as propofol, may facilitate the differentiation of neural progenitor cells into neurons when administered at very low concentrations(Qiao et al., 2017). However, at subclinical or medium-to-high doses, these anesthetics inhibit neuronal proliferation, increase pro-inflammatory factor levels, and trigger widespread neuronal apoptosis, with the extent of apoptosis gradually increasing with higher doses(K. Chen et al., 2022; Han et al., 2015). Anesthetic neurotoxicity also demonstrates clear time dependence, with neuronal apoptosis worsening as the exposure duration increases(Huang et al., 2012; Zhao et al., 2016). In rodent models, brief exposure to propofol (2 or 4 hours) induced marked activation of caspase-3, a key executor of apoptosis. Notably, a more prolonged exposure of 6 hours elicited not only a pronounced increase in caspase-3 but also elevated calpain activity within the cerebral cortex and thalamus. These molecular alterations were concomitant with the emergence of apoptotic neuronal profiles, indicating that extended anesthetic exposure may potentiate neurodegenerative processes in specific brain regions(Milanovic et al., 2010). Prolonged exposure to sevoflurane impairs the maturation of neural circuits by inhibiting synaptogenesis and destabilizing dendritic spine development. This anesthetic disruption is further characterized by structural synaptic alterations, including increased expression of synaptic vesicle-related proteins, reduced density of apical dendritic spines, and ultrastructural disorganization within hippocampal synapses, which collectively contribute to cognitive deficits observed in juvenile rats(Yu et al., 2020). Moreover, a study comparing single-dose versus seven-dose administrations of propofol with the same total dose showed that rats exposed multiple times exhibited significant impairments in spatial learning and hippocampal function, suggesting that frequent exposures may have a more severe impact on neurological function than a single cumulative dose(Gao et al., 2014). Recurrent administration of sevoflurane during critical periods of cortical development has been implicated in long-term synaptic dysfunction, accompanied by structural synaptic anomalies and persistent cognitive deficits manifesting in adulthood(G. Li et al., 2019; Zhou et al., 2019)

4.2 Synergistic Effects of Combined Anesthesia

Emerging evidence indicates that the combined administration of anesthetic agents may markedly potentiate neuronal apoptosis and disrupt hippocampal synaptic integrity, thereby impairing learning and memory through synergistic neurotoxic effects(Jevtovic-Todorovic et al., 2003). For instance, during critical windows of brain maturation, the combined administration of ketamine with thiopental or propofol significantly amplifies neuronal cell death in neonatal animals and results in enduring functional impairments in adulthood, whereas the administration of these agents individually tends to produce only modest neurodevelopmental effects(Fredriksson et al., 2007). The combined use of anesthetics such as isoflurane, midazolam, and nitric oxide (NO) in a "triple cocktail" may reduce synaptic density(Lunardi et al., 2010), destabilize mitochondria(Sanchez et al., 2011), and increase autophagic activity(Fredriksson et al., 2007), which could contribute to neurodegenerative changes. Combined exposure to midazolam and nitrous oxide perturbs the homeostasis of mitochondrial dynamics by dysregulating key modulators such as Mfn-2 and Drp-1. This imbalance leads to pronounced mitochondrial morphological alterations, compromised bioenergetic function, and subsequent impairments in neuronal integrity and performance(Boscolo et al., 2013).

4.3 Region-Specific Sensitivity to Anesthetics During Critical Periods of Brain Development

During BGS, exposure to anesthetic drugs can lead to widespread neuronal apoptosis and result in persistent behavioral deficits(Gentry et al., 2013). The temporal window of the BGS varies significantly across mammalian species. In rodents, this critical phase of neurodevelopment unfolds within the first two weeks following birth (Semple et al., 2013), whereas in rhesus macaques, it spans from gestational day 115 to approximately postnatal day 60 (Brambrink et al., 2012); and in humans, the growth spurts occur at varying times across different brain regions, with most regions peaking in the second year after birth and typically reaching their maximum between 3 and 10 years of age(Andropoulos & Greene, 2017). Neuronal apoptosis induced by anesthetic drugs exhibits significant regional specificity at different time points during the BGS. In rodents, postnatal day 0 (P0) is the peak period of early apoptosis, primarily occurring in neurons of the hypothalamus and certain thalamic nuclei; day 3 (P3) marks the peak of intermediate apoptosis, affecting regions such as the Subiculum, Hibrait, caudate nucleus, and thalamic nuclei; day 7 (P7) represents the peak of late apoptosis, predominantly affecting cortical neurons(Maloney et al., 2019). Similarly, In a comparable model using rhesus monkeys, prenatal exposure to propofol predominantly induces apoptosis in neurons and oligodendrocytes within the cerebellum, hypothalamus, and additional posterior and anterior brain regions, whereas exposure during the neonatal period primarily affects the cerebral cortex(Creeley et al., 2013) (Fig. 4).

4.4 Vulnerable Populations for Anesthetic-Induced Neurotoxicity

Neonates—particularly those born prematurely, with low birth weight, reduced gestational age, or complex congenital anomalies—constitute a highly vulnerable population, exhibiting an elevated susceptibility to anesthesia-related neurodevelopmental impairment. Neuroimaging studies have revealed that preterm infants frequently display structural abnormalities in both gray and white matter following neonatal surgery. These alterations, detectable by MRI, have been strongly correlated with subsequent cognitive deficits and delays in neurodevelopmental milestones(Filan et al., 2012; Stolwijk et al., 2017). Weiss et al. noted that neonatal brains are highly sensitive to ischemic injury, placing these groups at a higher risk for neurodevelopmental disorders during the perioperative period. Specifically, during anesthesia, changes in hemodynamics, respiration, and metabolism may have a greater impact on neurodevelopment than the anesthetic drugs themselves(Weiss et al., 2016). Furthermore, factors such as increased surgical frequency, prolonged hospital stays, and prolonged respiratory support further exacerbate the risk of neurodevelopmental disorders. These impairments are not only related to the direct effects of surgery and anesthesia, but may also be closely associated with hemodynamic instability, improper respiratory control, and metabolic imbalance during anesthesia, compounded by surgical stress and primary diseases(Stolwijk et al., 2016).

5 Potential Strategies to Prevent and Reverse Neurotoxicity

5.1 Combination of Newer Anesthetic Agents

Dexmedetomidine (DEX), a highly selective α_2-adrenergic receptor agonist, has emerged as a potent neuroprotective agent against isoflurane-induced neurotoxicity, primarily due to its robust anti-apoptotic

properties. Mechanistically, DEX exerts its neuroprotective effects through engagement with several converging molecular cascades. These include the DNMT3a–miR-377-5p–Arc axis and the ERK1/2–CREB–BDNF signaling pathway (Xiao et al., 2018), along with activation of the PI3K–Akt cascade. Concurrently, it inhibits pro-apoptotic processes by downregulating the GSK-3β–CRMP2 and CDK5–CRMP2 pathways (Z. Chen et al., 2022). Pretreatment with DEX has also been shown to counteract the isoflurane-mediated repression of neurotrophic mediators such as BDNF mRNA, phosphorylated ERK1/2, CREB, and BDNF protein, thereby preserving synaptic homeostasis under anesthetic-induced stress(Tu et al., 2019). Additional studies have confirmed that the suppression of the GSK-3β/CRMP2 and CDK5/CRMP2 pathways by DEX contributes to reduced neuronal apoptosis and cognitive decline in neonatal models (J. Li et al., 2019). Moreover, the neuroprotection afforded by DEX extends beyond apoptosis, encompassing antioxidative and anti-inflammatory actions that mitigate sevoflurane-induced cognitive impairments (Wang et al., 2015). In neonatal rats, DEX may also confer protection via modulation of glutamatergic transmission (Wang et al., 2019). Activation of α_2-adrenergic receptors by DEX and related agonists like clonidine has additionally been found to attenuate tau hyperphosphorylation and its associated cognitive sequelae in neonatal mice exposed to sevoflurane (Sun et al., 2021). Notably, the neuroprotective efficacy of DEX in anesthetic regimens appears dose-dependent: at low concentrations, co-administration with sevoflurane enhances neuroprotection; however, higher doses paradoxically increase apoptosis and mortality, potentially due to reduced α_2 receptor selectivity and inadvertent α_1 receptor activation (Liu et al., 2016; Perez-Zoghbi et al., 2020) (Perez-Zoghbi et al., 2017)(Fig. 4).

Novel BZDs such as remimazolam, when combined with sevoflurane, significantly reduce the incidence of postoperative delirium, suggesting a neuroprotective potential that may be linked to reduced overall anesthetic exposure(Yang et al., 2022). The combined use of anesthetics might also protect the brain by balancing excitatory and inhibitory pathways. For example, midazolam could facilitate the reuptake of excess GLU via EAAT2 and enhance $GABA_AR$ activity, inhibiting the release of glutamate transporters, thereby effectively alleviating excitotoxicity caused by the upregulation of NMDAR NR1 subunits after ketamine withdrawal(Y. Li et al., 2018; Slikker et al., 2007). Moreover, NAS such as 17β-estradiol may enhance AKT phosphorylation, increase p-GSK-3β levels, and stimulate BDNF release(W. Li et al., 2019; Yang et al., 2022), effectively mitigating the apoptosis induced by combinations of triple cocktails(Lu et al., 2006) or ketamine(Li et al., 2014). Alphaxalone has been reported to mitigate the neurotoxic effects elicited by isoflurane exposure in neonatal rats, highlighting its potential as a neuroprotective agent during early brain development(Zhao et al., 2023).

5.2 Neuroprotective Agents

5.2.1 Anti-Apoptotic Agents

Neuronal apoptosis is a major mechanism of developmental neurotoxicity induced by $GABA_AR$ receptor agonists. Inhibiting apoptotic pathways may serve as a potential intervention. The histamine H_3 receptor antagonist Clobenpropit has been shown to activate the PI3K/Akt signaling cascade, thereby mitigating isoflurane-induced apoptotic injury in hippocampal neurons in vitro (He et al., 2018) (He et al., 2018). Complementarily, the TNF-α inhibitor Etanercept significantly reduced neuronal apoptosis in

the developing rat brain following isoflurane exposure (Chen et al., 2016) (Chen et al., 2016), suggesting a role for inflammatory modulation in anesthetic-induced neurotoxicity. In a separate study, Straiko and colleagues demonstrated that the co-administration of lithium with either ketamine or propofol did not significantly alter caspase-3 activation levels compared to control conditions, indicating a limited effect of lithium in modulating anesthetic-induced apoptosis via this pathway(Straiko et al., 2009).Moreover, erythropoietin (EPO) can partially reverse the decrease in BDNF and Bcl-2 levels caused by general anesthetics, thereby mitigating neuronal damage(Pellegrini et al., 2014).

5.2.2 Anti-Inflammatory Agents

Neuroinflammation has emerged as a critical mediator of anesthetic-induced neurotoxicity, making it a compelling target for therapeutic intervention. Recent findings indicate that edaravone confers neuroprotection against isoflurane-induced neuronal injury by attenuating inflammatory and apoptotic responses via activation of the mBDNF–TrkB–PI3K signaling axis (Yang et al., 2021). These beneficial effects have been validated across both in vitro neuronal culture systems and in vivo preclinical models, highlighting the therapeutic promise of neurotrophic pathway modulation in mitigating anesthesia-related neural damage. In parallel, doxycycline—a semi-synthetic tetracycline antibiotic—has demonstrated potent neuroprotective capabilities beyond its antimicrobial function. Through engagement of the PI3K/Akt pathway, doxycycline has been shown to exert significant anti-inflammatory and anti-apoptotic effects, supporting its candidacy as a therapeutic agent for inflammation-related neurodevelopmental pathologies (Möller et al., 2016).

5.2.3 Anti-Oxidative Stress Neuroprotective Agents

Recent advances in neuropharmacology underscore the neuroprotective potential of natural antioxidants against anesthesia-induced neurotoxicity. Melatonin, for instance, has been shown to activate the PKCα/Nrf2 signaling cascade, upregulate the anti-apoptotic protein Bcl-xL, suppress cytochrome c release, and inhibit mitochondrial-dependent apoptotic pathways. Through these mechanisms, melatonin markedly reduces neuronal apoptosis triggered by combined exposure to midazolam, nitrous oxide, and isoflurane, suggesting a protective role in anesthetic neurotoxicity (B. Li et al., 2018; Yon et al., 2006). Similarly, curcumin (Ji et al., 2015) and resveratrol (Tang et al., 2021), both known for their antioxidant properties, have been demonstrated to attenuate sevoflurane-induced oxidative stress by neutralizing ROS and suppressing associated inflammatory responses. Acetyl-L-carnitine (L-Ca), a mitochondria-targeted antioxidant, has been identified as neuroprotective in aging and neurodegenerative conditions (Robinson et al., 2019; Yan et al., 2014). Notably, co-administration of L-Ca with propofol has been reported to alleviate propofol-induced cytotoxicity in embryonic neural stem cells, primarily by reducing ROS production and downregulating oxidative stress-related signaling pathways (Liu et al., 2014). These findings highlight the promise of L-Ca as a potential adjunct in anesthetic protocols, particularly those involving agents with known oxidative liabilities.

5.2.4 Nutritional Supplements for Neuroprotection

Calcium homeostasis disruption is a key mechanism underlying anesthetic-induced neurotoxicity, involving mitochondrial dysfunction as well as damage to astrocytes and neurons. Emerging evidence indicates that stabilizing intracellular calcium dynamics may represent a promising strategy to counteract the neurodevelopmental toxicity associated with sevoflurane exposure(Zhu et al., 2024). Iron deficiency has been recognized as a pivotal determinant of impaired myelination within the central nervous system, exerting its most detrimental effects during the fetal period and early stages of postnatal development. Disruptions in iron homeostasis during these sensitive periods may compromise oligodendrocyte function and hinder the proper formation of myelin sheaths(Ward et al., 2014). Sevoflurane exposure has been implicated in disrupting myelin development during early brain maturation, potentially through mechanisms involving oligodendrocyte apoptosis, as well as deficiencies in iron and folate metabolism. Supplementing with iron and folate and inhibiting OL apoptosis could be effective preventive measures, although further research is needed.

These findings suggest that strategies aimed at preventing apoptosis and inflammation, reducing oxidative stress, and restoring calcium homeostasis and nutrient balance may offer promising interventions against sevoflurane-induced neurotoxicity (Fig. 3).

6. Concluding Remarks and Future Directions

During the BGS, the finely tuned interplay between the GABAergic and glutamatergic (GABA/GLU) systems is essential for proper neural development, with the developmental switch from NKCC1 to KCC2 transporters serving as a pivotal mechanism in establishing the balance between E/I signaling. Anesthetics, particularly intravenous agents targeting $GABA_A$Rs, may disrupt this transition, thereby destabilizing neural networks(Cabrera et al., 2020). Additional mechanisms include the imbalance of the BDNF signaling pathway, oxidative stress responses, and inflammatory reactions, all of which exacerbate neurotoxicity. Compared with traditional anesthetics, newer agents such as NAS(Tesic et al., 2020; Timic Stamenic et al., 2021), remimazolam(Shi et al., 2024), and DEX(Wei et al., 2016) exhibit milder mechanisms of action, maintaining sufficient anesthetic effects while minimizing interference with the nervous system. However, our understanding remains limited regarding how novel NAS and similar compounds may modulate T-type calcium channels and selectively bind to specific sites on $GABA_A$R, thereby facilitating endogenous regenerative processes in the brain and mitigating the neurotoxic effects of anesthetic agents. Further investigation is essential to elucidate their potential in alleviating neurotoxicity. Concurrently, significant strides are being made in the development of neuroprotective pharmacotherapies. Agents such as melatonin (B. Li et al., 2018), erythropoietin (EPO) (Pellegrini et al., 2014), and resveratrol (Tang et al., 2021)—all of which exhibit potent antioxidant, anti-inflammatory, and neuroprotective properties—have been investigated as promising candidates for mitigating anesthetic-induced neurotoxicity (Fig. 3).Accordingly, the development of next-generation anesthetic agents should prioritise strategies that minimise neurotoxicity, enhance intrinsic neuroprotective mechanisms, and facilitate post-injury repair and functional recovery—thereby ensuring both the safety and therapeutic efficacy of anaesthesia in paediatric and other high-risk populations.

Since the 2016 advisory issued by the FDA regarding the potential neurotoxic effects of anesthetic agents in pediatric populations, there has been a marked increase in clinical investigations evaluating the

neurodevelopmental consequences of anesthetic exposure in children. Findings from key studies such as the PANDA study (Sun et al., 2016) and the GAS trial (Davidson et al., 2016; McCann et al., 2019) have shown that a single, brief exposure—typically under one hour—to general anesthesia in children younger than five years of age is not associated with significant long-term neurodevelopmental deficits. However, the MASK study provided evidence that multiple exposures prior to age three may be linked to slower cognitive processing, impaired fine motor coordination, and increased risk of behavioral and learning difficulties. Supporting this, recent data suggest that general anesthesia administered to infants under one year of age is associated with an elevated risk of developmental delays, with risk escalating as the number of surgical procedures increases (Kobayashi et al., 2020). While the majority of clinical studies suggest that isolated anesthetic exposure is relatively safe, the literature remains inconclusive due to ethical constraints, methodological limitations, and perioperative confounders. Notably, the combined neurotoxic effects of multiple anesthetic agents and their potential protective mechanisms remain underexplored. Future research should adopt an integrative approach, combining mechanistic molecular studies, behavioral analyses in animal models, and rigorously designed clinical trials. Prospective randomized controlled trials (RCTs) with long-term neurodevelopmental follow-up are essential. Moreover, the establishment of standardized dosing frameworks and exposure durations, alongside anesthesia protocols tailored to regional brain vulnerability across developmental stages, will be critical to advancing this field

Clinical investigations into the long-term cognitive and behavioral consequences of anesthetic exposure in children employ heterogeneous methodologies. However, a significant proportion of these studies rely predominantly on subjective outcome assessments (Maloney et al., 2019), with limited incorporation of objective neuroimaging techniques or biomarker analyses, thereby constraining a comprehensive evaluation of anesthetic-induced neurodevelopmental effects (Bethlehem et al., 2022; H. Zhang et al., 2022). Notably, quantitative ultrasound imaging has revealed increased apoptotic activity in the developing brains of non-human primates following sevoflurane exposure, highlighting the potential neurotoxic impact of this agent during vulnerable periods of brain maturation (Rosado-Mendez et al., 2019). Complementary neuroimaging studies suggest that pediatric exposure to inhalational anesthetics such as isoflurane and sevoflurane is associated with cognitive impairments and disruptions in white matter integrity (Banerjee et al., 2019; Bethlehem et al., 2022). Moreover, prospective investigations report that as many as 75% of infants undergoing non-cardiac surgery for congenital anomalies exhibit mild to moderate neuroimaging abnormalities postoperatively (Mongerson et al., 2019; Moran et al., 2019). To delineate the precise neurodevelopmental impact of anesthetic agents, future studies should adopt an integrative approach—combining MRI and diffusion tensor imaging (DTI) with high-resolution ultrasound and biomarker profiling (e.g., inflammatory cytokines, BDNF)—in conjunction with longitudinal assessments of cognitive and behavioral function across defined developmental windows. This will help establish preventive or reversal strategies, such as the use of antioxidants, calcium channel blockers, or agents that promote myelination, or through environmental stimulation and functional training to restore the function of impaired neural networks.

7. conclusion

With the widespread clinical application of intravenous anesthetics targeting $GABA_A$Rs, their potential impact on the developing nervous system has increasingly become a focal point of academic attention. Although extensive research has elucidated the neurodevelopmental impact of $GABA_A$R–targeting agents via modulation of both $GABA_A$Rs and NMDA receptors, the specific influence of anesthetic drugs on synaptogenesis remains a complex and multilayered phenomenon. Deciphering the long-term consequences of such agents, alongside the identification of potential neuroprotective strategies and the refinement of their clinical applicability, continues to pose substantial challenges for future investigations.

Statements & Declarations

Acknowledgements

We sincerely thank all colleagues and collaborators whose insights and discussions contributed to the development of this manuscript. We are also grateful to the editorial office and anonymous reviewers for their constructive feedback, which helped us improve the clarity and rigor of our work.

Funding

This research was funded by the National Natural Science Foundation of China (82271288, 82471288).

Confict of interest

The authors declare no competing interests.

Contributors

Yubao Li: Writing–review & editing, Writing–original draft, Conceptualization, Visualization. Hongliang Yang: Visualization, Investigation, Methodology, Validation. Lu Liu: Formal analysis, Investigation. Lulu Jiang: Methodology, Supervision. Peilin Xie: Supervision. Xiaoling Wang: Investigation. Xuhui Cong: Formal analysis. Ruilou Zhu: Project administration. Zhongyuan Lu: Project administration. Jiaqiang Zhang: Writing–review & editing, Supervision, Project administration, Funding acquisition, Investigation, Conceptualization, Resources. Mingyang Sun: Writing–review & editing, Supervision, Project administration, Funding acquisition, Investigation.

Data availability

No new datasets were generated or analyzed in the course of this review. As such, data sharing is not applicable.

Ethical Approval

This work is a synthesis of previously published literature and does not involve any original research involving human participants or animals conducted by the authors. All referenced studies received appropriate ethical approvals as documented in their respective publications.

Sources and Data Illustration Strategy

To illustrate the mechanisms of anesthetic-induced neurotoxicity, all pathway diagrams and synaptic models presented in this review were created using BioRender and manually curated from previously published peer-reviewed literature. No new experimental or clinical data were generated or analyzed for this work.

References

Alexaki, V. I., Fodelianaki, G., Neuwirth, A., Mund, C., Kourgiantaki, A., Ieronimaki, E., Lyroni, K., Troullinaki, M., Fujii, C., Kanczkowski, W., Ziogas, A., Peitzsch, M., Grossklaus, S., Sönnichsen, B., Gravanis, A., Bornstein, S. R., Charalampopoulos, I., Tsatsanis, C., & Chavakis, T. (2018). DHEA inhibits acute microglia-mediated inflammation through activation of the TrkA-Akt1/2-CREB-Jmjd3 pathway. *Mol Psychiatry*, *23*(6), 1410-1420. https://doi.org/10.1038/mp.2017.167

Andropoulos, D. B., & Greene, M. F. (2017). Anesthesia and Developing Brains - Implications of the FDA Warning. *N Engl J Med*, *376*(10), 905-907. https://doi.org/10.1056/NEJMp1700196

Atluri, N., Joksimovic, S. M., Oklopcic, A., Milanovic, D., Klawitter, J., Eggan, P., Krishnan, K., Covey, D. F., Todorovic, S. M., & Jevtovic-Todorovic, V. (2018). A neurosteroid analogue with T-type calcium channel blocking properties is an effective hypnotic, but is not harmful to neonatal rat brain. *Br J Anaesth*, *120*(4), 768-778. https://doi.org/10.1016/j.bja.2017.12.039

Aurelian, L., & Balan, I. (2019). GABA(A)R α2-activated neuroimmune signal controls binge drinking and impulsivity through regulation of the CCL2/CX3CL1 balance. *Psychopharmacology (Berl)*, *236*(10), 3023-3043. https://doi.org/10.1007/s00213-019-05220-4

Bademosi, A. T., Steeves, J., Karunanithi, S., Zalucki, O. H., Gormal, R. S., Liu, S., Lauwers, E., Verstreken, P., Anggono, V., Meunier, F. A., & van Swinderen, B. (2018). Trapping of Syntaxin1a in Presynaptic Nanoclusters by a Clinically Relevant General Anesthetic. *Cell Rep*, *22*(2), 427-440. https://doi.org/10.1016/j.celrep.2017.12.054

Balan, I., Beattie, M. C., O'Buckley, T. K., Aurelian, L., & Morrow, A. L. (2019). Endogenous Neurosteroid (3α,5α)3-Hydroxypregnan-20-one Inhibits Toll-like-4 Receptor Activation and Pro-inflammatory Signaling in Macrophages and Brain. *Sci Rep*, *9*(1), 1220. https://doi.org/10.1038/s41598-018-37409-6

Balan, I., Grusca, A., O'Buckley, T. K., & Morrow, A. L. (2023). Neurosteroid [3α,5α]-3-hydroxy-pregnan-20-one enhances IL-10 production via endosomal TRIF-dependent TLR4 signaling pathway. *Front Endocrinol (Lausanne)*, *14*, 1299420. https://doi.org/10.3389/fendo.2023.1299420

Balan, I., Patterson, R., Boero, G., Krohn, H., O'Buckley, T. K., Meltzer-Brody, S., & Morrow, A. L. (2023). Brexanolone therapeutics in post-partum depression involves inhibition of systemic inflammatory pathways. *EBioMedicine*, *89*, 104473. https://doi.org/10.1016/j.ebiom.2023.104473

Banerjee, P., Rossi, M. G., Anghelescu, D. L., Liu, W., Breazeale, A. M., Reddick, W. E., Glass, J. O., Phillips, N. S., Jacola, L. M., Sabin, N. D., Inaba, H., Srivastava, D., Robison, L. L., Pui, C. H., Hudson, M. M., & Krull, K. R. (2019). Association Between Anesthesia Exposure and Neurocognitive and Neuroimaging Outcomes in Long-term Survivors of Childhood Acute Lymphoblastic Leukemia. *JAMA Oncol*, *5*(10), 1456-1463. https://doi.org/10.1001/jamaoncol.2019.1094

Bethlehem, R. A. I., Seidlitz, J., White, S. R., Vogel, J. W., Anderson, K. M., Adamson, C., Adler, S., Alexopoulos, G. S., Anagnostou, E., Areces-Gonzalez, A., Astle, D. E., Auyeung, B., Ayub, M., Bae, J., Ball, G., Baron-Cohen, S., Beare, R., Bedford, S. A., Benegal, V.,…Alexander-Bloch, A. F.

(2022). Brain charts for the human lifespan. *Nature*, *604*(7906), 525-533. https://doi.org/10.1038/s41586-022-04554-y

Bi, C., Cai, Q., Shan, Y., Yang, F., Sun, S., Wu, X., & Liu, H. (2018). Sevoflurane induces neurotoxicity in the developing rat hippocampus by upregulating connexin 43 via the JNK/c-Jun/AP-1 pathway. *Biomed Pharmacother*, *108*, 1469-1476. https://doi.org/10.1016/j.biopha.2018.09.111

Boscolo, A., Milanovic, D., Starr, J. A., Sanchez, V., Oklopcic, A., Moy, L., Ori, C. C., Erisir, A., & Jevtovic-Todorovic, V. (2013). Early exposure to general anesthesia disturbs mitochondrial fission and fusion in the developing rat brain. *Anesthesiology*, *118*(5), 1086-1097. https://doi.org/10.1097/ALN.0b013e318289bc9b

Brambrink, A. M., Back, S. A., Riddle, A., Gong, X., Moravec, M. D., Dissen, G. A., Creeley, C. E., Dikranian, K. T., & Olney, J. W. (2012). Isoflurane-induced apoptosis of oligodendrocytes in the neonatal primate brain. *Ann Neurol*, *72*(4), 525-535. https://doi.org/10.1002/ana.23652

Briz, V., & Baudry, M. (2014). Estrogen Regulates Protein Synthesis and Actin Polymerization in Hippocampal Neurons through Different Molecular Mechanisms. *Front Endocrinol (Lausanne)*, *5*, 22. https://doi.org/10.3389/fendo.2014.00022

Cabrera, O. H., Tesic, V., Tat, Q. L., Chastain, S., Quillinan, N., & Jevtovic-Todorovic, V. (2020). Sevoflurane-Induced Dysregulation of Cation-Chloride Cotransporters NKCC1 and KCC2 in Neonatal Mouse Brain. *Mol Neurobiol*, *57*(1), 1-10. https://doi.org/10.1007/s12035-019-01751-1

Calvo-Rodriguez, M., & Bacskai, B. J. (2021). Mitochondria and Calcium in Alzheimer's Disease: From Cell Signaling to Neuronal Cell Death. *Trends Neurosci*, *44*(2), 136-151. https://doi.org/10.1016/j.tins.2020.10.004

Chen, B., Deng, X., Wang, B., & Liu, H. (2016). Etanercept, an inhibitor of TNF-a, prevents propofol-induced neurotoxicity in the developing brain. *Int J Dev Neurosci*, *55*, 91-100. https://doi.org/10.1016/j.ijdevneu.2016.10.002

Chen, J., Zhang, D. M., Feng, X., Wang, J., Qin, Y. Y., Zhang, T., Huang, Q., Sheng, R., Chen, Z., Li, M., & Qin, Z. H. (2018). TIGAR inhibits ischemia/reperfusion-induced inflammatory response of astrocytes. *Neuropharmacology*, *131*, 377-388. https://doi.org/10.1016/j.neuropharm.2018.01.012

Chen, K., Lu, D., Yang, X., Zhou, R., Lan, L., Wu, Y., Wang, C., Xu, X., Jiang, M. H., Wei, M., & Feng, X. (2022). Enhanced hippocampal neurogenesis mediated by PGC-1α-activated OXPHOS after neonatal low-dose Propofol exposure. *Front Aging Neurosci*, *14*, 925728. https://doi.org/10.3389/fnagi.2022.925728

Chen, Z., Ding, Y., Zeng, Y., Zhang, X. P., & Chen, J. Y. (2022). Dexmedetomidine reduces propofol-induced hippocampal neuron injury by modulating the miR-377-5p/Arc pathway. *BMC Pharmacol Toxicol*, *23*(1), 18. https://doi.org/10.1186/s40360-022-00555-9

Chen, Z. W., Bracamontes, J. R., Budelier, M. M., Germann, A. L., Shin, D. J., Kathiresan, K., Qian, M. X., Manion, B., Cheng, W. W. L., Reichert, D. E., Akk, G., Covey, D. F., & Evers, A. S. (2019). Multiple functional neurosteroid binding sites on GABAA receptors. *PLoS Biol*, *17*(3), e3000157. https://doi.org/10.1371/journal.pbio.3000157

Choi, E. H., Blasiak, A., Lee, J., & Yang, I. H. (2019). Modulation of Neural Activity for Myelination in the Central Nervous System. *Front Neurosci*, *13*, 952. https://doi.org/10.3389/fnins.2019.00952

Cohen-Cory, S., Kidane, A. H., Shirkey, N. J., & Marshak, S. (2010). Brain-derived neurotrophic factor and the development of structural neuronal connectivity. *Dev Neurobiol*, *70*(5), 271-288. https://doi.org/10.1002/dneu.20774

Creeley, C., Dikranian, K., Dissen, G., Martin, L., Olney, J., & Brambrink, A. (2013). Propofol-induced apoptosis of neurones and oligodendrocytes in fetal and neonatal rhesus macaque brain. *Br J Anaesth*, *110 Suppl 1*(Suppl 1), i29-38. https://doi.org/10.1093/bja/aet173

Davidson, A. J., Disma, N., de Graaff, J. C., Withington, D. E., Dorris, L., Bell, G., Stargatt, R., Bellinger, D. C., Schuster, T., Arnup, S. J., Hardy, P., Hunt, R. W., Takagi, M. J., Giribaldi, G., Hartmann, P. L., Salvo, I., Morton, N. S., von Ungern Sternberg, B. S., Locatelli, B. G.,…McCann, M. E. (2016). Neurodevelopmental outcome at 2 years of age after general anaesthesia and awake-regional anaesthesia in infancy (GAS): an international multicentre, randomised controlled trial. *Lancet*, *387*(10015), 239-250. https://doi.org/10.1016/s0140-6736(15)00608-x

Deoni, S., Dean, D., 3rd, Joelson, S., O'Regan, J., & Schneider, N. (2018). Early nutrition influences developmental myelination and cognition in infants and young children. *Neuroimage*, *178*, 649-659. https://doi.org/10.1016/j.neuroimage.2017.12.056

DiGruccio, M. R., Joksimovic, S., Joksovic, P. M., Lunardi, N., Salajegheh, R., Jevtovic-Todorovic, V., Beenhakker, M. P., Goodkin, H. P., & Todorovic, S. M. (2015). Hyperexcitability of rat thalamocortical networks after exposure to general anesthesia during brain development. *J Neurosci*, *35*(4), 1481-1492. https://doi.org/10.1523/jneurosci.4883-13.2015

Diviccaro, S., Cioffi, L., Falvo, E., Giatti, S., & Melcangi, R. C. (2022). Allopregnanolone: An overview on its synthesis and effects. *J Neuroendocrinol*, *34*(2), e12996. https://doi.org/10.1111/jne.12996

Doi, A., Miyazaki, T., Mihara, T., Ikeda, M., Niikura, R., Andoh, T., & Goto, T. (2021). CLP290 promotes the sedative effects of midazolam in neonatal rats in a KCC2-dependent manner: A laboratory study in rats. *PLoS One*, *16*(3), e0248113. https://doi.org/10.1371/journal.pone.0248113

Dong, Y., Hong, W., Tang, Z., Gao, Y., Wu, X., & Liu, H. (2020). Sevoflurane leads to learning and memory dysfunction via breaking the balance of tPA/PAI-1. *Neurochem Int*, *139*, 104789. https://doi.org/10.1016/j.neuint.2020.104789

Fan, X. Y., Shi, G., & Zhao, P. (2021). Neonatal Sevoflurane Exposure Impairs Learning and Memory by the Hypermethylation of Hippocampal Synaptic Genes. *Mol Neurobiol*, *58*(3), 895-904. https://doi.org/10.1007/s12035-020-02161-4

Faraco, G., Hochrainer, K., Segarra, S. G., Schaeffer, S., Santisteban, M. M., Menon, A., Jiang, H., Holtzman, D. M., Anrather, J., & Iadecola, C. (2019). Dietary salt promotes cognitive impairment through tau phosphorylation. *Nature*, *574*(7780), 686-690. https://doi.org/10.1038/s41586-019-1688-z

Filan, P. M., Hunt, R. W., Anderson, P. J., Doyle, L. W., & Inder, T. E. (2012). Neurologic outcomes in very preterm infants undergoing surgery. *J Pediatr*, *160*(3), 409-414. https://doi.org/10.1016/j.jpeds.2011.09.009

Food, U., & Administration, D. (2017). FDA Drug Safety Communication: FDA review results in new warnings about using general anesthetics and sedation drugs in young children and pregnant women. *Silver Spring, MD, USA: US Food and Drug Administration*.

Forcelli, P. A., Kim, J., Kondratyev, A., & Gale, K. (2011). Pattern of antiepileptic drug-induced cell death in limbic regions of the neonatal rat brain. *Epilepsia*, *52*(12), e207-211. https://doi.org/10.1111/j.1528-1167.2011.03297.x

Fredriksson, A., Pontén, E., Gordh, T., & Eriksson, P. (2007). Neonatal exposure to a combination of N-methyl-D-aspartate and gamma-aminobutyric acid type A receptor anesthetic agents potentiates apoptotic neurodegeneration and persistent behavioral deficits. *Anesthesiology*, *107*(3), 427-436. https://doi.org/10.1097/01.anes.0000278892.62305.9c

Frye, C. A., Koonce, C. J., & Walf, A. A. (2014). Novel receptor targets for production and action of allopregnanolone in the central nervous system: a focus on pregnane xenobiotic receptor. *Front Cell Neurosci*, *8*, 106. https://doi.org/10.3389/fncel.2014.00106

Gao, J., Peng, S., Xiang, S., Huang, J., & Chen, P. (2014). Repeated exposure to propofol impairs spatial learning, inhibits LTP and reduces CaMKIIα in young rats. *Neurosci Lett*, *560*, 62-66. https://doi.org/10.1016/j.neulet.2013.11.061

Gentry, K. R., Steele, L. M., Sedensky, M. M., & Morgan, P. G. (2013). Early developmental exposure to volatile anesthetics causes behavioral defects in Caenorhabditis elegans. *Anesth Analg*, *116*(1), 185-189. https://doi.org/10.1213/ANE.0b013e31826d37c5

Green, P. S., Yang, S. H., Nilsson, K. R., Kumar, A. S., Covey, D. F., & Simpkins, J. W. (2001). The nonfeminizing enantiomer of 17beta-estradiol exerts protective effects in neuronal cultures and a rat model of cerebral ischemia. *Endocrinology*, *142*(1), 400-406. https://doi.org/10.1210/endo.142.1.7888

Han, D., Jin, J., Fang, H., & Xu, G. (2015). Long-term action of propofol on cognitive function and hippocampal neuroapoptosis in neonatal rats. *Int J Clin Exp Med*, *8*(7), 10696-10704.

He, W., Yuan, Q. H., & Zhou, Q. (2018). Histamine H3 receptor antagonist Clobenpropit protects propofol-induced apoptosis of hippocampal neurons through PI3K/AKT pathway. *Eur Rev Med Pharmacol Sci*, *22*(22), 8013-8020. https://doi.org/10.26355/eurrev_201811_16430

Head, B. P., Patel, H. H., Niesman, I. R., Drummond, J. C., Roth, D. M., & Patel, P. M. (2009). Inhibition of p75 neurotrophin receptor attenuates isoflurane-mediated neuronal apoptosis in the neonatal central nervous system. *Anesthesiology*, *110*(4), 813-825. https://doi.org/10.1097/ALN.0b013e31819b602b

Hirotsu, A., Iwata, Y., Tatsumi, K., Miyai, Y., Matsuyama, T., & Tanaka, T. (2019). Maternal exposure to volatile anesthetics induces IL-6 in fetal brains and affects neuronal development. *Eur J Pharmacol*, *863*, 172682. https://doi.org/10.1016/j.ejphar.2019.172682

Hu, X., Hu, X., & Huang, G. (2019). LncRNA MALAT1 is involved in sevoflurane-induced neurotoxicity in developing rats. *J Cell Biochem*, *120*(10), 18209-18218. https://doi.org/10.1002/jcb.29127

Huang, L., Liu, Y., Jin, W., Ji, X., & Dong, Z. (2012). Ketamine potentiates hippocampal neurodegeneration and persistent learning and memory impairment through the PKCγ-ERK signaling pathway in the developing brain. *Brain Res*, *1476*, 164-171. https://doi.org/10.1016/j.brainres.2012.07.059

Jakab, R. L., Wong, J. K., & Belcher, S. M. (2001). Estrogen receptor beta immunoreactivity in differentiating cells of the developing rat cerebellum. *J Comp Neurol*, *430*(3), 396-409. https://doi.org/10.1002/1096-9861(20010212)430:3<396::aid-cne1039>3.0.co;2-0

Jevtovic-Todorovic, V., Hartman, R. E., Izumi, Y., Benshoff, N. D., Dikranian, K., Zorumski, C. F., Olney, J. W., & Wozniak, D. F. (2003). Early exposure to common anesthetic agents causes widespread neurodegeneration in the developing rat brain and persistent learning deficits. *J Neurosci*, *23*(3), 876-882. https://doi.org/10.1523/jneurosci.23-03-00876.2003

Ji, M. H., Qiu, L. L., Yang, J. J., Zhang, H., Sun, X. R., Zhu, S. H., Li, W. Y., & Yang, J. J. (2015). Pre-administration of curcumin prevents neonatal sevoflurane exposure-induced neurobehavioral abnormalities in mice. *Neurotoxicology*, *46*, 155-164. https://doi.org/10.1016/j.neuro.2014.11.003

Jiang, C., Arzua, T., Yan, Y., & Bai, X. (2021). Expression Signature of lncRNAs and mRNAs in Sevoflurane-Induced Mouse Brain Injury: Implication of Involvement of Wide Molecular Networks and Pathways. *Int J Mol Sci*, *22*(3). https://doi.org/10.3390/ijms22031389

Ju, L. S., Yang, J. J., Gravenstein, N., Seubert, C. N., Morey, T. E., Sumners, C., Vasilopoulos, T., Yang, J. J., & Martynyuk, A. E. (2017). Role of environmental stressors in determining the developmental outcome of neonatal anesthesia. *Psychoneuroendocrinology*, *81*, 96-104. https://doi.org/10.1016/j.psyneuen.2017.04.001

Kahraman, S., Zup, S. L., McCarthy, M. M., & Fiskum, G. (2008). GABAergic mechanism of propofol toxicity in immature neurons. *J Neurosurg Anesthesiol*, *20*(4), 233-240. https://doi.org/10.1097/ANA.0b013e31817ec34d

Karunanithi, S., Cylinder, D., Ertekin, D., Zalucki, O. H., Marin, L., Lavidis, N. A., Atwood, H. L., & van Swinderen, B. (2020). Proportional Downscaling of Glutamatergic Release Sites by the General Anesthetic Propofol at Drosophila Motor Nerve Terminals. *eNeuro*, *7*(1). https://doi.org/10.1523/eneuro.0422-19.2020

Kobayashi, Y., Tokuda, N., Adachi, S., Takeshima, Y., Hirose, M., & Shima, M. (2020). Association between surgical procedures under general anesthesia in infancy and developmental outcomes at 1 year: the Japan Environment and Children's Study. *Environ Health Prev Med*, *25*(1), 32. https://doi.org/10.1186/s12199-020-00873-6

Kow, L. M., Easton, A., & Pfaff, D. W. (2005). Acute estrogen potentiates excitatory responses of neurons in rat hypothalamic ventromedial nucleus. *Brain Res*, *1043*(1-2), 124-131. https://doi.org/10.1016/j.brainres.2005.02.068

Laverty, D., Thomas, P., Field, M., Andersen, O. J., Gold, M. G., Biggin, P. C., Gielen, M., & Smart, T. G. (2017). Crystal structures of a GABA(A)-receptor chimera reveal new endogenous neurosteroid-binding sites. *Nat Struct Mol Biol*, *24*(11), 977-985. https://doi.org/10.1038/nsmb.3477

Lee, J. H., Zhang, J., Wei, L., & Yu, S. P. (2015). Neurodevelopmental implications of the general anesthesia in neonate and infants. *Exp Neurol*, *272*, 50-60. https://doi.org/10.1016/j.expneurol.2015.03.028

Li, B., Feng, X. J., Hu, X. Y., Chen, Y. P., Sha, J. C., Zhang, H. Y., & Fan, H. G. (2018). Effect of melatonin on attenuating the isoflurane-induced oxidative damage is related to PKCα/Nrf2 signaling pathway in developing rats. *Brain Res Bull*, *143*, 9-18. https://doi.org/10.1016/j.brainresbull.2018.09.018

Li, G., Du, J., Wang, L., & Shi, X. (2019). Developmental neurotoxicity in the context of multiple sevoflurane exposures: Potential role of histone deacetylase 6. *Neurotoxicol Teratol*, *74*, 106813. https://doi.org/10.1016/j.ntt.2019.106813

Li, G. F., Li, Z. B., Zhuang, S. J., & Li, G. C. (2018). Inhibition of microRNA-34a protects against propofol anesthesia-induced neurotoxicity and cognitive dysfunction via the MAPK/ERK signaling pathway. *Neuroscience Letters*, *675*, 152-159. https://doi.org/https://doi.org/10.1016/j.neulet.2018.03.052

Li, J., Guo, M., Liu, Y., Wu, G., Miao, L., Zhang, J., Zuo, Z., & Li, Y. (2019). Both GSK-3β/CRMP2 and CDK5/CRMP2 Pathways Participate in the Protection of Dexmedetomidine Against Propofol-Induced Learning and Memory Impairment in Neonatal Rats. *Toxicol Sci*, *171*(1), 193-210. https://doi.org/10.1093/toxsci/kfz135

Li, J., Wang, B., Wu, H., Yu, Y., Xue, G., & Hou, Y. (2014). 17β-estradiol attenuates ketamine-induced neuroapoptosis and persistent cognitive deficits in the developing brain. *Brain Res*, *1593*, 30-39. https://doi.org/10.1016/j.brainres.2014.09.013

Li, W., Li, H., Wei, H., Lu, Y., Lei, S., Zheng, J., Lu, H., Chen, X., Liu, Y., & Zhang, P. (2019). 17β-Estradiol Treatment Attenuates Neurogenesis Damage and Improves Behavior Performance After Ketamine Exposure in Neonatal Rats. *Front Cell Neurosci*, *13*, 251. https://doi.org/10.3389/fncel.2019.00251

Li, Y., Chen, L., Zhu, D., Chen, Y., Qin, W., & Cui, J. (2020). Propofol downregulates the activity of glutamatergic neurons in the basal forebrain via affecting intrinsic membrane properties and postsynaptic GABAARs. *Neuroreport*, *31*(17), 1242-1248. https://doi.org/10.1097/wnr.0000000000001540

Li, Y., Li, X., Zhao, J., Li, L., Wang, Y., Zhang, Y., Chen, Y., Liu, W., & Gao, L. (2018). Midazolam Attenuates Autophagy and Apoptosis Caused by Ketamine by Decreasing Reactive Oxygen Species in the Hippocampus of Fetal Rats. *Neuroscience*, *388*, 460-471. https://doi.org/10.1016/j.neuroscience.2018.03.040

Liao, Z., Huang, Z., Li, J., Li, H., Miao, L., Liu, Y., Zhang, J., Xu, Y., & Li, Y. (2021). Regulation of CRMP2 by Cdk5 and GSK-3β participates in sevoflurane-induced dendritic development abnormalities and cognitive dysfunction in developing rats. *Toxicol Lett*, *341*, 68-79. https://doi.org/10.1016/j.toxlet.2021.01.023

Liu, B., Ou, G., Chen, Y., & Zhang, J. (2019). Inhibition of protein tyrosine phosphatase 1B protects against sevoflurane-induced neurotoxicity mediated by ER stress in developing brain. *Brain Res Bull*, *146*, 28-39. https://doi.org/10.1016/j.brainresbull.2018.12.006

Liu, B., Xia, J., Chen, Y., & Zhang, J. (2017). Sevoflurane-Induced Endoplasmic Reticulum Stress Contributes to Neuroapoptosis and BACE-1 Expression in the Developing Brain: The Role of eIF2α. *Neurotox Res*, *31*(2), 218-229. https://doi.org/10.1007/s12640-016-9671-z

Liu, F., Rainosek, S. W., Sadovova, N., Fogle, C. M., Patterson, T. A., Hanig, J. P., Paule, M. G., Slikker, W., Jr., & Wang, C. (2014). Protective effect of acetyl-L-carnitine on propofol-induced toxicity in embryonic neural stem cells. *Neurotoxicology*, *42*, 49-57. https://doi.org/10.1016/j.neuro.2014.03.011

Liu, J. R., Yuki, K., Baek, C., Han, X. H., & Soriano, S. G. (2016). Dexmedetomidine-Induced Neuroapoptosis Is Dependent on Its Cumulative Dose. *Anesth Analg*, *123*(4), 1008-1017. https://doi.org/10.1213/ane.0000000000001527

Liu, Y., Yan, Y., Inagaki, Y., Logan, S., Bosnjak, Z. J., & Bai, X. (2017). Insufficient Astrocyte-Derived Brain-Derived Neurotrophic Factor Contributes to Propofol-Induced Neuron Death Through Akt/Glycogen Synthase Kinase 3β/Mitochondrial Fission Pathway. *Anesth Analg*, *125*(1), 241-254. https://doi.org/10.1213/ane.0000000000002137

Lu, H., Liufu, N., Dong, Y., Xu, G., Zhang, Y., Shu, L., Soriano, S. G., Zheng, H., Yu, B., & Xie, Z. (2017). Sevoflurane Acts on Ubiquitination-Proteasome Pathway to Reduce Postsynaptic Density 95 Protein Levels in Young Mice. *Anesthesiology*, *127*(6), 961-975. https://doi.org/10.1097/aln.0000000000001889

Lu, L. X., Yon, J. H., Carter, L. B., & Jevtovic-Todorovic, V. (2006). General anesthesia activates BDNF-dependent neuroapoptosis in the developing rat brain. *Apoptosis*, *11*(9), 1603-1615. https://doi.org/10.1007/s10495-006-8762-3

Lunardi, N., Ori, C., Erisir, A., & Jevtovic-Todorovic, V. (2010). General anesthesia causes long-lasting disturbances in the ultrastructural properties of developing synapses in young rats. *Neurotox Res*, *17*(2), 179-188. https://doi.org/10.1007/s12640-009-9088-z

Maksimovic, S., Useinovic, N., Quillinan, N., Covey, D. F., Todorovic, S. M., & Jevtovic-Todorovic, V. (2022). General Anesthesia and the Young Brain: The Importance of Novel Strategies with Alternate Mechanisms of Action. *Int J Mol Sci*, *23*(3). https://doi.org/10.3390/ijms23031889

Maloney, S. E., Creeley, C. E., Hartman, R. E., Yuede, C. M., Zorumski, C. F., Jevtovic-Todorovic, V., Dikranian, K., Noguchi, K. K., Farber, N. B., & Wozniak, D. F. (2019). Using animal models to evaluate the functional consequences of anesthesia during early neurodevelopment. *Neurobiol Learn Mem*, *165*, 106834. https://doi.org/10.1016/j.nlm.2018.03.014

Marchi, S., Patergnani, S., Missiroli, S., Morciano, G., Rimessi, A., Wieckowski, M. R., Giorgi, C., & Pinton, P. (2018). Mitochondrial and endoplasmic reticulum calcium homeostasis and cell death. *Cell Calcium*, *69*, 62-72. https://doi.org/10.1016/j.ceca.2017.05.003

McCann, M. E., de Graaff, J. C., Dorris, L., Disma, N., Withington, D., Bell, G., Grobler, A., Stargatt, R., Hunt, R. W., Sheppard, S. J., Marmor, J., Giribaldi, G., Bellinger, D. C., Hartmann, P. L., Hardy, P., Frawley, G., Izzo, F., von Ungern Sternberg, B. S., Lynn, A.,...Davidson, A. J. (2019). Neurodevelopmental outcome at 5 years of age after general anaesthesia or awake-regional anaesthesia in infancy (GAS): an international, multicentre, randomised, controlled equivalence trial. *Lancet*, *393*(10172), 664-677. https://doi.org/10.1016/s0140-6736(18)32485-1

Mendiola, A. S., & Cardona, A. E. (2018). The IL-1β phenomena in neuroinflammatory diseases. *J Neural Transm (Vienna)*, *125*(5), 781-795. https://doi.org/10.1007/s00702-017-1732-9

Miao, H. H., Liu, W. B., Jiao, X. H., Shao, K. J., Yuan, Y. X., Sha, S., Zhang, Q. Q., Yan, J., Sun, Y. Y., Zhou, C. H., & Wu, Y. Q. (2022). Neonatal Exposure to Propofol Interferes with the Proliferation and Differentiation of Hippocampal Neural Stem Cells and the Neurocognitive Function of Rats in Adulthood via the Akt/p27 Signaling Pathway. *Biomed Environ Sci*, *35*(4), 283-295. https://doi.org/10.3967/bes2022.040

Milanovic, D., Pesic, V., Loncarevic-Vasiljkovic, N., Pavkovic, Z., Popic, J., Kanazir, S., Jevtovic-Todorovic, V., & Ruzdijic, S. (2016). The Fas Ligand/Fas Death Receptor Pathways Contribute to Propofol-Induced Apoptosis and Neuroinflammation in the Brain of Neonatal Rats. *Neurotox Res*, *30*(3), 434-452. https://doi.org/10.1007/s12640-016-9629-1

Milanovic, D., Popic, J., Pesic, V., Loncarevic-Vasiljkovic, N., Kanazir, S., Jevtovic-Todorovic, V., & Ruzdijic, S. (2010). Regional and temporal profiles of calpain and caspase-3 activities in postnatal rat brain following repeated propofol administration. *Dev Neurosci*, *32*(4), 288-301. https://doi.org/10.1159/000316970

Möller, T., Bard, F., Bhattacharya, A., Biber, K., Campbell, B., Dale, E., Eder, C., Gan, L., Garden, G. A., Hughes, Z. A., Pearse, D. D., Staal, R. G., Sayed, F. A., Wes, P. D., & Boddeke, H. W. (2016). Critical data-based re-evaluation of minocycline as a putative specific microglia inhibitor. *Glia*, *64*(10), 1788-1794. https://doi.org/10.1002/glia.23007

Mongerson, C. R. L., Wilcox, S. L., Goins, S. M., Pier, D. B., Zurakowski, D., Jennings, R. W., & Bajic, D. (2019). Infant Brain Structural MRI Analysis in the Context of Thoracic Non-cardiac Surgery and Critical Care. *Front Pediatr*, *7*, 315. https://doi.org/10.3389/fped.2019.00315

Moran, M. M., Gunn-Charlton, J. K., Walsh, J. M., Cheong, J. L. Y., Anderson, P. J., Doyle, L. W., Greaves, S., & Hunt, R. W. (2019). Associations of Neonatal Noncardiac Surgery with Brain Structure and Neurodevelopment: A Prospective Case-Control Study. *J Pediatr*, *212*, 93-101.e102. https://doi.org/10.1016/j.jpeds.2019.05.050

Naseri, M., Parham, A., & Moghimi, A. (2017). The effect of sodium thiopental as a GABA mimetic drug in neonatal period on expression of GAD65 and GAD67 genes in hippocampus of newborn and adult male rats. *Iran J Basic Med Sci*, *20*(9), 996-1001. https://doi.org/10.22038/ijbms.2017.9264

Nilsen, J., Chen, S., & Brinton, R. D. (2002). Dual action of estrogen on glutamate-induced calcium signaling: mechanisms requiring interaction between estrogen receptors and src/mitogen activated protein kinase pathway. *Brain Res*, *930*(1-2), 216-234. https://doi.org/10.1016/s0006-8993(02)02254-0

Oh, W. C., Lutzu, S., Castillo, P. E., & Kwon, H. B. (2016). De novo synaptogenesis induced by GABA in the developing mouse cortex. *Science*, *353*(6303), 1037-1040. https://doi.org/10.1126/science.aaf5206

Pellegrini, L., Bennis, Y., Velly, L., Grandvuillemin, I., Pisano, P., Bruder, N., & Guillet, B. (2014). Erythropoietin protects newborn rat against sevoflurane-induced neurotoxicity. *Paediatr Anaesth*, *24*(7), 749-759. https://doi.org/10.1111/pan.12372

Perez-Zoghbi, J. F., Zhu, W., Grafe, M. R., & Brambrink, A. M. (2017). Dexmedetomidine-mediated neuroprotection against sevoflurane-induced neurotoxicity extends to several brain regions in neonatal rats. *Br J Anaesth*, *119*(3), 506-516. https://doi.org/10.1093/bja/aex222

Perez-Zoghbi, J. F., Zhu, W., Neudecker, V., Grafe, M. R., & Brambrink, A. M. (2020). Neurotoxicity of sub-anesthetic doses of sevoflurane and dexmedetomidine co-administration in neonatal rats. *Neurotoxicology*, *79*, 75-83. https://doi.org/10.1016/j.neuro.2020.03.014

Piao, M., Wang, Y., Liu, N., Wang, X., Chen, R., Qin, J., Ge, P., & Feng, C. (2020). Sevoflurane Exposure Induces Neuronal Cell Parthanatos Initiated by DNA Damage in the Developing Brain via an Increase of Intracellular Reactive Oxygen Species. *Front Cell Neurosci*, *14*, 583782. https://doi.org/10.3389/fncel.2020.583782

Popić, J., Pešić, V., Milanović, D., Lončarević-Vasiljković, N., Smiljanić, K., Kanazir, S., & Ruždijić, S. (2015). Induction of TNF-α signaling cascade in neonatal rat brain during propofol anesthesia. *Int J Dev Neurosci*, *44*, 22-32. https://doi.org/10.1016/j.ijdevneu.2015.05.003

Qiao, H., Li, Y., Xu, Z., Li, W., Fu, Z., Wang, Y., King, A., & Wei, H. (2017). Propofol Affects Neurodegeneration and Neurogenesis by Regulation of Autophagy via Effects on Intracellular Calcium Homeostasis. *Anesthesiology*, *127*(3), 490-501. https://doi.org/10.1097/aln.0000000000001730

Ramirez, K., Niraula, A., & Sheridan, J. F. (2016). GABAergic modulation with classical benzodiazepines prevent stress-induced neuro-immune dysregulation and behavioral alterations. *Brain Behav Immun*, *51*, 154-168. https://doi.org/10.1016/j.bbi.2015.08.011

Robinson, B., Gu, Q., Ali, S. F., Dumas, M., & Kanungo, J. (2019). Ketamine-induced attenuation of reactive oxygen species in zebrafish is prevented by acetyl l-carnitine in vivo. *Neurosci Lett*, *706*, 36-42. https://doi.org/10.1016/j.neulet.2019.05.009

Rodrigues, D. C., Mufteev, M., Weatheritt, R. J., Djuric, U., Ha, K. C. H., Ross, P. J., Wei, W., Piekna, A., Sartori, M. A., Byres, L., Mok, R. S. F., Zaslavsky, K., Pasceri, P., Diamandis, P., Morris, Q., Blencowe, B. J., & Ellis, J. (2020). Shifts in Ribosome Engagement Impact Key Gene Sets in Neurodevelopment and Ubiquitination in Rett Syndrome. *Cell Rep*, *30*(12), 4179-4196.e4111. https://doi.org/10.1016/j.celrep.2020.02.107

Rosado-Mendez, I. M., Noguchi, K. K., Castañeda-Martinez, L., Kirvassilis, G., Wang, S. H., Manzella, F., Swiney, B. S., Masuoka, K., Capuano, S., III, Brunner, K. G., Crosno, K., Guerrero, Q. W., Whitson, H., Brambrink, A., Simmons, H. S., Mejia, A. F., Zagzebski, J. A., Hall, T. J., & Ikonomidou, C. (2019). Quantitative ultrasound and apoptotic death in the neonatal primate brain. *Neurobiol Dis*, *127*, 554-562. https://doi.org/10.1016/j.nbd.2019.03.032

Sanchez, V., Feinstein, S. D., Lunardi, N., Joksovic, P. M., Boscolo, A., Todorovic, S. M., & Jevtovic-Todorovic, V. (2011). General Anesthesia Causes Long-term Impairment of Mitochondrial Morphogenesis and Synaptic Transmission in Developing Rat Brain. *Anesthesiology*, *115*(5), 992-1002. https://doi.org/10.1097/ALN.0b013e3182303a63

Schaefer, M. L., Wang, M., Perez, P. J., Coca Peralta, W., Xu, J., & Johns, R. A. (2019). Nitric Oxide Donor Prevents Neonatal Isoflurane-induced Impairments in Synaptic Plasticity and Memory. *Anesthesiology*, *130*(2), 247-262. https://doi.org/10.1097/aln.0000000000002529

Schenning, K. J., Noguchi, K. K., Martin, L. D., Manzella, F. M., Cabrera, O. H., Dissen, G. A., & Brambrink, A. M. (2017). Isoflurane exposure leads to apoptosis of neurons and oligodendrocytes in 20- and 40-day old rhesus macaques. *Neurotoxicol Teratol*, *60*, 63-68. https://doi.org/10.1016/j.ntt.2016.11.006

Semple, B. D., Blomgren, K., Gimlin, K., Ferriero, D. M., & Noble-Haeusslein, L. J. (2013). Brain development in rodents and humans: Identifying benchmarks of maturation and vulnerability to injury across species. *Prog Neurobiol*, *106-107*, 1-16. https://doi.org/10.1016/j.pneurobio.2013.04.001

Serrao, J. M., & Goodchild, C. S. (2022). Alfaxalone anaesthesia increases brain derived neurotrophic factor levels and preserves postoperative cognition by activating pregnane-X receptors: an in vitro study and a double blind randomised controlled trial. *BMC Anesthesiol*, *22*(1), 401. https://doi.org/10.1186/s12871-022-01940-x

Shi, W., Wu, X., Yuan, C., Kuang, T., Xie, X., Gong, W., Li, F., Shen, L., Zhang, Y., & Liang, X. (2024). Effect of remimazolam toluene sulfonate on the cognitive function of juveniles and its mechanism of action. *Eur J Med Res*, *29*(1), 543. https://doi.org/10.1186/s40001-024-02142-6

Shibuta, S., Morita, T., & Kosaka, J. (2022). Effect of preconditioning on propofol-induced neurotoxicity during the developmental period. *PLoS One*, *17*(8), e0273219. https://doi.org/10.1371/journal.pone.0273219

Shu, P., Wu, C., Liu, W., Ruan, X., Liu, C., Hou, L., Zeng, Y., Fu, H., Wang, M., Chen, P., Zhang, X., Yin, B., Yuan, J., Qiang, B., & Peng, X. (2019). The spatiotemporal expression pattern of microRNAs in the developing mouse nervous system. *J Biol Chem*, *294*(10), 3444-3453. https://doi.org/10.1074/jbc.RA118.004390

Slikker, W., Jr., Zou, X., Hotchkiss, C. E., Divine, R. L., Sadovova, N., Twaddle, N. C., Doerge, D. R., Scallet, A. C., Patterson, T. A., Hanig, J. P., Paule, M. G., & Wang, C. (2007). Ketamine-induced neuronal cell death in the perinatal rhesus monkey. *Toxicol Sci*, *98*(1), 145-158. https://doi.org/10.1093/toxsci/kfm084

Soyalp, C., Oksuz, E., Gorgisen, G., Gulacar, I. M., Yasar, S., Tuncdemir, Y. E., & Delen, L. A. (2022). Role of Sedative-Hypnotic Agents in Neurodegeneration: Effects of Midazolam and Thiopental on Apoptosis and Oxidative Stress Expression in Neonatal and Adult Rat Brains. *Turk Neurosurg*, *32*(3), 378-385. https://doi.org/10.5137/1019-5149.Jtn.32324-20.2

Stolwijk, L. J., Keunen, K., de Vries, L. S., Groenendaal, F., van der Zee, D. C., van Herwaarden, M. Y. A., Lemmers, P. M. A., & Benders, M. (2017). Neonatal Surgery for Noncardiac Congenital Anomalies: Neonates at Risk of Brain Injury. *J Pediatr*, *182*, 335-341.e331. https://doi.org/10.1016/j.jpeds.2016.11.080

Stolwijk, L. J., Lemmers, P. M., Harmsen, M., Groenendaal, F., de Vries, L. S., van der Zee, D. C., Benders, M. J., & van Herwaarden-Lindeboom, M. Y. (2016). Neurodevelopmental Outcomes After Neonatal Surgery for Major Noncardiac Anomalies. *Pediatrics*, *137*(2), e20151728. https://doi.org/10.1542/peds.2015-1728

Straiko, M. M., Young, C., Cattano, D., Creeley, C. E., Wang, H., Smith, D. J., Johnson, S. A., Li, E. S., & Olney, J. W. (2009). Lithium protects against anesthesia-induced developmental neuroapoptosis. *Anesthesiology*, *110*(4), 862-868. https://doi.org/10.1097/ALN.0b013e31819b5eab

Sun, L. S., Li, G., Miller, T. L., Salorio, C., Byrne, M. W., Bellinger, D. C., Ing, C., Park, R., Radcliffe, J., Hays, S. R., DiMaggio, C. J., Cooper, T. J., Rauh, V., Maxwell, L. G., Youn, A., & McGowan, F. X. (2016). Association Between a Single General Anesthesia Exposure Before Age 36 Months and Neurocognitive Outcomes in Later Childhood. *Jama*, *315*(21), 2312-2320. https://doi.org/10.1001/jama.2016.6967

Sun, M., Dong, Y., Li, M., Zhang, Y., Liang, F., Zhang, J., Soriano, S. G., & Xie, Z. (2021). Dexmedetomidine and Clonidine Attenuate Sevoflurane-Induced Tau Phosphorylation and Cognitive Impairment in Young Mice via α-2 Adrenergic Receptor. *Anesth Analg*, *132*(3), 878-889. https://doi.org/10.1213/ane.0000000000005268

Szymanski, J., & Minichiello, L. (2022). NKCC1 Deficiency in Forming Hippocampal Circuits Triggers Neurodevelopmental Disorder: Role of BDNF-TrkB Signalling. *Brain Sci*, *12*(4). https://doi.org/10.3390/brainsci12040502

Tang, X. L., Wang, X., Fang, G., Zhao, Y. L., Yan, J., Zhou, Z., Sun, R., Luo, A. L., & Li, S. Y. (2021). Resveratrol ameliorates sevoflurane-induced cognitive impairment by activating the SIRT1/NF-κB pathway in neonatal mice. *J Nutr Biochem*, *90*, 108579. https://doi.org/10.1016/j.jnutbio.2020.108579

Tao, G., Zhang, J., Zhang, L., Dong, Y., Yu, B., Crosby, G., Culley, D. J., Zhang, Y., & Xie, Z. (2014). Sevoflurane induces tau phosphorylation and glycogen synthase kinase 3β activation in young mice. *Anesthesiology*, *121*(3), 510-527. https://doi.org/10.1097/aln.0000000000000278

Tateiwa, H., & Evers, A. S. (2024). Neurosteroids and their potential as a safer class of general anesthetics. *J Anesth*, *38*(2), 261-274. https://doi.org/10.1007/s00540-023-03291-4

Taylor, G. T., Manzella, F. M., Huffman, J., Cabrera, O. H., & Hoffman, J. (2017). Cognition in female rats after blocking conversion of androgens to estrogens. *Horm Behav*, *90*, 84-89. https://doi.org/10.1016/j.yhbeh.2017.02.011

Tesic, V., Joksimovic, S. M., Quillinan, N., Krishnan, K., Covey, D. F., Todorovic, S. M., & Jevtovic-Todorovic, V. (2020). Neuroactive steroids alphaxalone and CDNC24 are effective hypnotics and potentiators of GABA(A) currents, but are not neurotoxic to the developing rat brain. *Br J Anaesth*, *124*(5), 603-613. https://doi.org/10.1016/j.bja.2020.01.013

Thomason, E. J., Escalante, M., Osterhout, D. J., & Fuss, B. (2020). The oligodendrocyte growth cone and its actin cytoskeleton: A fundamental element for progenitor cell migration and CNS myelination. *Glia*, *68*(7), 1329-1346. https://doi.org/10.1002/glia.23735

Timic Stamenic, T., Feseha, S., Manzella, F. M., Wallace, D., Wilkey, D., Corrigan, T., Fiedler, H., Doerr, P., Krishnan, K., Raol, Y. H., Covey, D. F., Jevtovic-Todorovic, V., & Todorovic, S. M. (2021). The T-type calcium channel isoform Ca(v)3.1 is a target for the hypnotic effect of the anaesthetic neurosteroid (3β,5β,17β)-3-hydroxyandrostane-17-carbonitrile. *Br J Anaesth*, *126*(1), 245-255. https://doi.org/10.1016/j.bja.2020.07.022

Tu, Y., Liang, Y., Xiao, Y., Lv, J., Guan, R., Xiao, F., Xie, Y., & Xiao, Q. (2019). Dexmedetomidine attenuates the neurotoxicity of propofol toward primary hippocampal neurons in vitro via Erk1/2/CREB/BDNF signaling pathways. *Drug Des Devel Ther*, *13*, 695-706. https://doi.org/10.2147/dddt.S188436

Vanderhaeghen, P., & Polleux, F. (2023). Developmental mechanisms underlying the evolution of human cortical circuits. *Nat Rev Neurosci*, *24*(4), 213-232. https://doi.org/10.1038/s41583-023-00675-z

Wan, J., Shen, C. M., Wang, Y., Wu, Q. Z., Wang, Y. L., Liu, Q., Sun, Y. M., Cao, J. P., & Wu, Y. Q. (2021). Repeated exposure to propofol in the neonatal period impairs hippocampal synaptic plasticity and the recognition function of rats in adulthood. *Brain Res Bull*, *169*, 63-72. https://doi.org/10.1016/j.brainresbull.2021.01.007

Wang, N., & Wang, M. (2019). Dexmedetomidine suppresses sevoflurane anesthesia-induced neuroinflammation through activation of the PI3K/Akt/mTOR pathway. *BMC Anesthesiol*, *19*(1), 134. https://doi.org/10.1186/s12871-019-0808-5

Wang, W. Y., Jia, L. J., Luo, Y., Zhang, H. H., Cai, F., Mao, H., Xu, W. C., Fang, J. B., Peng, Z. Y., Ma, Z. W., Chen, Y. H., Zhang, J., Wei, Z., Yu, B. W., & Hu, S. F. (2016). Location- and Subunit-Specific NMDA Receptors Determine the Developmental Sevoflurane Neurotoxicity Through ERK1/2 Signaling. *Mol Neurobiol*, *53*(1), 216-230. https://doi.org/10.1007/s12035-014-9005-1

Wang, X., Shan, Y., Tang, Z., Gao, L., & Liu, H. (2019). Neuroprotective effects of dexmedetomidine against isoflurane-induced neuronal injury via glutamate regulation in neonatal rats. *Drug Des Devel Ther*, *13*, 153-160. https://doi.org/10.2147/dddt.S163197

Wang, X., Zhao, B., & Li, X. (2015). Dexmedetomidine attenuates isoflurane-induced cognitive impairment through antioxidant, anti-inflammatory and anti-apoptosis in aging rat. *Int J Clin Exp Med*, *8*(10), 17281-17288.

Ward, R. J., Zucca, F. A., Duyn, J. H., Crichton, R. R., & Zecca, L. (2014). The role of iron in brain ageing and neurodegenerative disorders. *Lancet Neurol*, *13*(10), 1045-1060. https://doi.org/10.1016/s1474-4422(14)70117-6

Watanabe, M., & Fukuda, A. (2015). Development and regulation of chloride homeostasis in the central nervous system. *Front Cell Neurosci*, *9*, 371. https://doi.org/10.3389/fncel.2015.00371

Wei, Y., Hu, J., Liang, Y., Zhong, Y., He, D., Qin, Y., Li, L., Chen, J., Xiao, Q., & Xie, Y. (2016). Dexmedetomidine pretreatment attenuates propofol-induced neurotoxicity in neuronal cultures from the rat hippocampus. *Mol Med Rep*, *14*(4), 3413-3420. https://doi.org/10.3892/mmr.2016.5628

Weiss, M., Hansen, T. G., & Engelhardt, T. (2016). Ensuring safe anaesthesia for neonates, infants and young children: what really matters. *Arch Dis Child*, *101*(7), 650-652. https://doi.org/10.1136/archdischild-2015-310104

Wu, S. Y., Pan, B. S., Tsai, S. F., Chiang, Y. T., Huang, B. M., Mo, F. E., & Kuo, Y. M. (2020). BDNF reverses aging-related microglial activation. *J Neuroinflammation*, *17*(1), 210. https://doi.org/10.1186/s12974-020-01887-1

Wu, Z., Xue, H., Gao, Q., & Zhao, P. (2020). Effects of early postnatal sevoflurane exposure on oligodendrocyte maturation and myelination in cerebral white matter of the rat. *Biomed Pharmacother*, *131*, 110733. https://doi.org/10.1016/j.biopha.2020.110733

Wu, Z., Zhang, Y., Zhang, Y., & Zhao, P. (2020). Sirtuin 2 Inhibition Attenuates Sevoflurane-Induced Learning and Memory Deficits in Developing Rats via Modulating Microglial Activation. *Cell Mol Neurobiol*, *40*(3), 437-446. https://doi.org/10.1007/s10571-019-00746-9

Xian, F., Li, Q., & Chen, Z. (2019). Overexpression of phosphoprotein enriched in astrocytes 15 reverses the damage induced by propofol in hippocampal neurons. *Mol Med Rep*, *20*(2), 1583-1592. https://doi.org/10.3892/mmr.2019.10412

Xiao, Y., Zhou, L., Tu, Y., Li, Y., Liang, Y., Zhang, X., Lv, J., Zhong, Y., & Xie, Y. (2018). Dexmedetomidine attenuates the propofol-induced long-term neurotoxicity in the developing brain of rats by enhancing the PI3K/Akt signaling pathway. *Neuropsychiatr Dis Treat*, *14*, 2191-2206. https://doi.org/10.2147/ndt.S169099

Xu, J., Mathena, R. P., Xu, M., Wang, Y., Chang, C., Fang, Y., Zhang, P., & Mintz, C. D. (2018). Early Developmental Exposure to General Anesthetic Agents in Primary Neuron Culture Disrupts Synapse Formation via Actions on the mTOR Pathway. *Int J Mol Sci*, *19*(8). https://doi.org/10.3390/ijms19082183

Yan, J., Huang, Y., Lu, Y., Chen, J., & Jiang, H. (2014). Repeated administration of ketamine can induce hippocampal neurodegeneration and long-term cognitive impairment via the ROS/HIF-1α pathway in developing rats. *Cell Physiol Biochem*, *33*(6), 1715-1732. https://doi.org/10.1159/000362953

Yang, F., Zhang, Y., Tang, Z., Shan, Y., Wu, X., & Liu, H. (2020). Hemin treatment protects neonatal rats from sevoflurane-induced neurotoxicity via the phosphoinositide 3-kinase/Akt pathway. *Life Sci*, *242*, 117151. https://doi.org/10.1016/j.lfs.2019.117151

Yang, M., Lian, N., Yu, Y., Wang, Y., Xie, K., & Yu, Y. (2020). Coenzyme Q10 alleviates sevoflurane-induced neuroinflammation by regulating the levels of apolipoprotein E and phosphorylated tau protein in mouse hippocampal neurons. *Mol Med Rep*, *22*(1), 445-453. https://doi.org/10.3892/mmr.2020.11131

Yang, M., & Wei, H. (2017). Anesthetic neurotoxicity: Apoptosis and autophagic cell death mediated by calcium dysregulation. *Neurotoxicol Teratol*, *60*, 59-62. https://doi.org/10.1016/j.ntt.2016.11.004

Yang, X., Lin, C., Chen, S., Huang, Y., Cheng, Q., & Yao, Y. (2022). Remimazolam for the Prevention of Emergence Delirium in Children Following Tonsillectomy and Adenoidectomy Under Sevoflurane Anesthesia: A Randomized Controlled Study. *Drug Des Devel Ther*, *16*, 3413-3420. https://doi.org/10.2147/dddt.S381611

Yang, Y., Yi, J., Pan, M., Hu, B., & Duan, H. (2021). Edaravone Alleviated Propofol-Induced Neurotoxicity in Developing Hippocampus by mBDNF/TrkB/PI3K Pathway. *Drug Des Devel Ther*, *15*, 1409-1422. https://doi.org/10.2147/dddt.S294557

Ye, X., Lian, Q., Eckenhoff, M. F., Eckenhoff, R. G., & Pan, J. Z. (2013). Differential general anesthetic effects on microglial cytokine expression. *PLoS One*, *8*(1), e52887. https://doi.org/10.1371/journal.pone.0052887

Yeh, F. L., Hansen, D. V., & Sheng, M. (2017). TREM2, Microglia, and Neurodegenerative Diseases. *Trends Mol Med*, *23*(6), 512-533. https://doi.org/10.1016/j.molmed.2017.03.008

Yon, J. H., Carter, L. B., Reiter, R. J., & Jevtovic-Todorovic, V. (2006). Melatonin reduces the severity of anesthesia-induced apoptotic neurodegeneration in the developing rat brain. *Neurobiol Dis*, *21*(3), 522-530. https://doi.org/10.1016/j.nbd.2005.08.011

Yu, Z., Wang, J., Wang, H., Wang, J., Cui, J., & Junzhang, P. (2020). Effects of Sevoflurane Exposure During Late Pregnancy on Brain Development and Beneficial Effects of Enriched Environment on Offspring Cognition. *Cell Mol Neurobiol*, *40*(8), 1339-1352. https://doi.org/10.1007/s10571-020-00821-6

Zeng, Z., Yao, J., Zhong, J., Fan, S., Xue, Y., Chen, X., Luo, Y., & Xue, S. (2021). The Role of the lncRNA-LRCF in Propofol-Induced Oligodendrocyte Damage in Neonatal Mouse. *Neurochem Res*, *46*(4), 778-791. https://doi.org/10.1007/s11064-020-03205-w

Zhang, H., Li, J., Su, X., Hu, Y., Liu, T., Ni, S., Li, H., Zuo, X. N., Fu, J., Yuan, T. F., & Yang, Z. (2022). Growth charts of brain morphometry for preschool children. *Neuroimage*, *255*, 119178. https://doi.org/10.1016/j.neuroimage.2022.119178

Zhang, L., Cheng, Y., Xue, Z., Li, J., Wu, N., Yan, J., Wang, J., Wang, C., Chen, W., Zhou, T., Qiu, Z., & Jiang, H. (2022). Sevoflurane impairs m6A-mediated mRNA translation and leads to fine motor and cognitive deficits. *Cell Biol Toxicol*, *38*(2), 347-369. https://doi.org/10.1007/s10565-021-09601-4

Zhang, S., Liang, Z., Sun, W., & Pei, L. (2017). Repeated propofol anesthesia induced downregulation of hippocampal miR-132 and learning and memory impairment of rats. *Brain Res*, *1670*, 156-164. https://doi.org/10.1016/j.brainres.2017.04.011

Zhang, Y., Xiao, X., Zhang, X. M., Zhao, Z. Q., & Zhang, Y. Q. (2012). Estrogen facilitates spinal cord synaptic transmission via membrane-bound estrogen receptors: implications for pain hypersensitivity. *J Biol Chem*, *287*(40), 33268-33281. https://doi.org/10.1074/jbc.M112.368142

Zhao, T., Li, C., Wei, W., Zhang, H., Ma, D., Song, X., & Zhou, L. (2016). Prenatal ketamine exposure causes abnormal development of prefrontal cortex in rat. *Sci Rep*, *6*, 26865. https://doi.org/10.1038/srep26865

Zhao, X., Li, Z., Guo, Y., Liu, Q., Qing, M., Sheng, Y., Chen, Y., Xie, L., & Zhou, Z. (2023). Alfaxalone Alleviates Neurotoxicity and Cognitive Impairment Induced by Isoflurane Anesthesia in Offspring Rats. *Anesth Analg*, *136*(6), 1206-1216. https://doi.org/10.1213/ane.0000000000006420

Zheng, H., Dong, Y., Xu, Z., Crosby, G., Culley, D. J., Zhang, Y., & Xie, Z. (2013). Sevoflurane anesthesia in pregnant mice induces neurotoxicity in fetal and offspring mice. *Anesthesiology*, *118*(3), 516-526. https://doi.org/10.1097/ALN.0b013e3182834d5d

Zhou, B., Chen, L., Liao, P., Huang, L., Chen, Z., Liao, D., Yang, L., Wang, J., Yu, G., Wang, L., Zhang, J., Zuo, Y., Liu, J., & Jiang, R. (2019). Astroglial dysfunctions drive aberrant synaptogenesis and social behavioral deficits in mice with neonatal exposure to lengthy general anesthesia. *PLoS Biol*, *17*(8), e3000086. https://doi.org/10.1371/journal.pbio.3000086

Zhou, Z. W., Shu, Y., Li, M., Guo, X., Pac-Soo, C., Maze, M., & Ma, D. (2011). The glutaminergic, GABAergic, dopaminergic but not cholinergic neurons are susceptible to anaesthesia-induced cell death in the rat developing brain. *Neuroscience*, *174*, 64-70. https://doi.org/10.1016/j.neuroscience.2010.10.009

Zhu, R., Liu, L., Mao, T., Wang, X., Li, Y., Li, T., Lv, S., Zeng, S., Fu, N., Li, N., Wang, Y., Sun, M., & Zhang, J. (2024). Mfn2 regulates mitochondria and mitochondria-associated endoplasmic reticulum membrane function in neurodegeneration induced by repeated sevoflurane exposure. *Exp Neurol*, *377*, 114807. https://doi.org/10.1016/j.expneurol.2024.114807

Zuo, Y., Li, B., Xie, J., Ma, Z., Thirupathi, A., Yu, P., Gao, G., Zhou, J., Zhou, C., Xu, H., Chang, Y., & Shi, Z. (2020). Sevoflurane anesthesia during pregnancy in mice induces cognitive impairment in the offspring by causing iron deficiency and inhibiting myelinogenesis. *Neurochem Int*, *135*, 104693. https://doi.org/10.1016/j.neuint.2020.104693

FIGURES LEGENDS

Fig. 1 Mechanisms of Developmental Neurotoxicity Induced by Anesthetics Targeting $GABA_A$ Receptors

Anesthetic drugs target $GABA_A$ receptors ($GABA_AR$) in immature neurons, leading to developmental neurotoxicity. Black arrows represent the direct effects of anesthetic drugs on immature neurons, while red arrows indicate the subsequent effects triggered by the inhibition of relevant signaling pathways. The underlying mechanisms include: GA upregulating NKCC1 and downregulating KCC2, thereby enhancing GABA-mediated excitatory signaling, which leads to excessive calcium influx and calcium overload. This overload then triggers neuroinflammation and exacerbates oxidative stress. Through NMDAR-mediated excitatory/inhibitory imbalance (E/I imbalance), synaptic plasticity is disrupted, with a reduction in PSD95 and related proteins, as well as inhibition of long-term potentiation (LTP). Furthermore, GA affects the BDNF signaling pathway by enhancing BDNF/ $P75^{NTR}$, inhibiting BDNF/TrkB signaling, and causing BDNF dysregulation. Simultaneously, it interferes with abnormal ncRNA and PTP-1B signaling pathways, inducing endoplasmic reticulum stress, which ultimately results in neuronal damage.

Fig. 2 The Effects of Anesthetics Targeting $GABA_A$ Receptors on Different Neuronal Populations

Anesthetic agents activate microglia, triggering the release of pro-inflammatory cytokines and M1 markers, which in turn exacerbate neuroinflammation. These drugs induce massive apoptosis in oligodendrocytes, leading to neuronal demyelination. Notably, iron and folate have been shown to mitigate this demyelination process. Furthermore, anesthetics interfere with astrocyte function via the ERK pathway, disrupting BDNF metabolism and ultimately leading to neuronal apoptosis. This cascade of events underscores the harmful effects of anesthetics on neurodevelopment, particularly in the context of inflammation-induced apoptosis in both neurons and glial cells.

Fig. 3 Neuroprotective Mechanisms of NAS

NAS exerts neuroprotective effects through multiple pathways. The mechanisms are as follows: black arrows indicate the direct effects of anesthetics on immature neurons, while red arrows represent subsequent effects induced by the inhibition of related signaling pathways. NAS interacts with TLR2, TLR4, and TLR7 and their adaptor proteins to reduce the production of inflammatory factors, thereby alleviating neuronal inflammation. NAS also regulates the synthesis of mBDNF through receptors such as GPER1, ERα/β, and PXR. mBDNF activates MEK/ERK, PI3K/AKT, and PLCγ/PKC signaling pathways through TrkA/B receptors, inhibiting ROS production, alleviating mitochondrial dysfunction, reducing neuronal apoptosis, enhancing neurotransmitter release, and activating NMDAR to promote LTP formation, thus increasing synaptic plasticity. Additionally, NAS may exert anesthetic effects by modulating $GABA_ARs$ and calcium ions while enhancing neuronal regeneration to further protect the nervous system. NAS, DEX, and EPO reduce neuroinflammation and exhibit anti-apoptotic and neuroprotective effects through the BDNF/TrkB pathway, while Clobenpropit, Edaravone, and Doxycycline act through the BDNF/PI3K pathway. Melatonin increases BDNF production through Nrf2, while L-Ca, Curcumin, and Resveratrol reduce oxidative stress, and Etanercept reduces the production of inflammatory factors.

Fig. 4. The Impact of Anesthetic Agents on Different Brain Regions During Development

Neurodevelopmental trajectories based on global pediatric MRI data are presented, with the y-axis representing the percentage of maximum volume attained by each brain region during development, and

the x-axis representing age. The yellow shaded region highlights the period of anesthesia vulnerability, in comparison to the critical peak developmental periods for various brain functions.The impact of anesthetic drugs on different brain regions is shown, with highlighted areas indicating regions particularly vulnerable to anesthesia-induced damage. BGS:brain growth spurt;GMV:total cortical grey matter volume;WMV:total white matter volume;CSF:total ventricular cerebrospinal fluid volume (ventricles or cerebrospinal fluid);MCT:mean cortical thickness;TSA:total surface area; TCV:total cerebrum volume.

Fig. 5 Graphical Abstract: Mechanisms of Anesthetic-Induced Neurotoxicity Targeting $GABA_A$ Receptors and Associated Susceptibility Factors

Neonatal brain development occurs during a critical period, and anesthetic-induced neurotoxicity during this time is influenced by multiple complex factors, including the mechanisms of anesthetic drugs targeting GABA receptors and associated susceptibility factors. The interplay of these factors contributes to the formation of a complex mechanism underlying neonatal neurotoxicity induced by anesthesia.

Fig. 1 Mechanisms of Developmental Neurotoxicity Induced by Anesthetics Targeting $GABA_A$ Receptors

1394 **Fig. 2** **The Effects of Anesthetics Targeting $GABA_A$ Receptors on Different Neuronal Populations**

Fig. 3 **Neuroprotective Mechanisms of NAS**

Fig. 4 The Impact of Anesthetic Agents on Different Brain Regions During Development

Fig. 5 Graphical Abstract: Mechanisms of Anesthetic-Induced Neurotoxicity Targeting $GABA_A$ Receptors and Associated Susceptibility Factors

Fig. 1-5 Created with BioRender.com.

1403

20250528222701112523091301041766

ORIGINALITY REPORT

14%

SIMILARITY INDEX

PRIMARY SOURCES

1	link.springer.com<br>Internet	110 words — 1%
2	www.frontiersin.org<br>Internet	72 words — 1%
3	Jing Zhang, Yu Li. "Propofol-Induced Developmental Neurotoxicity: From Mechanisms to Therapeutic Strategies", ACS Chemical Neuroscience, 2023<br>Crossref	34 words — < 1%
4	Mingyang Sun, Xiaoling Wang, Zhongyuan Lu, Yitian Yang, Shuang Lv, Mengrong Miao, Wan-Ming Chen, Szu-Yuan Wu, Jiaqiang Zhang. "Comparative effectiveness of SGLT2 inhibitors and GLP-1 receptor agonists in preventing Alzheimer's disease, vascular dementia, and other dementia types among patients with type 2 diabetes", Diabetes & Metabolism, 2025<br>Crossref	27 words — < 1%
5	Qiu-Xia Xiao, Min-Jian Geng, Qiu-Lin Wang, Chang-Le Fang, Jing-Han Zhang, Qi Liu, Liu-Liu Xiong. "Unraveling the Effects of Prenatal Anesthesia on Neurodevelopment: A Review of Current Evidence and Future Directions", NeuroToxicology, 2024<br>Crossref	25 words — < 1%
6	smarttots.org<br>Internet	24 words — < 1%

7	Atanu Bhattacharjee, Akula Ramakrishna, Magisetty Obulesu. "Phytomedicine and Alzheimer’s Disease", CRC Press, 2020<br>Publications	22 words — < 1%
8	Francesca M. Manzella, Douglas F. Covey, Vesna Jevtovic-Todorovic, Slobodan M. Todorovic. "Synthetic neuroactive steroids as new sedatives and anaesthetics: Back to the future", Journal of Neuroendocrinology, 2022<br>Crossref	22 words — < 1%
9	mdpi-res.com<br>Internet	22 words — < 1%
10	Ningning Fu, Yangyang Wang, Ruilou Zhu, Ningning Li, Shuang Zeng, Mengrong Miao, Yitian Yang, Mingyang Sun, Jiaqiang Zhang. "Bicuculline and Bumetanide Attenuate Sevoflurane-Induced Impairment of Myelination and Cognition in Young Mice", ACS Chemical Neuroscience, 2023<br>Crossref	21 words — < 1%
11	coek.info<br>Internet	20 words — < 1%
12	www.researchsquare.com<br>Internet	20 words — < 1%
13	pmc.ncbi.nlm.nih.gov<br>Internet	19 words — < 1%
14	Kantarakorn Unchiti, Prangmalee Leurcharusmee, Artid Samerchua, Tanyong Pipanmekaporn et al. " The Potential Role of Dexmedetomidine	18 words — < 1%

on Neuroprotection and its Possible Mechanisms: Evidence from and Studies ", European Journal of Neuroscience, 2021
Crossref

15 Thiago Arzua, Congshan Jiang, Yasheng Yan, Xiaowen Bai. "The importance of non-coding RNAs in environmental stress-related developmental brain disorders: A systematic review of evidence associated with exposure to alcohol, anesthetic drugs, nicotine, and viral infections", Neuroscience & Biobehavioral Reviews, 2021 — 18 words — < 1%
Crossref

16 Wen-Liang Yan, Wenliang Yan, Xitong Liang, Yang Li et al. "Genome-wide association analyses for revealing QTN, QTN-by-environment and QTN-by-QTN interactions in soybean phenology", Springer Science and Business Media LLC, 2024 — 18 words — < 1%
Crossref Posted Content

17 www2.mdpi.com — 17 words — < 1%
Internet

18 Rosalva Mora-Escobedo, Gloria Dávila-Ortiz, Gustavo F. Gutiérrez López, José J. Berrios. "Health-Promoting Food Ingredients during Processing", Routledge, 2024 — 16 words — < 1%
Publications

19 hdl.handle.net — 16 words — < 1%
Internet

20 onlinelibrary.wiley.com — 16 words — < 1%
Internet

21 www.jneurosci.org — 16 words — < 1%
Internet

22 Bonnie Robinson, Qiang Gu, Jyotshna Kanungo. "Antidepressant Actions of Ketamine: Potential Role of L-Type Calcium Channels", Chemical Research in Toxicology, 2021
Crossref
15 words — < 1%

23 Chao Ji, Qiang Ni, Wurong Chen, Zeyong Yang, Daqing Ma. "General anesthetic neurotoxicity in the young: Mechanism and prevention", Neuroscience & Biobehavioral Reviews, 2019
Crossref
15 words — < 1%

24 Yi Wei, Junhua Hu, Yubing Liang, Yuling Zhong, Dan He, Yi Qin, Li Li, Jing Chen, Qiang Xiao, Yubo Xie. "Dexmedetomidine pretreatment attenuates propofol-induced neurotoxicity in neuronal cultures from the rat hippocampus", Molecular Medicine Reports, 2016
Crossref
15 words — < 1%

25 Danfeng Yang, Junwei Su, Yeru Chen, Gang Chen. "The NF-κB pathway: Key players in neurocognitive functions and related disorders", European Journal of Pharmacology, 2024
Crossref
14 words — < 1%

26 Fahad Iqbal, Andrew J. Thompson, Saba Riaz, Marcus Pehar, Tiffany Rice, Naweed I. Syed. "Anesthetics: from modes of action to unconsciousness and neurotoxicity", Journal of Neurophysiology, 2019
Crossref
13 words — < 1%

27 Yan Jin, Song Cai, Yuepeng Jiang, Kai Zhong, Chengping Wen, Yeping Ruan, Lindsey A. Chew, Rajesh Khanna, Zhenghao Xu, Jie Yu. "Tetramethylpyrazine Reduces Epileptogenesis Progression in Electrical Kindling Models by Modulating Hippocampal Excitatory Neurotransmission", ACS Chemical Neuroscience, 2019
13 words — < 1%

Crossref

28	"Handbook of Neurotoxicity", Springer Nature, 2014<br>Crossref	12 words — < 1%
29	"Mitochondrial Biology and Experimental Therapeutics", Springer Science and Business Media LLC, 2018<br>Crossref	12 words — < 1%
30	Biswas, Bharti. "Effect of Combined Early Life Stressors on Brain and Behaviour in Rat Offspring.", University of New South Wales (Australia)<br>ProQuest	12 words — < 1%
31	Annalisa Savardi, Ilias Ziogas, Maria Bolla, Laura Cancedda. "Chloride transporters in physiological brain development and neurodevelopmental disorders: The case of the Down syndrome", Elsevier BV, 2020<br>Crossref	11 words — < 1%
32	Kantarakorn Unchiti, Prangmalee Leurcharusmee, Artid Samerchua, Tanyong Pipanmekaporn et al. "The potential role of dexmedetomidine on neuroprotection and its possible mechanisms: Evidence from in vitro and in vivo studies", European Journal of Neuroscience, 2021<br>Crossref	11 words — < 1%
33	academic.oup.com<br>Internet	11 words — < 1%
34	www.medrxiv.org<br>Internet	11 words — < 1%
35	Andreas W. Loepke. "Developmental neurotoxicity of sedatives and anesthetics: A	10 words — < 1%

concern for neonatal and pediatric critical care medicine?* :", Pediatric Critical Care Medicine, 03/2010
Crossref

36 Dan Zhao, Minli Zhang, Lingling Yang, Mingquan Zeng. "GPR68 Improves Nerve Damage and Myelination in an Immature Rat Model Induced by Sevoflurane Anesthesia by Activating cAMP/CREB to Mediate BDNF", ACS Chemical Neuroscience, 2022 — 10 words — < 1%
Crossref

37 Diogo Dominguini, Amanda V. Steckert, Monique Michels, Mariana B. Spies et al. "The effects of anaesthetics and sedatives on brain inflammation", Neuroscience & Biobehavioral Reviews, 2021 — 10 words — < 1%
Crossref

38 Félicie Lorenc, Luc Dupuis, Raphaelle Cassel. "Impairments of inhibitory neurons in amyotrophic lateral sclerosis and frontotemporal dementia", Neurobiology of Disease, 2024 — 10 words — < 1%
Crossref

39 Laura Castañeda-Martinez, Kevin K. Noguchi, Chrysanthy Ikonomidou, James A. Zagzebski, Timothy J. Hall, Ivan M. Rosado-Mendez. "Optimization of Ultrasound Backscatter Spectroscopy to Assess Neurotoxic Effects of Anesthesia in the Newborn Non-human Primate Brain", Ultrasound in Medicine & Biology, 2020 — 10 words — < 1%
Crossref

40 Lili Xu, Qi Xu, Shaobing Dai, Cuicui Jiao, Yingying Tang, Jiaqian Xie, Hui Wu, Xinzhong Chen. "lncRNA Xist regulates sevoflurane-induced social and emotional impairment by modulating miR-98-5p/EDEM1 signaling axis in neonatal mice", Molecular Therapy - Nucleic Acids, 2021 — 10 words — < 1%
Crossref

41 Shetty, Ashok K. "Studies on Developmental Exposures to Anesthetic Agents and Stem Cell Derived Models", Neural Stem Cells in Health and Disease, 2015.
Crossref
10 words — < 1%

42 Cheng, Yuan, Qiaoying Li, Yidan Zhang, Quan Wen, and Jianjun Zhao. "Effects of Female Sex Hormones on Expression of Ang-(1-7)-Mas-R-nNOS Pathways in Rat Brain", Canadian Journal of Physiology and Pharmacology, 2015.
Crossref
9 words — < 1%

43 E.B. Engler-Chiurazzi, C.M. Brown, J.M. Povroznik, J.W. Simpkins. "Estrogens as neuroprotectants: Estrogenic actions in the context of cognitive aging and brain injury", Progress in Neurobiology, 2017
Crossref
9 words — < 1%

44 Genghua Xu, Nuoya Liu, Yaqing Qiu, Jiayu Qi, Danyan Zhu. "Role of Histamine H3 Receptor Antagonist Pitolisant in Early Neural Differentiation of Mouse Embryonic Stem Cells", Stem Cells and Development, 2023
Crossref
9 words — < 1%

45 Guo, Peipei, Zhibin Huang, Tao Tao, Xiaohui Chen, Wenqing Zhang, Yiyue Zhang, and Chunshui Lin. "Zebrafish as a model for studying the developmental neurotoxicity of propofol : Developmental neurotoxicity of propofol in zebrafish", Journal of Applied Toxicology, 2015.
Crossref
9 words — < 1%

46 Jing Lv, Yubing Liang, Youbing Tu, Jing Chen, Yubo Xie. "Hypoxic preconditioning reduces propofol-induced neuroapoptosis via regulation of Bcl-2 and Bax and downregulation of activated caspase-3 in the hippocampus of neonatal rats", Neurological Research, 2018
Crossref
9 words — < 1%

47 Lijuan Shu, Chunfu Du. "PHLDA1 promotes sevoflurane-induced pyroptosis of neuronal cells in developing rats through TRAF6-mediated activation of Rac1", NeuroToxicology, 2022 — 9 words — < 1%
Crossref

48 Luís F. Martins, Rui O. Costa, Joana R. Pedro, Paulo Aguiar et al. "Mesenchymal stem cells secretome-induced axonal outgrowth is mediated by BDNF", Scientific Reports, 2017 — 9 words — < 1%
Crossref

49 Olutoyin A. Olutoye, Candace Style, Alicia Menchaca. "Neurocognitive Effects of Fetal Exposure to Anesthesia", Anesthesiology Clinics, 2021 — 9 words — < 1%
Crossref

50 Yang Zhang, Shan Liu, Jiu-Long Ma, Chen Chen, Peng Huang, Jia-Hua Ji, Di Wu, Li-Qun Ren. "Apocynum venetum leaf extract alleviated doxorubicin-induced cardiotoxicity through the AKT/Bcl-2 signaling pathway", Phytomedicine, 2021 — 9 words — < 1%
Crossref

51 aopwiki.org — 9 words — < 1%
Internet

52 discovery.ucl.ac.uk — 9 words — < 1%
Internet

53 hal-amu.archives-ouvertes.fr — 9 words — < 1%
Internet

54 opac.lib.idu.ac.id — 9 words — < 1%
Internet

55 "Neonatal Anesthesia", Springer Science and Business Media LLC, 2023
Crossref
8 words — < 1%

56 Abdelhamid, Ramy, Jia Luo, Lawren VandeVrede, Indraneel Kundu, Bradley Michalsen, Vladislav A. Litosh, Isaac T. Schiefer, Teshome Gherezghiher, Ping Yao, Zhihui Qin, and Gregory R. J. Thatcher. "Benzothiophene Selective Estrogen Receptor Modulators Provide Neuroprotection by a Novel GPR30-Dependent Mechanism", ACS Chemical Neuroscience, 2011.
Crossref
8 words — < 1%

57 Anita C. Truttmann, Vanessa Ginet, Julien Puyal. "Current Evidence on Cell Death in Preterm Brain Injury in Human and Preclinical Models", Frontiers in Cell and Developmental Biology, 2020
Crossref
8 words — < 1%

58 Chun-Yun Zhang, Fang-Fang He, Hua Su, Chun Zhang, Xian-Fang Meng. "Association between chronic kidney disease and Alzheimer’s disease: an update", Metabolic Brain Disease, 2020
Crossref
8 words — < 1%

59 Doboszewska, Urszula, Bernadeta Szewczyk, Magdalena Sowa-Kućma, Karolina Noworyta-Sokołowska, Paulina Misztak, Joanna Gołębiowska, Katarzyna Młyniec, Beata Ostachowicz, Mirosław Krośniak, Agnieszka Wojtanowska-Krośniak, Krystyna Gołembiowska, Marek Lankosz, Wojciech Piekoszewski, and Gabriel Nowak. "Alterations of Bio-elements, Oxidative, and Inflammatory Status in the Zinc Deficiency Model in Rats", Neurotoxicity Research, 2015.
Crossref
8 words — < 1%

60	Fei-Fei Ma, Run-Hui Ma, Wang-Wei Zhang, Xiu-Xiu Zhang, Qian Zhong, Kiran Thakur, Jian-Guo Zhang, Jesus Simal-Gandara, Hui Cao. "Neferine induces apoptosis and inhibits metastasis in human endometrial cancer cells by down-regulating miR-1266–5p", Food Bioscience, 2025<br>Crossref	8 words — < 1%
61	Groark, Christina J.; McCarthy, Stephanie K.; Kirk, Afton R. . "Early Childhood Development: From Theory to Practice, Second Edition", UAGC, 2023<br>Publications	8 words — < 1%
62	Hisham F. Bahmad, Batoul Darwish, Karem Bou Dargham, Rabih Machmouchi et al. "Role of MicroRNAs in Anesthesia-Induced Neurotoxicity in Animal Models and Neuronal Cultures: a Systematic Review", Neurotoxicity Research, 2019<br>Crossref	8 words — < 1%
63	Hocker, Austin D., Jennifer A. Stokes, Frank L. Powell, and Adrianne G. Huxtable. "The impact of inflammation on respiratory plasticity", Experimental Neurology, 2016.<br>Crossref	8 words — < 1%
64	Jennifer L. Walters, Merle G. Paule. "Review of preclinical studies on pediatric general anesthesia-induced developmental neurotoxicity", Neurotoxicology and Teratology, 2017<br>Crossref	8 words — < 1%
65	Kokane, Saurabh S., and Qing Lin. "Impact of Early Life Ketamine Exposure on the Developing Brain and Cognitive Sequelae", Neuropathology of Drug Addictions and Substance Misuse, 2016.<br>Crossref	8 words — < 1%

66 Martin Mortensen, Yue Xu, Mohamed A. Shehata, Jacob Krall, Margot Ernst, Bente Frølund, Trevor G. Smart. " Pregnenolone sulfate analogues differentially modulate GABA receptor closed/desensitised states ", British Journal of Pharmacology, 2023
Crossref
8 words — < 1%

67 Mary Ellen McCann, Sulpicio G. Soriano. "General Anesthetics in Pediatric Anesthesia: Influences on the Developing Brain", Current Drug Targets, 2012
Crossref
8 words — < 1%

68 N. Lunardi. "General Anesthesia Causes Long-Lasting Disturbances in the Ultrastructural Properties of Developing Synapses in Young Rats", Neurotoxicity Research, 07/21/2009
Crossref
8 words — < 1%

69 Ngoc Kim Cuong Tran, Ji Hoon Jeong, Naveen Sharma, Yen Nhi Doan Nguyen et al. "Ginsenoside Re blocks Bay k-8644-induced neurotoxicity via attenuating mitochondrial dysfunction and PKCδ activation in the hippocampus of mice: Involvement of antioxidant potential", Food and Chemical Toxicology, 2023
Crossref
8 words — < 1%

70 Oliver Henschel, Keith Gipson, Angelique Bordey. "GABAA Receptors, Anesthetics and Anticonvulsants in Brain Development", CNS & Neurological Disorders - Drug Targets, 2008
Crossref
8 words — < 1%

71 Omar Hoseá Cabrera, Nemanja Useinovic, Stefan Maksimovic, Michelle Near et al. "Neonatal ketamine exposure impairs infrapyramidal bundle pruning and causes lasting increase in excitatory synaptic transmission in hippocampal CA3 neurons", Neurobiology of Disease, 2022
8 words — < 1%

Crossref

72 Shan Lei, Pan Lu, Yang Lu, Juan Zheng et al. "Dexmedetomidine Alleviates Neurogenesis Damage Following Neonatal Midazolam Exposure in Rats through JNK and P38 MAPK Pathways", ACS Chemical Neuroscience, 2020 — 8 words — < 1%
Crossref

73 Vinayak Mishra, Brian Sims, Margaret Kuper-Sassé, Akhil Maheshwari. "Neurodevelopmental Impairment in Specific Neonatal Disorders", Elsevier BV, 2024 — 8 words — < 1%
Crossref

74 Yafang Liu, Daowei Lin, Chuiliang Liu, Yifan Zhao, Zhiwen Shen, Kun Zhang, Minghui Cao, Yujuan Li. "Cyclin-dependent kinase 5/Collapsin response mediator protein 2 pathway may mediate sevoflurane-induced dendritic development abnormalities in rat cortical neurons", Neuroscience Letters, 2017 — 8 words — < 1%
Crossref

75 Youbing Tu, Yubing Liang, Yong Xiao, Jing Lv, Ruicong Guan, Fei Xiao, Yubo Xie, Qiang Xiao. "<p>Dexmedetomidine attenuates the neurotoxicity of propofol toward primary hippocampal neurons in vitro via Erk1/2/CREB/BDNF signaling pathways</p>", Drug Design, Development and Therapy, 2019 — 8 words — < 1%
Crossref

76 Yu Hou, Shuai Li, Qi Hou, Runjia Wang, Xiyuan Xu, Zhichao Li, Linhui Ma, Qidong Liu, Yuan Shen, Hui Zheng. "Vitamin K2 mitigates cognitive, and motor impairments induced by multiple surgery with anesthesia and analgesia in neonatal stage", Biochemical and Biophysical Research Communications, 2025 — 8 words — < 1%
Crossref

77 Yuying Huang, Jingwen Xu, Yihai Wang, Yanqiong Lei, Yongxin Mai, Xiangjiu He. "Q43, a new triterpenoid extracted from Chinese acorn, exhibits pronounced anti-neuroinflammatory activity through the MAPK and NF-κB pathways", Journal of Functional Foods, 2021 — 8 words — < 1%
Crossref

78 Zachary Fleissner, Antonia Schonwald, Neil Patel, Benjamin F. Gruenbaum, Valerie A. Smart, Shaun E. Gruenbaum. "Neurotoxicity and translational research", Elsevier BV, 2022 — 8 words — < 1%
Crossref

79 Zhi-Li Yao, Qing-Nan Li, Yong-Ji Li, Hao-Rui Li et al. "CRISPR/Cas12a-based fluorescence-colorimetric dual-mode strategy for sensitive detection of clear cell renal cell carcinoma biomarker miR-210–3p", Sensors and Actuators B: Chemical, 2025 — 8 words — < 1%
Crossref

80 d.docksci.com — 8 words — < 1%
Internet

81 journals.lww.com — 8 words — < 1%
Internet

82 openrepository.aut.ac.nz — 8 words — < 1%
Internet

83 qspace.library.queensu.ca — 8 words — < 1%
Internet

84 rcastoragev2.blob.core.windows.net — 8 words — < 1%
Internet

85 su.diva-portal.org — 8 words — < 1%
Internet

86 wiredspace.wits.ac.za
Internet
8 words — < 1%

87 www.biorxiv.org
Internet
8 words — < 1%

88 www.grafiati.com
Internet
8 words — < 1%

89 A. Leslie Morrow, Minna H. McFarland, Todd K. O'Buckley, Donita L. Robinson. "Emerging evidence for pregnane steroid therapeutics for alcohol use disorders", Elsevier BV, 2024
Crossref
7 words — < 1%

90 Baptiste Riffault, Igor Medina, Camille Dumon, Carine Thalman et al. " Pro-Brain-Derived Neurotrophic Factor Inhibits GABAergic Neurotransmission by Activating Endocytosis and Repression of GABA Receptors ", The Journal of Neuroscience, 2014
Crossref
7 words — < 1%

91 Guiping Xu, Yang Wang, Zhe Chen, Yuxuan Zhang, Xuexue Zhang, Guichao Zhang. "Esketamine improves propofol-induced brain injury and cognitive impairment in rats", Translational Neuroscience, 2022
Crossref
7 words — < 1%

92 Hewitt, Danielle. "Neural Changes Associated with Spinal Cord and External Peripheral Nerve Stimulation for the Treatment of Neuropathic Lower Limb Pain", The University of Liverpool (United Kingdom), 2023
ProQuest
7 words — < 1%

93 Ian Coulter, Tamara Timic Stamenic, Pierce Eggan, Brier R. Fine et al. "Different roles of T-type calcium
7 words — < 1%

channel isoforms in hypnosis induced by an endogenous neurosteroid epipregnanolone", Neuropharmacology, 2021
Crossref

94 Kok, Morgan. "A Molecular and Computational Approach to Analyze the Biogenesis of the Potassium-Chloride Cotransporter 2 (KCC2), A Neuron-Specific Protein Implicated in Disease", University of Pittsburgh — 7 words — < 1%
ProQuest

95 Nan Wang, Mingyu Wang. "Dexmedetomidine suppresses sevoflurane anesthesia-induced neuroinflammation through activation of the PI3K/Akt/mTOR pathway", BMC Anesthesiology, 2019 — 7 words — < 1%
Crossref

96 Refik Servi, Ramazan Fazıl Akkoç, Feyza Aksu, Süleyman Servi. "Therapeutic potential of enzymes, neurosteroids, and synthetic steroids in neurodegenerative disorders: A critical review", The Journal of Steroid Biochemistry and Molecular Biology, 2025 — 7 words — < 1%
Crossref

97 Stephanie L. Merhar, Carla M. Bann, Nicole Mack, Jamie E. Newman et al. "Prenatal Opioid Exposure is Associated with Punctate White Matter Lesions in Term Newborns", The Journal of Pediatrics, 2025 — 7 words — < 1%
Crossref

98 "Fundamentals of Pediatric Neuroanesthesia", Springer Science and Business Media LLC, 2021 — 6 words — < 1%
Crossref

99 "Total Intravenous Anesthesia and Target Controlled Infusions", Springer Science and Business Media LLC, 2017 — 6 words — < 1%
Crossref

100 A. Leslie Morrow, Giorgia Boero, Irina Balan. "Emerging Evidence for Endogenous Neurosteroid Modulation of Pro-Inflammatory and Anti-Inflammatory Pathways that Impact Neuropsychiatric Disease", Neuroscience & Biobehavioral Reviews, 2024
Crossref
6 words — < 1%

101 Hostetler, Rachel Elaine. "The Diversity and Synaptic Connectivity of Somatostatin-containing Inhibitory Interneurons in the Somatosensory (Barrel) Cortex", West Virginia University, 2024
ProQuest
6 words — < 1%

102 Jiaxi Qian, Maorong Jiang, Zihan Ding, Dandan Gu, Huiyuan Bai, Min Cai, Dengbing Yao. "Role of Long Non-coding RNA in Nerve Regeneration", International Journal of Neuroscience, 2023
Crossref
6 words — < 1%

103 Ryan A. Stevens, Brandon D. Butler, Saurabh S. Kokane, Andrew W. Womack, Qing Lin. "Neonatal inhibition of Na + -K + -2Cl − -cotransporter prevents ketamine induced spatial learning and memory impairments", Neurotoxicology and Teratology, 2017
Crossref
6 words — < 1%

104 Wanping Gu, Tingting Pan, Xuedong Wang, Liheng Kang, Nan Liu, Meihua Piao, Chunsheng Feng. "Sevoflurane exposure triggers ferroptosis of neuronal cells initiated by the activation of ATM/p53 in the neonatal brain via JNK/p38 MAPK-mediated oxidative DNA damage", Springer Science and Business Media LLC, 2024
Crossref Posted Content
6 words — < 1%

105 X. Zhang, F. Liu, W. Slikker, C. Wang, M.G. Paule. "Minimally invasive biomarkers of general
6 words — < 1%

anesthetic-induced developmental neurotoxicity",
Neurotoxicology and Teratology, 2017
Crossref

EXCLUDE QUOTES ON
EXCLUDE BIBLIOGRAPHY ON
EXCLUDE SOURCES OFF
EXCLUDE MATCHES OFF
